# Topical paromomycin for New World cutaneous leishmaniasis

**DOI:** 10.1371/journal.pntd.0007253

**Published:** 2019-05-02

**Authors:** Néstor Sosa, Juan Miguel Pascale, Ana I. Jiménez, Jeanne A. Norwood, Mara Kreishman-Detrick, Peter J. Weina, Kendra Lawrence, William F. McCarthy, Ryan C. Adams, Charles Scott, Janet Ransom, Douglas Tang, Max Grogl

**Affiliations:** 1 Instituto Conmemorativo Gorgas de Estudios de la Salud, Avenida Justo Arosemena, Panama City, Panama; 2 US Army Medical Materiel Development Activity (USAMMDA), Fort Detrick, MD, United States of America; 3 Walter Reed Army Institute of Research, Silver Spring, MD, United States of America; 4 Walter Reed National Military Medical Center, Bethesda, MD, United States of America; 5 Fast-Track Drugs and Biologics, North Potomac MD, United States of America; 6 US Naval Medical Research Unit No. 6, in Lima, Peru; Instituto de Ciências Biológicas, Universidade Federal de Minas Gerais, BRAZIL

## Abstract

**Background:**

Paromomycin-based topical treatments were shown to be effective in curing cutaneous leishmaniasis (CL) lesions caused by *Leishmania major* in Tunisia. Cure rates of an index lesion were approximately 80%. As a follow on, we conducted a similar Phase 3 trial in Panama to demonstrate the efficacy of these treatments against New World species. The primary objective was to determine if a combination topical cream (paromomycin-gentamicin) resulted in statistically superior final clinical cure rates of an index lesion compared to a paromomycin alone topical cream for the treatment of CL, primarily caused by *Leishmania panamensis*.

**Methods:**

We conducted a randomized, double blind, Phase 3 trial of topical creams for the treatment of CL caused by *Leishmania* spp. Three hundred ninety nine patients with one to ten CL lesions were treated by topical application once daily for 20 days. The primary efficacy endpoint was percentage of subjects with clinical cure of an index lesion confirmed to contain *Leishmania* with no relapse.

**Results:**

The clinical cure of the index lesion for paromomycin-gentamicin was 79% (95% CI; 72 to 84) and for paromomycin alone was 78% (95% CI; 74 to 87) (p = 0.84). The most common adverse events considered related to study cream application were mild to moderate dermatitis, pain, and pruritus.

**Conclusions:**

Superiority of paromomycin-gentamicin was not demonstrated. However, the approximately 80% cure rates for both topical creams were similar to those demonstrated in Tunisia and previously reported with parenteral antimonials.

## Author summary

Leishmaniasis, a neglected parasitic infection transmitted by the bite of a female sand fly, is endemic in 98 countries or territories with approximately 0.7 to 1.2 million cutaneous leishmaniasis (CL) cases occurring each year. In Panama, most of the CL cases are caused by *L*. *panamensis* and, the first line of treatment is pentavalent antimony, given parenterally for 20 days. These systemic regimen is associated with toxicities that can limit the patient from receiving a full course of treatment. Alternative therapies are needed particularly for patients with mild disease, no mucosal involvement, no immunosuppression, and for patients living in areas with scarce infrastructure. Therefore, less toxic, non-parenteral new therapies against CL are urgently needed. We conducted a comparative clinical study that evaluated Paromomycin topical creams (Paromomycin alone versus Paromomycin+Gentamicin) for the treatment of cutaneous leishmaniasis (n = 399) in three sites of country. Our study demonstrated the efficacy of these preparations against New World leishmanial species (mostly *L*. *panamensis*) with a cure rate close to 80%.

Our trial supports Paromomycin as a viable alternative treatment for CL caused for the New World *Leishmania* species.

## Introduction

Leishmaniasis, a neglected parasitic infection transmitted by the bite of a female sand fly, is endemic in 98 countries or territories with approximately 0.7 to 1.2 million cutaneous leishmaniasis (CL) cases occurring each year [1]. CL results from parasitisation of skin macrophages by *Leishmania (L)* species and generally presents as a papule that enlarges to a nodule that often ulcerates over 1–3 months [2]. The illness has a variety of skin manifestations including small, dry, crusted lesions; ulcerative lesions that are shallow and circular with well-defined borders and a bed of granulated tissue; and large, deep, mutilating ulcers [3]. There are at least five *Leishmania* species that cause CL in the Old World and 12 species in the New World [4]. Also, in Panama, leishmaniasis is an important parasitic disease with an average estimated 2,200 new cases of CL reported per year, although this number is likely a 4-fold underestimate due to underreporting [1,5]. Among the cases reported in Panama from 2005–2009 the majority were diagnosed as *L*. *panamensis*. *L*. *panamensis* typically causes CL lesions [1,6]. However, it does have the potential to progress to mucocutaneous leishmaniasis in approximately 5% of cases [3]. CL can create substantial morbidity due to the continued presence of a skin ulcer and the psychological impact of disfigurement [7].

The first line of treatment for CL in Panama is pentavalent antimony, either meglumine antimoniate or sodium stibogluconate, given parenterally for 20 to 28 days [8]. The cure rates for *L*. *panamensis* CL in adults treated with systemic antimony have been reported in the range from 25–93% [9,10]. However, these systemic regimens are associated with toxicities that can limit the patient from receiving a full course of treatment [11]. Alternative therapies are needed particularly for patients with mild disease, no mucosal involvement, and who are not immunocompromised, and for patients living in areas with scarce infrastructure (most CL endemic areas) where laboratory monitoring and trained health personnel needed for correct management of pentavalent antimony (SbV) treatments are limited. Children also represent a large part of the affected population in Panama and in other endemic regions and there is evidence that pediatric patients with CL have a significantly lower response rate to pentavalent antimonials [8,12]. In the registry of leishmaniasis cases from the Ministry of Health-Panama in 2014, 77% of those affected were 19 years of age or younger and 60% were under 10 years of age [8].

Therefore, the availability of a topically applied drug, instead of a parenteral therapy, offers a potentially safer and more easily administered treatment.

In the Phase 3 study in Tunisia, 15% paromomycin-0.5% gentamicin (called WR 279,396) topical cream and 15% paromomycin alone topical cream were equally effective but statistically superior to a vehicle control (paromomycin-gentamicin, paromomycin alone, and vehicle-control final clinical cure rates of 81%, 82% versus 58%, P<0.001, respectively) [13]. However, results from a mouse study suggested gentamicin would provide an added benefit in New World *Leishmania* species [14]. In addition, a Phase 2 study conducted in Panama showed a trend toward superiority of paromomycin-gentamicin over paromomycin alone against *L*. *panamensis* [15]. The results of these studies were supportive of testing the superiority of paromomycin-gentamicin over that of paromomycin alone in the New World CL caused by *L*. *panamensis*.

## Methods

### Ethics statement

The protocol was approved by the Gorgas Institutional Bioethics Committee, the National Committee of Bioethics for Research, Panama and by the Human Research Protections Office, U.S. Army Medical Research and Materiel Command. All patients or their legal representatives provided written informed consent, and minors also provided assent.

### Study design

This study was a pivotal Phase 3, randomized, double-blind, two-group trial assessing the efficacy and safety of paromomycin-gentamicin and paromomycin alone topical cream in a hydrophilic vehicle (designed to enhance drug penetration while maintaining high tolerability) in subjects with CL in Panama. A vehicle-control group was not included as it was considered unethical to withhold treatment based on the standard of care in Panama and the results of the Phase 3 Tunisian study, which showed the statistical superiority of paromomycin-gentamicin and paromomycin alone compared with the vehicle-control [13].

This study was conducted between May 2013 and March 2016 at three sites in Panama: Penonomé, Panama City, and Changuinola.

### Treatments

The same investigational products were used in Tunisia and in this study. Paromomycin-gentamicin and paromomycin alone in a hydrophilic vehicle were manufactured by Teva Pharmaceuticals USA, Sellersville, Pennsylvania in accordance with Good Manufacturing Practices. For each patient, all lesions (i.e., the index lesion, as defined below, plus non-index lesions) were treated topically once daily for 20 days by a member of the study staff who documented treatment application. (For details of the application procedure, see Supporting information, S2 Appendix.)

### Study patients

Study subjects were males or non-pregnant non-lactating females, ages 2 and older, and with 10 or fewer lesions. For each subject, an index lesion was selected with the following characteristics: ulcerative, from 1–5cm in diameter, and confirmed to contain *Leishmania* parasites via culture or microscopic examination of lesion material. Subjects were otherwise healthy and without clinical evidence of mucosal involvement. Whenever possible, infecting species of *Leishmania* were determined by polymerase chain reaction (PCR) followed by restriction fragment length polymorphism (RFLP) using the heat shock protein 70 for discrimination of *Leishmania* species [16] and isoenzyme analysis [multilocus enzyme electrophoresis (MLEE)][17]. Eligibility criteria and study procedures are described in the Supporting information, S2 Appendix.

### Endpoints

#### Efficacy

The primary efficacy endpoint was percentage of subjects with final clinical cure. Final clinical cure was defined as: initial clinical cure (100% re-epithelialization of index lesion by nominal.

Day 63) or initial clinical improvement (greater than 50% re-epithelialization of index lesion by nominal Day 63) followed by 100% re-epithelialization of the index lesion on or before nominal Day 100. In addition, no relapse of index lesion by nominal Day 168.

Key secondary efficacy endpoints were: percentage of subjects with all lesions cured, defined as final clinical cure (as defined above) and cure of all other lesions by nominal Day 100 (100% re-epithelialization of all ulcerated lesions and resolution of all other types of lesions); and median time to initial clinical cure (100% re-epithelialization of the index lesion).

The percentage of subjects meeting criteria for final clinical cure of index lesion was also analyzed by age group and infecting species of *Leishmania*.

#### Safety

The safety endpoints were adverse events (AE) including application site reactions (pain, erythema, edema, and vesicles) and increased creatinine and transaminases. Examination of the nasal and oral mucosa was performed at baseline and Days 63, 100, and 168 for evidence of mucosal disease. Evidence of mucosal leishmaniasis was also considered an adverse event.

### Statistical analysis

The original sample size was 300 subjects. During the conduct of this trial, new *Leishmania* species were identified that were not found in the Phase 2 trial previously performed in Panama. Of 149 subjects randomized to the trial for which speciation data were available, 74% of subjects had *L*. *panamensis* and the others were split between *L*. *guyanensis* and *L*. *braziliensis*. Since it was not clear what impact various species may have had on overall treatment effect, it was felt that a more conservative approach to trial power was needed. The sample size of the study was adjusted to 400 total subjects, adding 50 subjects to each study arm to maintain at least 90% power with a two-sided alpha of 0.05 to detect statistically significant superiority of paromomycin-gentamicin over paromomycin alone for *L*. *panamensis* patients.

The modified intention-to-treat (mITT) population (N = 399) and the safety population consisted of all subjects who received any administration of investigational product and was used as the primary analytic population for efficacy and safety analyses. The evaluable population (N = 387) included all subjects who received daily doses of investigational product for at least 18 of the total 20 days and did not have missing lesion measurements at Day 63 and 168.

Final clinical cure rates of the index lesion and all lesions (proportions) were compared between the two treatment groups by uncorrected chi-square test using the mITT group.

The Kaplan Meier product-limit method was used to determine the median time to initial clinical cure. The time to initial clinical cure curves were compared using the log-rank test. Within subgroup level two treatment groups were compared by uncorrected chi-square test using the mITT group. A Cochran-Mantel-Haenszel was used to test if there were treatment differences across the subgroup levels.

A two-sided alpha of 0.05 was used to demonstrate statistical significance. No adjustments were made to correct for multiplicity of comparisons of secondary efficacy endpoints. (The complete Statistical Analysis Plan is provided in Supporting information, S3 Appendix).

## Results

**Fig 1:**
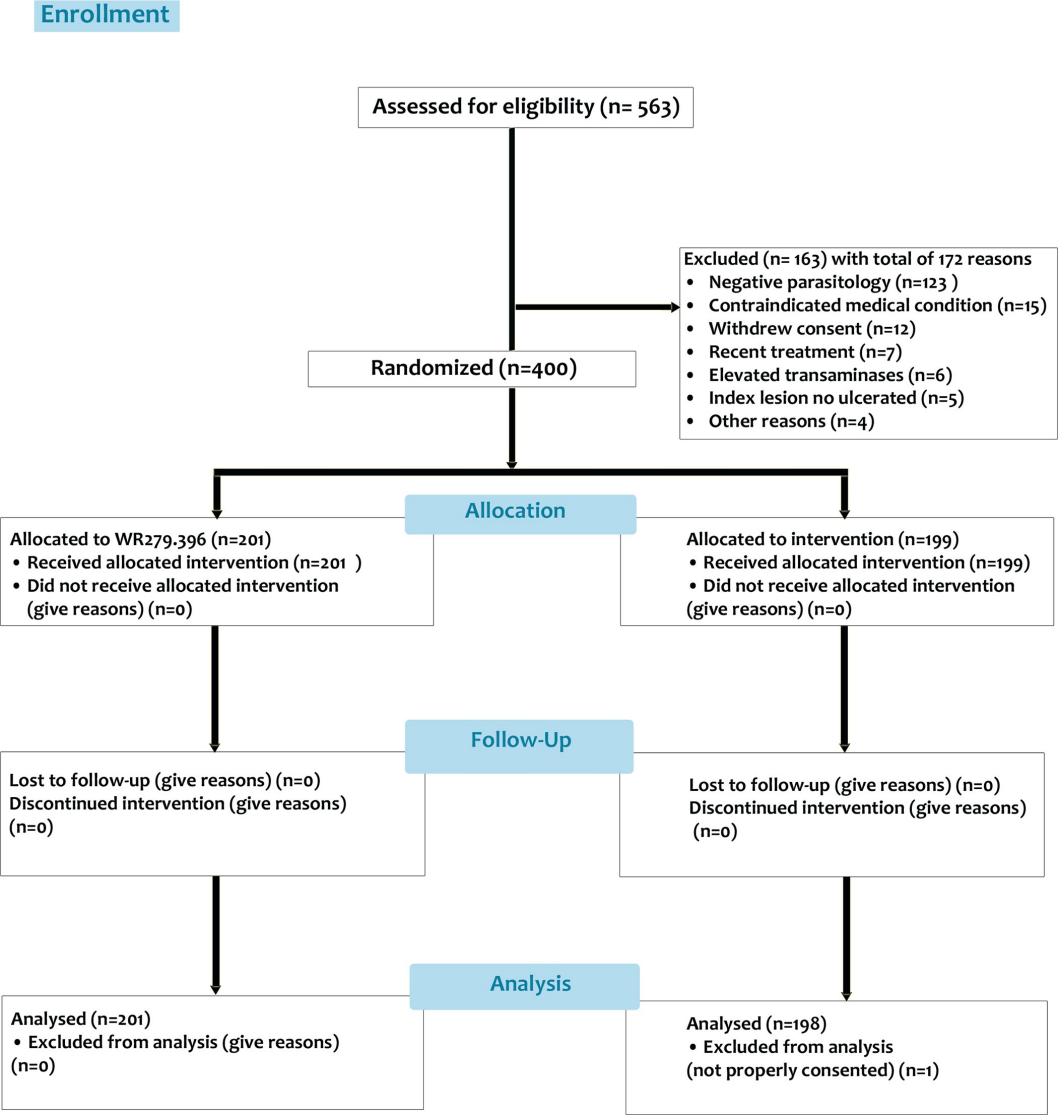
Patient disposition. A total of 563 patients were consented and screened, 400 were randomized and, 163 were not eligible. One randomized patient (a minor) was later determined to not be properly consented (legal guardian could not provide documentation) and was not included in the analysis.

Of 563 subjects screened, 400 were randomized and received an investigational product (Fig 1).

Patient characteristics are shown in Table 1. There was a slightly higher proportion of males (62.7%) than females and adults constituted 53.9% of patients studied. The average number of lesions per patient was 2.2 with areas ranging from 0.2–1158 mm^2^. Age of lesions ranged from 10 to 559 days prior to start of treatment. Baseline characteristics were not significantly different between groups (Table 1).

**Table 1:** Patient and disease characteristics at baseline.

Characteristic	Paromomycin-Gentamicin N = 201	Paromomycin N = 198	All Subjects N = 399
Male Gender: no. (%)	125 (62)	125 (63)	250 (63)
Age (years): mean ± SD (range)	23 ± 17 (2–78)	24 ± 15 (2–73)	23 ± 16 (2–78)
>17 yrs: no. (%)	105 (52)	110 (56)	215 (54)
Total Number of lesions	417	396	813
Area of all lesion ulcers (mm^2^): mean ± SD (range)	120 ± 146 (0.2–1053)	121 ± 152 (5.2–1158)	120 ± 149 (0.2–1158)
Lesions per subject: mean ± SD (range)	2.3 ± 1.7 (1–10)	2.1 ± 1.6 (1–9)	2.2 ± 1.7 (1–10)
Lesion age (days): mean ± SD (range)	59.7 ± 53.6 (15–374)	62.1 ± 57.7 (10–559)	60.9 ± 55.6 (10–559)
Infecting species: no. (%) *L*. *panamensis L*. *guyanensis L*. *braziliensis L*. *naiffi* Not identified	155 (77.1) 36 (17.9) 5 (2.5) 1 (0.5) 4 (2.0)	146 (73.7) 42 (21.2) 3 (1.5) 0 (0.0) 7 (3.5)	301 (75.4) 78 (19.5) 8 (2.0) 1 (0.3) 11 (2.8)

### Treatment compliance

Of the 399 patients who received treatment, a total of 16 subjects, 9 in the paromomycin-gentamicin group and 7 in the paromomycin alone Group missed at least 1 day of application of investigational product. Two patients missed treatment due to adverse events of mild and transient hypoacusia or vomiting, neither of which were considered to be related to study cream.

### Efficacy

**Fig 2:**
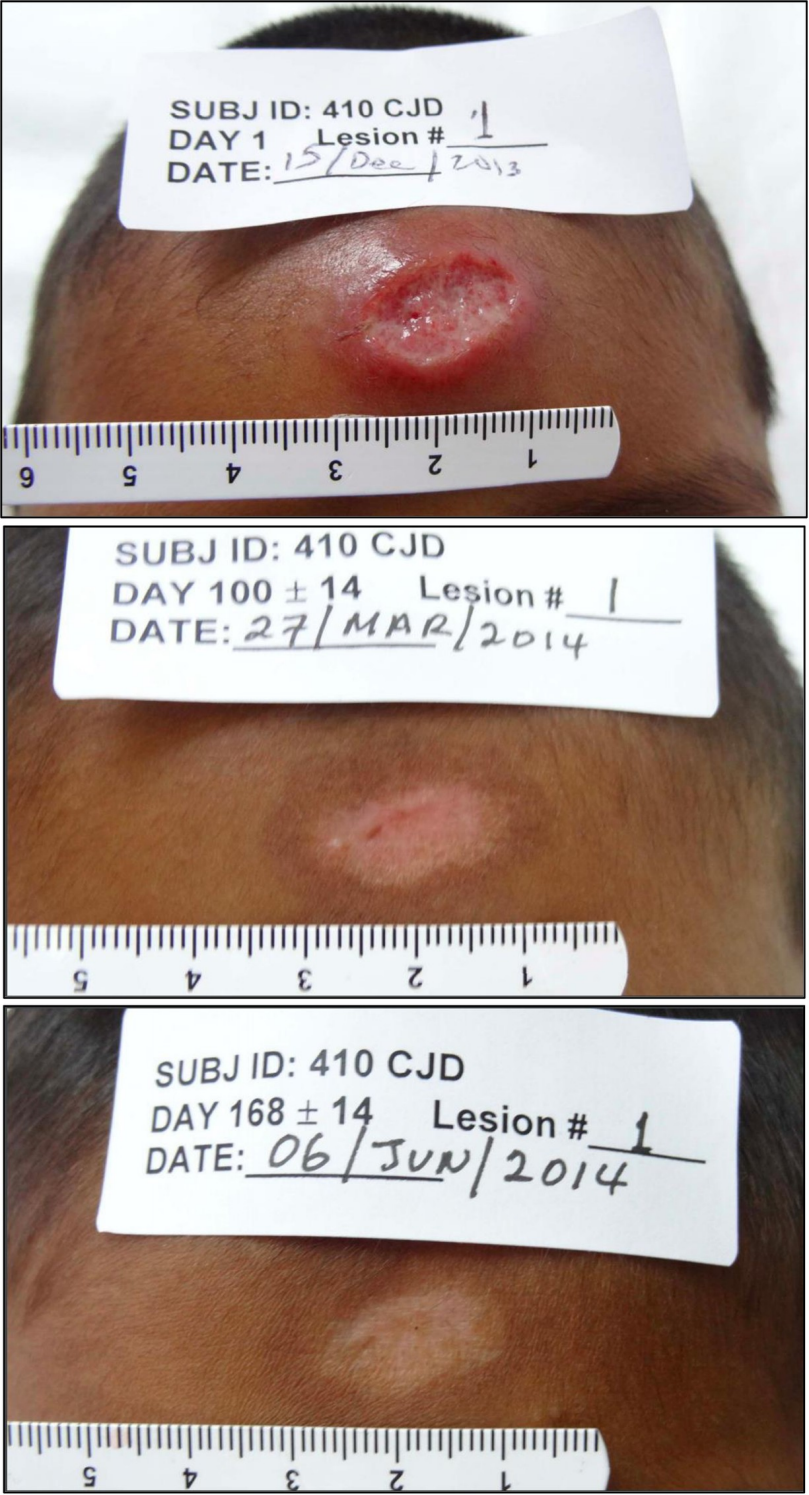
Photographs of lesion #1 in a subject treated with paromomycin over time (baseline, day 100 and day 168).

There was no significant difference in the primary efficacy endpoint, final clinical cure rate of an index lesion, between groups (79% vs. 78% of subjects; paromomycin-gentamicin vs. paromomycin alone; p = 0.84, a difference of -0.83% (95% CI -8.93 to 7.27) (Table 2). The evaluable population showed similar results (81% vs. 80% of subjects; paromomycin-gentamicin vs. paromomycin alone; p = 0.94). The typical response of a treated lesion is shown in Fig 2.

**Table 2:** Efficacy outcomes.

Outcome	Paromomycin-Gentamicin	Paromomycin Alone	p-value*
Patients with final clinical cure of index lesion (mITT): no./total (%)	158/201 (78.6)*^†^	154/198 (77.8)*	0.841
Patients with final clinical cure of all lesions (PP): no. /total (%)	157/195 (80.5)	154/192 (80.2)	0.940
Patients with final clinical cure of all lesions (mITT): no. /total (%)	151/201 (75.1)	151/198 (76.3)	0.791
Patients with final clinical cure of all lesions (PP): no. /total (%)	150/195 (76.9)	151/192 (78.6)	0.684
Patients with final clinical cure of index lesion (mITT) by species: no. /total (%)			
*L*. *panamensis*	123/159 (77.4)	118/153 (77.1)	0.961
*L*. *guyanensis*	34/42 (81.0)	29/36 (80.6)	0.965
*L*. *braziliensis*	2/3 (66.7)	5/5 (100.0)	0.168
Patients with final clinical cure of index lesion (mITT) by patient age: no. /total (%)			
Under 12 years	48/61 (78.7)	42/46 (91.3)	0.077
12 to 17 years	31/35 (88.6)	32/42 (76.2)	0.161
Over 17 years	79/105 (75.2)	80/110 (72.7)	0.675
Reasons for early discontinuation	**N = 201**	N = 199**	
Improperly consented minor	0 (0.0)	1 (0.5)	
Lost to follow-up	1 (0.5)	0 (0.0)	
Withdrawal of consent by subject	3 (1.5)	4 (2.0)	
Withdrawal of subject by investigator	1 (0.5)	0 (0.0)	
Continued presence of disease	13 (6.5)	20 (10.1)	
Worsening of disease	4 (2.0)	4 (2.0)	
Recurrence of disease	12 (6.0)	7 (3.5)	
Reasons for clinical failure (documented failure)	N = 43	N = 44	
No initial clinical cure by Day 63	10 (23.3)	16 (36.4)	
No cure by Day 100	4 (9.3)	7 (15.9)	
Disease recurred	20 (46.5)	12 (27.3)	
No clinical response at Day 49		1 (2.3)	
Disease worsened at Day 35		1 (2.3)	
Other reasons for clinical failure			
Withdrawn–due to new multiple lesions	3 (7.0)		
Withdrawn—developed mucosal disease	1 (2.3)	3 (6.8)	
Withdrew consent	3 (7.0)	4 (9.1)	
Withdrawn by investigator	1 (2.3)		
Lost to follow-up	1 (2.3)		

* Uncorrected chi-square test (2-sided).

** Improperly consented minor was not included in any analyses.

Of the 87 subjects in both groups who were final clinical failures of the index lesion, 71 (34 in the paromomycin-gentamicin group and 37 in the paromomycin alone group) had documented clinical failure (disease persistence, disease worsening, or disease relapse), and the other 16 were failures due to withdrawal of consent or lost to follow-up without meeting the protocol definition of failure to cure. The primary reasons for failure in both groups were lack of initial clinical response of disease by Day 100 (49 subjects) or recurrence of disease (32 subjects). There was no significant difference in the percentage of subjects with all lesions cured between groups in the mITT (75% vs. 76%; paromomycin-gentamicin vs. paromomycin alone; p = 0.79, a difference of 1.14% (95% CI -7.28 to 9.55) nor in the evaluable population (77% vs. 79%; paromomycin-gentamicin vs. paromomycin alone; p = 0.68, a difference of 1.72% (95% CI -6.56 to 10.00). Although the final clinical cure rate was the same between the two treatment groups, the median time to initial clinical cure of the index lesion was 36 days (95% CI 35 to 49) for paromomycin-gentamicin and 48 days (95% CI 36 to 49) for paromomycin alone. However, this difference was not significant (p = 0.22) (Table 2).

There were no significant differences in the final clinical cure rate of the index lesion by age group, under 12 years; 12–17 years; and over 17 years (p = 0.92).

Of 399 subjects, 398 were typed using PCR/RFLP. Of those, a total of 312 (78%) of subjects were identified as infected with *L*. *panamensis*, 78 (20%) with *L*. *guyanensis*, and 8 (2%) with *L*. *braziliensis*. There was no significance difference in the final clinical cure rate between treatment groups for any of the species identified (Table 2).

### Safety

All of the AEs were either mild (98.5%) or moderate (1.5%) in severity and none were severe or life-threatening. Adverse events that occurred with at least 5% incidence in any group are shown in Table 3. Application site reactions constituted the majority of AEs considered at least possibly related to investigational products. In order of frequency, these were application site dermatitis, pruritus, erythema, pain, and burning sensation.

**Table 3:** Adverse events occurring in greater than 5% of patients in any study group.

Adverse Event Preferred Term	WR 279,396 (n = 201)	Paromomycin (n = 198)
Contact Dermatitis	90 (44.8)	89 (44.9)
Nasopharyngitis	90 (44.8)	82 (41.4)
Pruritus	54 (26.9)	50 (25.3)
Headache	28 (13.9)	19 (9.6)
Lymphangitis	23 (11.4)	14 (7.1)
Skin erosion	22 (10.9)	11 (5.6)
Bacterial Superinfection	19 (9.5)	19 (9.6)
Application site injury	19 (9.5)	34 (17.2)
Application site dermatitis	19 (9.5)	13 (6.6)
Arthropod bite	17 (8.5)	14 (7.1)
Application site pain	14 (7.0)	16 (8.1)
Rhinitis	12 (6.0)	13 (6.6)
Application site pruritus	11 (5.5)	8 (4.0)
Folliculitis	7 (3.5)	13 (6.6)
Lymphadenopathy	6 (3.0)	10 (5.1)

Eleven subjects (2.8%, 95% CI 1.1–4.4) developed nasal mucosal lesions that were positive for *Leishmania* by histopathology, culture, or PCR. Four of these cases were in the paromomycin-gentamicin group and 7 were in the paromomycin alone group. Mucosal lesions were mild in all cases, with erythema and superficial ulcerations in most of the patients. No septal perforation or nasal deformity was detected. In 9 of the 11 cases, subjects were treated with meglumine antimoniate and all nasal lesions resolved. The other two subjects were lost to follow up.

## Discussion

Neither the primary nor secondary efficacy endpoints provided evidence for the superiority of paromomycin-gentamicin over paromomycin alone for the treatment of CL in Panama. Rather, the results of this study mirror the results of the Phase 3 study in Tunisia, which demonstrated virtually identical final clinical cure rates for both topical creams. Phase 3 clinical trial results against epidemiologically important Old World and New World species make it clear that the addition of gentamicin to paromomycin topical cream provides no additional clinical benefit [13].

Children had a favorable response to the paromomycin investigational products. The percentage of subjects under 12 years and 12 to 17 years of age who achieved final clinical cure rate of the index lesion was 84% and 82%, respectively. A high cure rate in children with a topical treatment is important because of poor adherence and higher rates of metabolic elimination with parenteral antimonials both of which contribute to lower cure rates [18].

Neither of the two topical creams was associated with any serious or severe systemic toxicity. Specifically, no aminoglycoside-related nephrotoxicity or ototoxicity was observed. Application site reactions associated with the topical therapy and contact dermatitis related to the tape dressing were common. However, no patient had to withdraw from the study because of these local adverse events. These findings were in agreement with those reported in other studies [13].

The significance of mucosal lesions detected in eleven subjects is unclear, but it warrants evaluation of the nasal mucosa in all patients with New World CL caused by the subgenus *Viannia*, in order to consider whether systemic therapy is appropriate. To date there is no conclusive evidence that systemic treatment of CL prevents the development of mucosal leishmaniasis [19,20].

This trial demonstrates that topical therapy with a paramomycin-based cream offers a potential alternative to the current standard of care for the treatment of CL in Panama. A topical therapy offers possible advantages over systemic treatments, such as pentavalent antimonials, and might be an alternative to treat CL in children or in settings where parenteral therapy is not feasible. Topical treatment could also be studied in future trials as part of combination treatment with oral or parenteral agents. A topical treatment for uncomplicated CL is also of great interest in the United States, where current treatment options all have suboptimal risk/benefit profiles for this disease.

## Supporting information

S1 Consort Checklist (DOC)

S1 Appendix Trial registration number. (PDF)

S2 Appendix Complete protocol. (DOCX)

S3 Appendix Statistical analysis plan. (PDF)
