# Supplementary material for: Topical paromomycin for New World cutaneous leishmaniasis
Source: PLoS Negl Trop Dis. 2019 May 2;13(5):e0007253. doi: 10.1371/journal.pntd.0007253 (PMC6497224; doi:10.1371/journal.pntd.0007253)
Supplement: S1 Appendix — (PDF) [file pntd.0007253.s002.pdf]

# ClinicalTrials.gov Search Results 11/12/2018

| | Title | Status | Study Results | Conditions | Interventions | Locations |
|---|---|---|---|---|---|---|
| 1 | Phase 3 Study of Walter Reed (WR) 279,396 and Paromomycin Alone for the Treatment of Cutaneous Leishmaniasis in Panama | Completed | Has Results | •Cutaneous Leishmaniasis | •Drug: WR 279,396<br>•Drug: Paromomycin | •Instituto Conmemorativo Gorgas de Estudios de la Salud,, Panama City, Panama |

U.S. National Library of Medicine | U.S. National Institutes of Health | U.S. Department of Health & Human Services
