# Supplementary material for: Topical paromomycin for New World cutaneous leishmaniasis
Source: PLoS Negl Trop Dis. 2019 May 2;13(5):e0007253. doi: 10.1371/journal.pntd.0007253 (PMC6497224; doi:10.1371/journal.pntd.0007253)

Title Page

A Randomized, Double-blind, Pivotal Phase 3 Study of WR 279,396 (Paromomycin + Gentamicin Topical Cream) and Paromomycin Alone Topical Cream for the Treatment of Cutaneous Leishmaniasis in Panama

| Sponsor | The Surgeon General, Department of the Army |
| --- | --- |
| Sponsor’s Representative | Kenneth A. Bertram, MD, PhD US Army Medical Research and Materiel Command (USAMRMC) 504 Scott StreetFt. Detrick, MD 21702-5012  301-619-4517-0317; DSN: 343-4517 Fax: 301-619-2982  Email: usarmy.detrick.medcom-usammda.mbx.usamrmc-regulatory-affairs@mail.mil |
| **Sponsor's Medical Expert for the Trial** | Dr. Alejandro Llanos-Cuentas Instituto de Medicina Tropical Alexander von Humboldt Universidad Peruana Cayetano Heredia Av. Honorio Delgado 430, San Martín de Porras Lima, Peru Tel: +51-1-482-7739 |
| **Sponsor's Scientific Expert for the Trial** | Max Grögl, PhD United States Army Medical Material Development Activity (USAMMDA) 1430 Veterans Drive, Fort Detrick, MD 21702-5009 Tel: +1 410-564-7089 Email: max.grogl@gmail.com |
| **Research Monitor** | Maria Eugenia Barnett de Antinori, MD Regional Training Center Gorgas Memorial Institute Avenida Justo Arosemena Entre Calles 35 y 36 Panama City, Panama Tel: +507-6615-1338 E-mail: mebantinori@cwpanama.net |
| Principal Investigator | Néstor Sosa, MD FACP Instituto Conmemorativo Gorgas de Estudios de la Salud Avenida Justo Arosemena Panama City, Panama Tel: +507-527-4890 E-mail: drnsosa@gmail.com |
| Subinvestigators: | Dr. José Calzada, PhD Juan Miguel Pascale, MD Ana Jiménez, MD Aracelis Miranda, MSc Zeuz Capitan-Barrios, MSc Vanessa Almengor Franz Barnes, MD Lourdes Kirschenbaum, MD Robert Samuels, MD, ENT Betsy Ortiz, RN Azael Saldaña, PhD Kadir González Jorge Tuñon, MD Suzette Chacón Allin Lewis Rodileida Castillo, RN Xochilt Rugmama, RN Maurice Coronado, MD Laureano Aguilar, MD Elvia Valdés, RN Naty Aguirre, RN Angélica Pedrouzo José Velásquez Instituto Conmemorativo Gorgas de Estudios de la Salud Avenida Justo Arosemena Entre Calle 35 y 36 Panama City, Panama Tel: +507-527-4890 |
| Clinical Trial Sites | Instituto Conmemorativo Gorgas de Estudios de la Salud Avenida Justo Arosemena Entre Calle 35 y 36 Panama City, Panama  Federal Wide Assurance No.: FWA00005923  Hospital Aquilino Tejeira Penonome, Cocle, Panama  Centro Materno Infantil Sandra Hernandez El Silencio, Bocas del Toro, Panama |
| Qualified Physicians Responsible for All Trial-Site-Related Medical Decisions: | Néstor Sosa, MD FACP  Ana Jiménez, MD Instituto Conmemorativo Gorgas de Estudios de la Salud Avenida Justo Arosemena Panama City, Panama Tel: +507-527-4890 |
| Clinical Laboratories and Other Departments/Institutions Involved in the Trial | |
| Site Investigational Product Accountability | Néstor Sosa, MD FACP Instituto Conmemorativo Gorgas de Estudios de la Salud Avenida Justo Arosemena Panamá City, Panama Tel: +507-527-4890 |
| Clinical Laboratory | Laboratorio Central de Referencia en Salud Publica Avenida Justo Arosemena Entre calle 35 y 36 Panamá City, Panama  Tel: +507-527-4848 Laboratorio del Hospital Aquilino Tejeira Vía Panamericana Penonomé, Coclé, Panamá Laboratorio del Centro de Salud Materno Infantil Sandra Hernández Empalme El Silencio, Vía Principal Changuinola, Bocas del Toro, Panamá |
| Parasitology Laboratories | Dr. José Calzada Instituto Conmemorativo Gorgas de Estudios de la Salud Avenida Justo Arosemena Panama City, Panama Entre Calle 35 y 36 Tel: +507-527-4815  Walter Reed Army Institute of Research Silver Spring, MD 20910-7500 Tel: +1 301-319-3763 Email: usarmy.detrick.medcom-wrair.mbx.leishmania-diagnostic@mail.mil |
| Statisticians | Charles Scott, PhD Douglas Tang, PhD Fast-Track Drugs & Biologics, LLC 5 Paramus Court, North Potomac, MD 20878 Tel: +1 215-880-7055 Email: [cscott@fasttrackresearch.com](mailto:cscott@fasttrackresearch.com) |
| Data Management | Janet H. Ransom, PhD Fast-Track Drugs & Biologics, LLC 5 Paramus Court, North Potomac, MD 20878 Tel: +1 301-762-5787 Email: jransom@fasttrackresearch.com |
| Independent Ethics Committee: | Gorgas Institutional Bioethics Committee Instituto Conmemorativo Gorgas de Estudios de la Salud Avenida Justo Arosemana Entre calles 35 y 36 Panama City, Panama Tel: +507-527-4823 or +507-527-4931 Email: combioetica@gorgas.gob.pa |

**FOR OFFICIAL USE ONLY**

Information and data included in this document contain privileged and/or proprietary information, which is the property of the United States Army. No person is authorized to make it public without express written permission of the United States Army. These restrictions on disclosure will apply equally to all future information, which is indicated as privileged or proprietary.

Investigator’s Agreement

A Randomized, Double-blind, Pivotal Phase 3 Study of WR 279,396 (Paromomycin + Gentamicin Topical Cream) and Paromomycin Alone Topical Cream for the Treatment of Cutaneous Leishmaniasis in Panama

“I have read this protocol and agree to conduct the study as outlined herein in accordance with International Conference on Harmonization Good Clinical Practice Guideline and FDA, DoD, and United States Army Regulations.”

Néstor Sosa, MD, FACP Date

Instituto Conmemorativo Gorgas de Estudios de la Salud
Avenida Justo Arosemena
Panama City, Panama

Procedures in Case of Emergency

Table 1: Emergency Contact Information

| Role in Study | Name | Address and Telephone number |
| --- | --- | --- |
| Principal Investigator | Néstor Sosa, MD, FACP | Instituto Conmemorativo Gorgas de Estudios de la Salud Avenida Justo Arosemena Entre Calle 35 y 36 Panama City, Panama Phone : +507-527-4890 Fax: +1 216-71-791-83 E-mail: drnsosa@gmail.com |
| Subinvestigators | José Calzada, PhD Juan Miguel Pascale, MD Ana Jiménez, MD Aracelis Miranda, MSc Zeuz Capitan-Barrios, MSc Vanessa Almengor | Instituto Conmemorativo Gorgas de Estudios de la Salud Avenida Justo Arosemena Entre Calle 35 y 36 Panamá City, Panama Phone : +507-527-4890 |
| Study Coordinators in Panama | Zeuz Capitan-Barrios, MSc Vanessa Almengor | Instituto Conmemorativo Gorgas de Estudios de la Salud Avenida Justo Arosemena Entre Calle 35 y 36 Panamá City, Panama Phone : +507-527-4950 or +507-527-4890 |
| Research Monitor | Maria Eugenia Barnett de Antinori, MD | Regional Training Center Gorgas Memorial Institute Avenida Justo Arosemena Entre Calles 35 y 36 Panamá City, Panama Phone : +507-6615-1338 E-mail: mebantinori@cwpanama.net |
| USAMMDA Clinical Services Support Division (CSSD) | LTC Lela King, PharmD, BCPS | USAMMDA 1430 Veterans Drive Fort Detrick, MD 21702-5009 Phone: +1 301-619-1095 or +1 301-619-9652 Fax: +1 301-619-0197 |
| Sponsor’s Representative | Kenneth A. Bertram, MD, PhD | US Army Medical Research and Materiel Command (USAMRMC) 504 Scott StreetFt. Detrick, MD 21702-5012  301-619-4517-0317; DSN: 343-4517 Fax: 301-619-2982 E-mail: usarmy.detrick.medcom-usammda.mbx.usamrmc-regulatory-affairs@mail.mil |
| USAMMDA Division of Regulated Activity and Compliance | Robert E. Miller, PhD RAC | USAMMDA 1430 Veterans Drive Fort Detrick, MD 21702-5009 Phone : +1 301-619-0317  E-mail: usarmy.detrick.medcom-usammda.mbx.usamrmc-regulatory-affairs@mail.mil |
| Ethics Committee | Gorgas Institutional Bioethics Committee | Gorgas Memorial Institute Avenida Justo Arosemana Entre calles 35 y 36 Phone : +507-527-4823 or +507-527-4931 Email: combioetica@gorgas.gob.pa |

1. Synopsis

| **Name of Sponsor:**  The Surgeon General, Department of the Army | |
| --- | --- |
| **Name of Investigational Products:**  WR 279,396 (Paromomycin and Gentamicin Topical Cream) and Paromomycin Alone Topical Cream | |
| **Name of Active Ingredients:**  Paromomycin sulfate and gentamicin sulfate | |
| **Title of Study:** A Randomized, Double-blind, Pivotal Phase 3 Study of WR 279,396 (Paromomycin + Gentamicin Topical Cream) and Paromomycin Alone Topical Cream for the Treatment of Cutaneous Leishmaniasis in Panama | |
| **Study Centers:** Instituto Conmemorativo Gorgas de Estudios de la Salud, Avenida Justo Arosemena, Panama City, Panama; Hospital Aquilino Tejeira, Penonome, Cocle; and Centro Materno Infantil Sandra Hernandez, El Silencio, Changuinola, Bocas del Toro | |
| **Principal Investigator:** Néstor Sosa, MD, FACP  **Subinvestigators:** José Calzada, PhD, Ana Jiménez, MD, Juan Miguel Pascale, MD, Aracelys Miranda, MSc, Zeuz Capitan- Barrios, MSc, Vanessa Almengor, Franz Barnes, MD, Robert Samuels, MD, ENT, Betsy Ortiz, RN,Azael Saldaña, PhD, Kadir González, Jorge Tuñon, MD, Suzette Chacón, Allin Lewis, Rodileida Castillo, RN, Xochilt Rugmama, RN, Maurice Coronado, MD, Laureano Aguilar, MD, Elvia Valdés, RN, Naty Aguirre, RN, Angélica Pedrouzo, and José Velásquez. | |
| **Study Period (years):**  Estimated date first subject enrolled: May 2013  Estimated date last subject completed: December 2015 | **Phase of development:** 3 |
| **Objectives:**  **Primary Objective:** The primary objective of this study is to determine if WR 279,396 (Paromomycin + Gentamicin Topical Cream) results in statistically superior final clinical cure rates of an index lesion when compared with Paromomycin Alone Topical Cream for the treatment of cutaneous leishmaniasis (CL) in Panama expected to be caused by *Leishmania panamensis (L panamensis)*.  **Secondary Objectives:** Secondary objectives include evaluating other efficacy parameters including: 1) percentage of subjects with all lesions cured; 2) percentage of all lesions cured; 3) lesion sizes over time; 4) cure rates over time, and 5) median time to initial cure to determine if these parameters are superior in the WR 279,396 group compared with the Paromomycin Alone group. An additional secondary objective is to determine the safety of both topical creams in this study population. | |
| **Methodology:** Subjects will be recruited from three regions in Panama known to be endemic for *L panamensis* CL. Subjects will be screened over a period up to 28 days for eligibility including medical history, physical examination, leishmaniasis history, vital signs, clinical chemistry, prior medications, and parasitology for confirmation of ulcerative CL. If eligible, subjects will be randomized in a targeted 1:1 ratio (200 subjects per group) using site as a stratification variable to receive either WR 279,396 (15% paromomycin + 0.5% gentamicin topical cream) or Paromomycin Alone (15% paromomycin topical cream) by topical application to CL lesions once daily for 20 days. Efficacy will be assessed by measuring the size of the index lesion ulcer, non-index lesions ulcers, and overall size of other non-ulcerated lesions at baseline (before the start of treatment), and on Study Days 20, 35 ± 2 days, 49 ± 4 days, 63 ± 7 days, 100 ± 14 days, and 168 ± 14 days. A notation will be made if clinical evidence of parasite persistence is observed at the Day 63 and beyond visits including significant erythema and induration when a lesion has otherwise completely re-epithelialized to document any subjects removed from the study early if the investigator judges them to be in need of rescue treatment. A photograph will be taken of all lesions at baseline, Day 20 and each of the follow-up visits. Safety will be assessed by monitoring adverse events (AEs) from the start of treatment until study completion, lesion site reactions during treatment, physical examination of the nasal and oral mucosa for appearance of mucosal leishmaniasis on Days 63 ± 7 days, 100 ± 14 days, and 168 ± 14 days, concomitant medication use for the duration of the study, blood creatinine, alanine aminotransferase (ALT) and aspartate aminotransferase (AST) levels on Study Day 20. After the sponsor’s approval, biochemistry can be repeated in the case of abnormal results and if the causes of these results could not be determined. A repeat pregnancy test on Day 35. Recent infection with leishmaniasis prior to the start of the study may result in the development of lesions that were not present at the start of the study that did not receive treatment. New lesions may be treated at the discretion of the investigator with the topical cream to which the subject was assigned any time during the conduct of the study except that treatment must be completed by the Day 168 visit. If a new lesion is discovered at the final study visit, the subject will be referred to their primary physician for treatment.  Subjects who fail therapy (see definition of failure below) will be taken off study and may be administered rescue therapy at the discretion of the subject’s personal physician. If the subject met the criteria for therapy failure but was undergoing treatment for new lesions, the subject can continue in the study (by signing a consent addendum) if the investigator decides it is in the best interest of the subject to do so. | |
| **Estimated Number of Subjects Screened:** 600 | |
| **Estimated Number of Subjects Planned and Enrolled:** 400 | |
| **Diagnosis and Criteria for Inclusion/Exclusion:**  **Inclusion Criteria:**   - Male or female at least 2 years-of-age - Subject or legal guardian able to give written informed consent or assent, as appropriate - Diagnosis of CL in at least one lesion by at least one of the following methods: 1) positive culture for promastigotes or 2) microscopic identification of amastigotes in stained lesion tissue - At least one ulcerative lesion ≥ 1 cm and ≤ 5 cm that has a diagnosis of CL - Willing to forego other forms of treatments for CL including other investigational treatments during the study - In the opinion of the investigator, the subject (or their legal guardian), is capable of understanding and complying with the protocol - If female and of child-bearing potential, must have a negative serum pregnancy test during screening and agree to use an acceptable method of birth control during the treatment phase and for 1 week after treatment is completed   **Exclusion Criteria:**   - Lesion due to leishmania that involves the nasal or oral mucosa or any signs of mucosal disease that might be due to *Leishmania* - Only a single lesion on the ear with erosive cartilage - Signs and symptoms of disseminated disease in the opinion of the investigator - More than 10 lesions - Female who is breast-feeding or pregnant - Significant organ abnormality, chronic disease such as diabetes, severe hearing loss, evidence of renal or hepatic dysfunction, or creatinine, aspartate aminotransferase (AST), or alanine aminotransferase (ALT) greater than 15% above the upper limit of normal (ULN) as defined by the clinical laboratory defined normal ranges - Received treatment for leishmaniasis including any medication with pentavalent antimony including sodium stibogluconate (Pentostam™), meglumine antimoniate (Glucantime™); amphotericin B (including liposomal amphotericin B and amphotericin B deoxycholate); or other medications containing paromomycin (administered parenterally or topically) or methylbenzethonium chloride (MBCL); gentamicin; fluconazole; ketoconazole; pentamidine; miltefosine, azithromycin or allopurinol that was completed within 56 days of starting study treatments - After the sponsor’s approval, biochemistry can be repeated in the case of abnormal results and if the causes of these results could not be determined - History of known or suspected hypersensitivity or idiosyncratic reactions to aminoglycosides - Any other topical disease/condition which would interfere with the objectives of this study   NOTE: The index lesion size for study inclusion is maximum diameter of the ulceration and surrounding induration. | |
| **Investigational Products, Dosage and Mode of Administration:** The investigational creams will be applied topically to all lesions once daily through Day 20. Application of investigational cream daily to Day 20 will be continued even if the lesion has obtained 100% re-epithelialization prior to Day 20. Prior to the first application, the lesions will be cleaned and debrided and then dried. At each subsequent application of the investigational cream, the previous day’s application and dressing will be removed, the lesion will be cleaned with soap, water, and sterile 0.9% saline, then dried using sterile cotton gauze sponges, and re-dressed. Lesions will be left undisturbed until the next application. The investigational cream will be applied with a gloved finger to cover the area of the ulcer or raised area of non-ulcerated lesions and rubbed into the lesion. The weight of the tube(s) containing the investigational cream will be measured through the use of an analytical balance prior to the start of treatment and after the completion of treatment on Day 20. The difference in weight will indicate how much investigational cream was applied. Each site will possess an analytical balance. If new lesions appear, the tubes will be weighed one final time after completion of treatment of all new lesions. | |
| **Duration of Treatment:** 20 days | |
| **Criteria for Evaluation:**  **Primary Efficacy Endpoint:**  Percentage of subjects with final clinical cure. **Final clinical cure** is defined as follows:   - Subject has initial clinical cure (100% re-epithelialization of index lesion by nominal Day 63); OR, - Subject has initial clinical improvement (> 50% re-epithelialization of index lesion by nominal Day 63) followed by 100% re-epithelialization of the index lesion on or before nominal Day 100; AND, - Subject has no relapse of index lesion.   **Relapse** is defined as an index lesion meeting the criteria for initial clinical cure that had any new ulceration (> 0 x 0 mm measurement) by nominal day 168, or an index lesion meeting the criteria for initial clinical improvement that subsequently enlarged by nominal Day 168.  **Failure** is the opposite of final clinical cure: Subjects who drop out of the study early (ie, are not assessed at the final Day 168 visit) will also be considered clinical failures.  **Secondary Efficacy Endpoints:**   - Percentage of subjects with all lesions cured, defined as:  1. Final clinical cure as defined above (which is based solely on the index lesion); AND, 2. Cure of all other lesions by nominal Day 100 (100% re-epithelialization of all ulcerated lesions).  - Percentage of all lesions cured at Day 168 - Area of ulceration of the index lesion at each measurement time point - Area of ulceration for all treated lesions at each measurement time point - Ulcerated lesion complete cure rate at each measurement time point (complete cure is defined as 100% re-epithelialization of an ulcerated lesion) - Median time to initial clinical cure (100% re-epithelialization of the index lesion)   **Safety Endpoints Include:**   - AEs including application site reactions including elicited examination for pain, and clinician examination for erythema/redness, swelling/edema, and vesicles - Blood creatinine, AST, and ALT | |
| **Statistical Methods:**  Descriptive statistics will be used to present study data. Continuous variables will be presented as number of observations (n), mean, standard deviation (SD), median, minimum, and maximum values. Categorical variables will be presented as counts and percentages.  **Analysis Groups:** Modified intention-to-treat (mITT) and safety analysis sets: The mITT and safety analysis sets include all subjects who received any administration of investigational product. The evaluable subset will include all subjects who received daily doses of investigational product for at least 18 of the total 20 days and had lesion measurements at Day 63 and 168. Efficacy will also be presented by subsets of subjects presenting with different species of *Leishmania*.  **Demographics and Treatment Compliance.** Summaries of the subject demographics (age, gender, ethnicity and race), number of baseline lesions, lesion sizes, infecting *Leishmania* species and length of time between initial presence of current lesions and treatment) will be provided. Compliance with scheduled treatments will also be summarized, including total exposure (number of days and total dose) to study drug. Protocol compliance will be presented as percentages of subjects attending scheduled visits over the duration of the study.  **Safety Data.** AEs will be coded using the most recent version of the Medical Dictionary of Regulatory Activities (MedDRA) preferred terms and will be grouped by system, organ, and class (SOC) designation. The severity, frequency, and relationship of AEs to study drug will be presented by preferred term by SOC grouping. Laboratory data will be presented as summary statistics by study day including changes from baseline and in by-subject data listings. Percentages of values above the normal laboratory limits at Day 20 will be presented.  **Efficacy Data.** The primary endpoint of the percentage of subjects who meet the criteria for final clinical cure of the index lesions and the secondary endpoints of percentage of subjects with all lesions cured, and percentage of all lesions cured without regard to subject will be presented for the mITT population and the evaluable population by treatment group. These endpoints will be compared between the two treatment groups by two-sided uncorrected chi-square test. P-values < .05 will be considered statistically significant. Subjects with missing data making it impossible to determine if lesions have cured will be considered failures in these analyses. Summary statistics for lesion area measurements including area of ulceration of each subject’s index lesion and total area of ulceration of all lesions by subject will be presented both in tabular form and graphically. Areas over time will be compared between the two treatment groups by mixed effects model using an appropriate transformation to normalize the data. The percentages of subjects with 100% re-epithelialization of the index lesion and all ulcerated lesions over time will be compared between treatment groups using a mixed effects model with treatment and time in the model. If data are not normally distributed appropriate transformations will be performed. The number of new lesions (if any) that develop during the study and their response to investigational products will also be presented. The proportions of subjects with final clinical cure of the index lesion and all lesions by *Leishmania* species will be presented by treatment group. Median time to initial clinical cure will be determined using time to events methods. Time to initial clinical cure will be compared between the two groups using log-rank test. | |

1. Table of Contents, List of Tables, and List of Figures

**Table of Contents**

[1. Title Page 1](#_Toc366065341)

[2. Synopsis 6](#_Toc366065342)

[3. Table of Contents, List of Tables, and List of Figures 11](#_Toc366065343)

[4. List of Abbreviations and Definitions of Terms 15](#_Toc366065344)

[5. Introduction 17](#_Toc366065345)

[Cutaneous Leishmaniasis Worldwide 17](#_Toc366065346)

[Cutaneous Leishmaniasis in the New World and in Panama 17](#_Toc366065347)

[Treatment of CL 20](#_Toc366065348)

[Military Relevance 21](#_Toc366065349)

[Rationale for Study 21](#_Toc366065350)

[5.1. Name and Description of the Investigational Product 21](#_Toc366065351)

[5.2. Summary of Nonclinical and Clinical Trials 22](#_Toc366065352)

[5.2.1. Mechanism of Action 22](#_Toc366065353)

[5.2.2. Nonclinical Toxicology Studies of WR 279,396 22](#_Toc366065354)

[5.2.3. Nonclinical Efficacy Studies of Paromomycin, Gentamicin, and WR 279,396 22](#_Toc366065355)

[5.2.4. Clinical Studies 24](#_Toc366065356)

[5.2.4.1. Efficacy 24](#_Toc366065357)

[5.2.4.2. Safety 25](#_Toc366065358)

[5.2.4.3. Pharmacokinetics 26](#_Toc366065359)

[5.3. Known and Potential Risks and Benefits to Human Subjects 27](#_Toc366065360)

[5.3.1. Risks/Discomfort to Subjects and Precautions to Minimize Risk 27](#_Toc366065361)

[5.3.1.1. Skin/Local Reactions 27](#_Toc366065362)

[5.3.1.2. Allergic Reactions to the Dressing 27](#_Toc366065363)

[5.3.1.3. Systemic Reactions: Hearing and Kidneys 28](#_Toc366065364)

[5.3.1.4. Pregnancy 28](#_Toc366065365)

[5.3.1.5. Breast Feeding 28](#_Toc366065366)

[5.3.1.6. Unknown Risks 28](#_Toc366065367)

[5.3.2. Research-Related Injuries 28](#_Toc366065368)

[5.3.3. Alternatives to this IND Product or Study 29](#_Toc366065369)

[5.3.4. Intended Benefit for Subjects 29](#_Toc366065370)

[5.4. Route of Administration, Dosage Regimen, Treatment Period, and Justification 29](#_Toc366065371)

[5.5. Compliance Statement 29](#_Toc366065372)

[5.6. Study Population 29](#_Toc366065373)

[5.7. Study Sites 30](#_Toc366065374)

[6. Trial Objectives and Purpose 31](#_Toc366065375)

[6.1. Primary Objectives 31](#_Toc366065376)

[6.2. Secondary Objectives 31](#_Toc366065377)

[7. Trial Design 32](#_Toc366065378)

[7.1. Study Endpoints 32](#_Toc366065379)

[7.1.1. Primary Efficacy Endpoint 32](#_Toc366065380)

[7.1.2. Secondary Efficacy Endpoints 33](#_Toc366065381)

[7.1.3. Safety Endpoints 33](#_Toc366065382)

[7.2. Overall Study Design 33](#_Toc366065383)

[7.3. Measures Taken to Minimize/Avoid Bias 33](#_Toc366065384)

[7.3.1. Blinding 35](#_Toc366065385)

[7.4. Investigational Product 35](#_Toc366065386)

[7.4.1. Description 35](#_Toc366065387)

[7.4.2. Investigational Product Packaging and Labeling 35](#_Toc366065388)

[7.4.3. Investigational Product Storage 36](#_Toc366065389)

[7.5. Duration of Subject Participation 36](#_Toc366065390)

[7.6. Dose-adjustment Criteria 36](#_Toc366065391)

[7.6.1. Safety Criteria for Dose Adjustment or Stopping Doses 36](#_Toc366065392)

[7.6.2. Study Termination 36](#_Toc366065393)

[7.7. Investigational Product Accountability 36](#_Toc366065394)

[7.8. Trial Treatment Randomization Codes 37](#_Toc366065395)

[7.9. Identification of Data to be Recorded on the Case Report Forms 37](#_Toc366065396)

[8. Selection and Withdrawal of Subjects 38](#_Toc366065397)

[Recruitment of Subjects 38](#_Toc366065398)

[Informed Consent Process 38](#_Toc366065399)

[8.1. Eligibility Screening 38](#_Toc366065400)

[8.1.1. Demographics 38](#_Toc366065401)

[8.1.2. Medical History 39](#_Toc366065402)

[8.1.3. Physical Exam 39](#_Toc366065403)

[8.1.4. Pregnancy Test 39](#_Toc366065404)

[8.1.5. Vital Signs 39](#_Toc366065405)

[8.1.6. Blood for Chemistry and Pregnancy Tests 39](#_Toc366065406)

[8.1.7. Leishmaniasis History 39](#_Toc366065407)

[8.1.8. Diagnosis of Leishmaniasis 40](#_Toc366065408)

[8.2. Eligibility Checklist 40](#_Toc366065409)

[8.3. Subject Inclusion Criteria 40](#_Toc366065410)

[8.4. Subject Exclusion Criteria 41](#_Toc366065411)

[8.5. Subject Withdrawal Criteria 41](#_Toc366065412)

[8.5.1. When and How to Withdraw Subjects 42](#_Toc366065413)

[8.5.2. Data Collected for Withdrawn Subjects 42](#_Toc366065414)

[8.5.3. Replacement of Subjects 42](#_Toc366065415)

[8.5.4. Follow-up for Withdrawn Subjects 43](#_Toc366065416)

[9. Treatment of Subjects 44](#_Toc366065417)

[9.1. Baseline Assessments Prior to Application of the Study Drugs 44](#_Toc366065418)

[9.1.1. Identification of the Index Lesion 44](#_Toc366065419)

[9.1.2. Lesion Photographs 45](#_Toc366065420)

[9.2. Application of the Study Drug 45](#_Toc366065421)

[9.3. Instructions for Subjects 46](#_Toc366065422)

[9.4. Concomitant Medications 46](#_Toc366065423)

[9.5. Procedures for Monitoring Subject Compliance 46](#_Toc366065424)

[9.6. Study Days 2 through 19 47](#_Toc366065425)

[9.7. Study Day 20 47](#_Toc366065426)

[9.8. Study Days 35 ± 2 days and 49 ±4 days 47](#_Toc366065427)

[9.9. Study Days 63 ±7 days, 100 ± 14 days and 168 ± 14 days 47](#_Toc366065428)

[10. Efficacy Assessments 49](#_Toc366065429)

[10.1. Specification of Efficacy Endpoints 49](#_Toc366065430)

[10.2. Methods/Timing for Assessing, Recording, and Analyzing Efficacy Endpoints 49](#_Toc366065431)

[11. Safety Assessments 50](#_Toc366065432)

[11.1. Specification of Safety Endpoints 50](#_Toc366065433)

[11.2. IND Safety Reporting 50](#_Toc366065434)

[11.2.1. Adverse Event or Suspected Adverse Reaction 51](#_Toc366065435)

[11.2.2. Serious Adverse Event or Serious Suspected Adverse Drug Reaction 51](#_Toc366065436)

[11.2.3. Unexpected Adverse Event or Unexpected Suspected Adverse Reaction 52](#_Toc366065437)

[11.3. Relationship to Investigational Product 52](#_Toc366065438)

[11.4. Recording Adverse Events 53](#_Toc366065439)

[11.4.1. Methods/Timing for Assessing, Recording, and Analyzing Safety Endpoints 53](#_Toc366065440)

[11.4.2. Duration of Follow-Up of Subjects after Adverse Events 53](#_Toc366065441)

[11.4.3. Severity Assessment 53](#_Toc366065442)

[11.4.4. AE Actions and Outcomes 54](#_Toc366065443)

[11.5. Reporting Adverse Events 54](#_Toc366065444)

[11.5.1. Reporting Serious and Unexpected Adverse Events 54](#_Toc366065445)

[11.5.1.1. Reporting to the Sponsor 54](#_Toc366065446)

[11.5.1.2. Reporting to the Ethics Committee 57](#_Toc366065447)

[11.5.1.3. Reporting to ORP HRPO 57](#_Toc366065448)

[11.5.2. Reporting Additional Immediately Reportable Events to the Sponsor’s Representative and ORP HRPO 57](#_Toc366065449)

[11.5.2.1. Pregnancy 57](#_Toc366065450)

[11.5.2.2. AE-related Withdrawal of Consent 58](#_Toc366065451)

[11.5.2.3. Pending Inspections/Issuance of Reports 58](#_Toc366065452)

[11.5.3. IND Annual Report to the FDA 58](#_Toc366065453)

[11.5.4. Final Report 58](#_Toc366065454)

[12. Statistics 59](#_Toc366065455)

[12.1. Description of Statistical Methods 59](#_Toc366065456)

[12.1.1. Analysis Addressing the Primary Study Efficacy Objective 59](#_Toc366065457)

[12.1.2. Analysis Addressing the Secondary Study Objectives 59](#_Toc366065458)

[12.1.2.1. Percentage of Subjects with All Lesions Cured 59](#_Toc366065459)

[12.1.2.2. Percentage of All Lesions Cured 59](#_Toc366065460)

[12.1.2.3. Lesion Area Measurements 59](#_Toc366065461)

[12.1.2.4. Ulcerated Lesion Cure Rate 60](#_Toc366065462)

[12.1.2.5. Time to Initial Clinical Cure 60](#_Toc366065463)

[12.1.2.6. New Lesions Incidence 60](#_Toc366065464)

[12.1.3. Safety Analyses 60](#_Toc366065465)

[12.1.3.1. Demographics and Treatment Compliance 60](#_Toc366065466)

[12.2. Planned Enrollment and Reason for Sample Size 61](#_Toc366065467)

[12.3. Interim Analysis 62](#_Toc366065468)

[12.4. Accounting for Missing, Unused, and Spurious Data 62](#_Toc366065469)

[12.5. Procedures for Reporting Deviations from the Original Statistical Plan 62](#_Toc366065470)

[12.6. Selection of Subjects to be Included in Analyses 62](#_Toc366065471)

[13. Direct Access to Source Data/Documents 63](#_Toc366065472)

[13.1. Study Monitoring 63](#_Toc366065473)

[13.2. Audits and Inspections 63](#_Toc366065474)

[13.3. Ethics Committee 63](#_Toc366065475)

[14. Quality Control and Quality Assurance 65](#_Toc366065476)

[15. Ethics 66](#_Toc366065477)

[15.1. Ethics Review 66](#_Toc366065478)

[15.1.1. Review/Approval of Study Protocol 66](#_Toc366065479)

[15.1.2. Protocol Modifications 66](#_Toc366065480)

[15.1.3. Protocol Deviation Procedures 67](#_Toc366065481)

[15.2. Ethical Conduct of the Study 67](#_Toc366065482)

[15.2.1. Confidentiality 67](#_Toc366065483)

[15.2.2. Compensation for Participation 68](#_Toc366065484)

[15.3. Written Informed Consent 68](#_Toc366065485)

[16. Data Handling and Recordkeeping 70](#_Toc366065486)

[16.1. Inspection of Records 70](#_Toc366065487)

[16.2. Retention of Records 70](#_Toc366065488)

[17. Publication Policy 72](#_Toc366065489)

[18. List of References 73](#_Toc366065490)

[Appendix A. Study Personnel Roles and Responsibilities 77](#_Toc366065491)

[Appendix B. Body Chart of Lesion Sites 79](#_Toc366065492)

**List of Tables**

[Table 1: Emergency Contact Information 5](#_Toc366061575)

[Table 2: Abbreviations 15](#_Toc366061576)

[Table 3: Incidence of New World Cutaneous Leishmaniasis, By-Country 18](#_Toc366061577)

[Table 4: Time Frame for Self-Healing of CL Lesions in Various Species 19](#_Toc366061578)

[Table 5: Efficacy of WR 279,396 (WR) Compared with Paromomycin-MBCL-Paraffin (PM) in *L major*-, *L mexicana*-, *L panamensis*-, and *L amazonensis*‑infected BALB/c Mice 23](#_Toc366061579)

[Table 6: Primary and Secondary Efficacy Endpoints in Phase 2 Panama Study 25](#_Toc366061580)

[Table 7: Summary of Paromomycin Pharmacokinetics Following Administration of WR 279,396 Adults on Day 1 and Day 20 26](#_Toc366061581)

[Table 8: Predicted Percentage of the Topical Dose of Paromomycin Absorbed and Paromomycin Accumulation Following Administration of WR 279,396 to Adult Subjects 27](#_Toc366061582)

[Table 9: Schedule of Procedures 34](#_Toc366061583)

[Table 10: Study Contacts for Reporting Serious Adverse Events 56](#_Toc366061584)

[Table 11: SAE Information to be Reported to the Sponsor's Representative 57](#_Toc366061585)

[Table 12: Sample Size Estimates 61](#_Toc366061586)

**List of Figures**

[Figure 1: Decision Tree for Selection of the Index Lesion 45](#_Toc366061587)

1. List of Abbreviations and Definitions of Terms

The following abbreviations are used in this study protocol.

Table 2: Abbreviations

| Abbreviation | Definition |
| --- | --- |
| μM | Micromolar |
| AE | Adverse event |
| ALT | Alanine aminotransferase |
| AST | Aspartate aminotransferase |
| AUC | Area under the plasma concentration time curve |
| CI | Confidence interval |
| CL | Cutaneous leishmaniasis |
| C_max_ | Maximum plasma concentration |
| CRF | Case report form |
| CSSD | Clinical Services Support Division |
| DoD | Department of Defense |
| ED_50_ | Estimated dose of 50% lethality |
| FDA | Food and Drug Administration |
| GCP | Good clinical practices |
| h | Hours |
| ICH | International conference on harmonization |
| ID | Identification |
| IND | Investigational New Drug |
| IV | Intravenous(ly) |
| *L amazonensis* | *Leishmania amazonensis* |
| *L braziliensis* | *Leishmania braziliensis* |
| *L donovani* | *Leishmania donovani* |
| *L major* | *Leishmania major* |
| *L mexicana* | *Leishmania mexicana* |
| *L panamensis* | *Leishmania panamensis* |
| MBCL | Methylbenzethonium chloride |
| MedDRA | Medical Dictionary for Regulatory Activities |
| mITT | Modified Intention-to-treat |
| mL | Milliliter |
| mm | Millimeter |
| ORP/HRPO | USAMRMC Office of Research Protections/Human Research Protection Office |
| PCR | Polymerase Chain Reaction |
| SAE | Serious adverse event |

Table 2: Abbreviations (Continued)

| Abbreviation | Definition |
| --- | --- |
| SAS | Statistical Analysis System |
| SD | Standard Deviation |
| SSP | Study Specific Procedure |
| t_1/2_ | Apparent terminal exponential half-life |
| T_max_ | Time of maximum plasma concentration |
| ULN | Upper limit of normal |
| USAMMDA | US Army Medical Materiel Development Activity |
| USAMRMC | US Army Medical Research and Materiel Command |
| w/w | Weight of drug to weight of other drug components expressed as a percentage |

1. Introduction

Cutaneous Leishmaniasis Worldwide

Leishmaniasis, an illness caused by *Leishmania* spp protozoa, is transmitted by infected female phlebotomine sandflies. It occurs in approximately 98 tropical and subtropical countries around the world, including portions of both the New World (tropical and subtropical areas of the Americas) and the Old World (primarily southwestern Asia, Africa, and southern Europe) [World Health Organization ([WHO-2014](#UnderSecretaryforHealth2004))]. In the Old World (the Mediterranean, the Middle East and Southwest Asia, the Indian subcontinent, Central Asia), most lesions are papules, nodules, or nodule-ulcers. In the New World (from the Texas-Mexico border down through South America to the level of the Tropic of Capricorn), ulcerative lesions are most common. The estimated annual number of new cases of cutaneous leishmaniasis (CL) is about 1.3 million ([WHO-2014](#WHO2014)).

Cutaneous Leishmaniasis in the New World and in Panama

The incubation period for CL typically ranges from 1 to 12 weeks, although some cases may take several months to manifest ([Hepburn-2003](#Hepburn2003); [Gontijo & de Carvalho-2003](#Gontijo2003)). The illness has a variety of skin manifestations, including the following: small, dry, crusted lesions; ulcerative lesions that are shallow and circular with well-defined borders and a bed of granulation tissue; and large, deep, mutilating ulcers ([Gontijo & de Carvalho-2003](#Gontijo2003)). In many cases, the disease begins as an erythematous papule at the site of the sand fly bite on an exposed portion of the body. The papule increases in size, becomes a nodule, ulcerates, and eventually crusts over. The resulting lesion is a crusted ulcer with a necrotic base, which is surrounded by a raised, indurated margin ([Hepburn-2003](#Hepburn2003)). The mean area per ulcer in one study was 400 mm^2^, but, in rare cases, an ulcer may measure 2,000 mm^2^ ([Soto-Mancipe et al-1993](#SotoMancipe1993)). Most patients with CL have only one or two lesions, ranging from 0.5 to 3.0 cm in diameter; however, some have multiple sites of infection resulting in as many as 20 CL lesions (often due to encountering a nest of sand flies) ([Hepburn-2003](#Hepburn2003)). Although the typical CL ulcer may be large, it is usually painless, unless there is a secondary bacterial or fungal infection ([Hepburn-2003](#Hepburn2003)).

With respect to New World disease, Brazil ranks among the top 8 nations of the world in terms of CL incidence, with an estimated 35,000 new cases annually ([Seimenis et al-2006](#Seimenis2006)). A further 20,000 cases are reported each year in the Central American nations, with approximately 30,000 more in the Andean countries (Table 3). These incidence figures probably represent underestimates, since the incidence of CL in many New World nations is increasing ([Carreira et al-1995](#Carreira1995); [Christensen and Petersen-1999](#Christensen1999); [Saenz-Anduaga and Chavez-Mancilla-2004](#SaenzAnduaga2004)).

In Panama, leishmaniasis is an important parasitic disease with recent numbers of new cases of CL approximating 3,000 per year. The rates of reported infections currently vary in the range of 60-110 per 100,000 individuals ([República de Panamá-2000-2007](#Republica2000)). These rates are a low estimate by as much as 50% since there is likely to be significant under-reporting due to the fact that there are many cases occurring in remote, rural locations with few if any medical facilities available for diagnosis and treatment ([Vásquez et al-1998](#Vásquez1998)). Records from the Epidemiology Department of the Ministry of Health of Panama indicate that there was a gradual decrease in the number of reported cases from year 2000 (2,426 cases) to year 2005 (1,649 cases). In 2006, that number jumped to 3,774 cases followed by a decrease to 2,069 cases in 2007([República de Panamá-2000-2007](#Republica2000)). Since data are only available since 2000, it is not yet possible to tell if there are regular cycles in the numbers of cases over several years or if there is an upward trend currently developing.

Among the cases reported, approximately 95% of them were diagnosed as *L panamensis* with small numbers of *Leishmania braziliensis* accounting for the balance. On rare occasions, simultaneous infections by two species have been reported ([Calzada-Not Dated](#Calzada)). *L panamensis* is typically limited to producing only CL lesions. However, it does have the potential to progress to mucocutaneous leishmaniasis in approximately 5% of cases ([Miranda et al-2009](#Miranda2009)).

Table 3: Incidence of New World Cutaneous Leishmaniasis, By-Country

| Country | Annual Number of CL Cases | Last Year Reported | Reference |
| --- | --- | --- | --- |
| Brazil | 35,000 28,575 | 1999 2004 | [Gontijo and Carvalho-2003](#Gontijo2003)  [OPS-2007](#Organizacion2007) |
| Panama | 3,774 2069 | 2006 2007 | República de Panamá |
| Colombia | 20,000 22,000 | 1998 2004 | [Davies et al-2000](#Davies2000)  [OPS-2007](#Organizacion2007) |
| Peru | 2,668 3,153 | 1998 1999 | [Davies et al-2000](#Davies2000)  [OPS-2007](#Organizacion2007) |
| Peru | 2,240 2,800 | 1998 2004 | [Davies et al-2000](#Davies2000)  [OPS-2007](#Organizacion2007) |
| Bolivia | 1,936 | 1998 | [Davies et al-2000](#Davies2000)  [OPS-2007](#Organizacion2007) |
| Ecuador | 1,936 | 1998 | [Davies et al-2000](#Davies2000) |
| Ecuador | 1,133 | 2003 | [OPS-2007](#Organizacion2007) [Davies et al-2000](#Davies2000) |
| Paraguay | 348 | 2004 | [OPS-2007](#Organizacion2007) |
| French Guiana | 348 | 2004 | [Rotureau et al-2007](#Rotreau2006) |

a Organización Panamericana de la Salud (a regional office of the World Health Organization).

Spontaneous healing may occur in patients with CL infections, with the rate of healing influenced by many factors, including the particular infecting species of *Leishmania* parasite. Although various estimates of the time frame required for the self-healing of CL lesions are not entirely consistent (Table 4), there is some general agreement regarding the fact that both *Leishmania major (L major)* and *L mexicana* have the greatest tendency to self-heal within the most rapid time frame (typically 3-9 months). When CL lesions heal spontaneously, there often remains a depressed scar that is round or (occasionally) irregular. The propensity for untreated CL to result in substantial scarring is well known among patients in endemic areas ([Asilian et al‑2003](#Asilian2003)).

Table 4: Time Frame for Self-Healing of CL Lesions in Various Species

| Reference | *L major* | *L tropica* | *L mexicana* | *L braziliensis* | *L panamensis* |
| --- | --- | --- | --- | --- | --- |
| [Asilian et al-1995](#Asilian1995)  [Zakraoui et al-1995](#Zakraoui1995) | 15%-45% of lesions heal within 6-8 months | 15%-45% of lesions heal within 6-8 months |  |  |  |
| [Hepburn-2003](#Hepburn2003) | 50% of lesions heal within 3 months | 50% of lesions heal within 10 months | 50% of lesions heal within 3 months | Lesions generally persist more than 10 months |  |
| [Reithinger et al-2007](#Reithinger2007) | Self-cure occurs at 3 months | Self-cure occurs at 6-15 months | Self-cure occurs at 3-9 months | Self-cure occurs at 6-15 months | Self-cure occurs at 6-15 months |
| [Murray et al-2005](#Murray2005) | Self-healing at 2-4 months |  | Self-healing at 3 months | Only 10% heal within 3 months | 35% heal within 3 months |
| [Andrade-Narvaez et al-2001](#Andrade2001) |  |  |  | 6% healed within 14 weeks |  |
| [Christensen et al-1999](#Christensen1999) |  |  |  |  | 6% healed within 14 weeks, but “a few lesions” persisted more than 10 years |
| [Lesho et al-2005](#Lesho2005) | Average time to self-healing is 5 months | Time to self-healing is approximately 1 year | Average time to self-healing is 8 months | Time to self-healing is approximately 1 year |  |

a Authors cited work by [Herwald & Berman (1992)](#Herwaldt1992).

The frequency of secondary bacterial infections in CL lesions varies by-country, possibly influenced by species-related differences in the underlying *Leishmania* infection. Rates of secondary infections have been reported as follows: 35.7% in Iran; 54.2% - 67.7% in Brazil; 90.9% in Mexico; and Peru 30% ([Edrissian et al-1990](#Edrissian1990); [Fontes et al-2005](#Fontes2005); [Isaac-Marquez et al‑2003](#IsaacMarquez2003)).

Iranian researchers have documented the presence of coagulase-positive staphylococci (the predominant species) in CL ulcers, as well as that of *Streptococcus* species, *Pseudomonas* species, *Klebsiella* species and *E coli* ([Edrissian et al-1990](#Edrissian1990))*.* In the New World (Brazil), Fontes et al (2005) similarly found that Staphylococcus was the predominant species in CL lesions, followed by *Proteus mirabilis, Streptococcus pyogenes, Proteus species, Klebsiella oxytoca, Enterobacter* species, *Peptostreptococcus* species, *Pseudomonas* species, *Prevotella* *bivia*, *E coli, Streptococcus* *agalactiae,* and *Bacteriodes fragilis.* In a second New World Study (Mexico), bacteria cultured from chiclero’s ulcer (CL due to *L mexicana*) included “*Staphylococcus aureus* (20%), *Staphylococcus pyogenes* (1.6%), *Pseudomonas aeruginosa* (1.6%), *Morganella morganii* (1.6%), and opportunist pathogenic bacteria such as *Klebsiella* spp. (20.0%), *Enterobacter* spp. (20%), and *Enterococcus* spp. (20%)” ([Isaac-Marquez et al-2003](#IsaacMarquez2003)). The fungi, *Candida albicans* and *Candida tropicalis*, were also isolated from CL lesions of Brazilian patients ([Fontes et al-2005](#Fontes2005)).

Few data are available with respect to the impact of secondary bacterial infections on the healing of CL lesions. In the Brazilian study described above, there was some evidence that the presence of bacteria, especially *Proteus* species, retarded the healing process in CL ulcers ([Fontes et al-2005](#Fontes2005)). Also, in the 2003 Mexican study, the authors observed that “individuals with purulent secretion and concomitant infections with *Streptococcus pyogenes, Staphylococcus aureus, Pseudomonas aeruginosa, Morganella morganii,* and *E durans* took longer to heal…when they were treated with antimonial drugs.” ([Isaac-Marquez et al-2003](#IsaacMarquez2003)).

Even after an ulcerated lesion has completely healed, there have been reported cases of relapse, not only in spontaneously cured lesions but also in patients who have been treated with a variety of drug therapies. This phenomenon has also been described in animals. The occurrence of CL relapse results from the persistence of live parasites in clinically “cured” CL lesions ([Ramírez & Guevara-1997](#Ramírez1997)). Even in healed CL lesions that have not clinically relapsed, evidence from biopsied scar tissue has confirmed that live *Leishmania* parasites (*L braziliensis*) may persist for many years and retain their infectivity ([Schubach et al-1998](#Schubach1998)). In addition, as long as 8 years after clinical cure, up to 80% of patients’ CL scars may remain positive by polymerase chain reaction (PCR) methods of detection ([Schubach et al-1998](#Schubach1998)). This tendency to persist following clinical resolution of disease is considered to be a “characteristic of all *Leishmania* species,” not only *L braziliensis* ([Netto et al-1990](#Netto1990)). Persistence after treatment of a few amastigotes in the skin in the absence of a lesion may be beneficial in the long run and the reason why in endemic areas individuals are generally protected after their first infection if infected with the same parasite strain.

In two studies which investigated relapse rates in patients with CL caused by *L braziliensis*, treatment with Glucantime was followed by relapse in approximately 10% of cases, with the majority of relapses (94%) occurring within the first year after therapy ([Ramírez and Guevara‑1997](#Ramírez1997); [Netto et al-1990](#Netto1990)). In a third study involving treatment with injectable paromomycin in Colombian patients, of the 8 of 89 patients that were initially cured, relapses occurred in all of these patients between 3 and 12 months after the end of antileishmanial therapy ([Soto et al-1994](#Soto1994)).

Treatment of CL

Standard treatment for CL is intravenous (IV) or intramuscular (IM) injections of pentavalent antimonials (such as Pentostam or Glucantime). The adverse effects of pentavalent antimonials can be significant and include pancreatitis, hepatitis, marrow suppression, and changes to the electrocardiograph (QT prolongation) ([Herwaldt and Berman-1992](#Herwaldt1992); [Berman-1988](#Berman1988); [Choi and Lerner-2001](#Choi2001)). Other common adverse effects include myalgias, fatigue, headache, rash and nausea. In most instances, these events resolve when therapy is discontinued. Although antimonial regimens have reportedly achieved cure rates of ~90% in some CL studies, lower cure rates of ~50% have also been documented ([Arevalo et al-2007](#Arevalo2007); [Navin et al-1992](#Navin1992); [Bellazoug and Neal-1986](#Bellazoug1986); [Rodrigues et al- 2006](#Rodrigues2006); [Romero et al-2001](#Romero2001); [Saldanha et al-1999](#Saldanha1999)). Recently, a review by [Lesho et al (2005)](#Lesho2005) estimated a 45%-100% efficacy range for parenteral pentavalent antimonials, depending on the infecting *Leishmania* species. In the New World, mounting *Leishmania* resistance to pentavalent antimonials has also been documented, as evidenced by a 612% increase in the total recommended dosage for these drugs in Colombia during the period 1975 to 2006 ([Soto and Soto-2006](#Soto2006)).

The attempt to find alternatives to an antimonial regimen has included oral agents, such as miltefosine, ketoconazole, fluconazole and allopurinol, and short courses of antimonials and pentamidine. The efficacy of these agents have been limited with <75% patients cured ([Alrajhi et al-2002](#Alrajhi2002)).

Several topical agents have been investigated to treat CL and at least one is available commercially outside of the United States. The primary active drug in these topical formulations is paromomycin, an aminoglycoside antibiotic that has an analogous structure to the antibacterial agent neomycin. Although neomycin has only antibacterial activity, paromomycin has activity against protozoa with an effective dose (ED)_100_ of ~10 µg/mL against *Leishmania* amastigotes.

Military Relevance

The large number of recent “operational” cases is due to ongoing military operations in Southwest Asia. CL was the most prominent infectious disease threat seen in Operation Iraqi Freedom, and it persists as a threat ([Armed Forces Health Surveillance Center-2011](#ArmedForces2011)). Between March 2003 and June 2005, an estimated 0.23% of deployed US ground forces participating in Operation Iraqi Freedom received a diagnosis of leishmaniasis ([Aronson-2006](#Aronson2006)), and at least 1,700 cases of leishmaniasis have been diagnosed in US military personnel since January 2003 ([Armed Forces Health Surveillance Center-2011](#ArmedForces2011)). As of 10 December 2004 (the most recent date for which this information has been published), military expenditures for the treatment of leishmaniasis had already approached $22 million ([Byrnes-2004](#Byrnes2004)).

Rationale for Study

The results of Phase 2 and 3 clinical studies conducted to date on WR 279,396 support further investigation of the efficacy and safety of WR 279,396 to treat CL. In a recently completed Phase 3 clinical trial of *L major* CL in Tunisia, WR 279,396 and Paromomycin Alone resulted in significantly higher clinical cure rates compared with Vehicle Control (p < .001, two-sided chi‑square test), although the final clinical cure rates in both the active treatment groups were essentially the same (WR 279,396, 80.8%; Paromomycin Alone , 81.6%; Vehicle 58.4%). In a recent Phase 2 study in Panama, WR 279,396 resulted in higher cure rates than the group treated with Paromomycin Alone (86.7% (13/15) versus 60.0% (9/15), respectively. The final clinical cure rate for the index lesion was not statistically significant when comparing these two treatment groups in this small study designed primarily to collect pharmacokinetic data (chi‑square test p = .099). However, the data from this study as well as in animal models of CL suggest that the gentamicin component in the combination product may improve efficacy outcomes in New World CL. As WR 279,396 is a combination of two aminoglycosides, the superiority of the combination over that of Paromomycin Alone needs to be shown in a larger pivotal trial for product regulatory approval.

- 1. Name and Description of the Investigational Product

WR 279,396 is a topical cream of paromomycin 15% (w/w) that also contains a second aminoglycoside, gentamicin 0.5% (w/w), in a hydrophilic base containing 6.75% urea. WR 279,396 is being developed for the treatment of uncomplicated CL. The current formulation under investigation has been manufactured by Teva Pharmaceuticals USA. A previous similar formulation was manufactured by the University of Iowa that was used in some of the non‑clinical and clinical trials to date.

- 1. Summary of Nonclinical and Clinical Trials
     1. Mechanism of Action

It is believed that the pharmacological activity of paromomycin and gentamicin, like that of the other aminoglycosides, is related to their ability to bind to 30S ribosomal ribonucleic acid (rRNA) at the aminoacyl-tRNA site, thereby causing misreading of the genetic code, inhibition of translation, and the resultant blockade of protein synthesis in target organisms ([Pearson-2000](#Pearson2000)). It has been hypothesized that the gentamicin component of WR 279,396 acts synergistically with paromomycin. Although the precise mechanism underlying this paromomycin-gentamicin synergism has not been determined, possible explanations include the following:

1. Gentamicin-related attenuation of *Leishmania* parasite virulence and resultant enhancement of the development of cellular immunity ([Daneshvar et al-2003](#Daneshvar2003); [Daneshvar et al-2009](#Daneshvar2009), [Daneshvar et al-2010](#Daneshvar2010));
2. Antibacterial activity of paromomycin and gentamicin that prevents and/or cures secondary bacterial infections in CL ulcers, thereby promoting healing ([Gonçalves et al‑2005)](#Goncalves2005).
   - 1. Nonclinical Toxicology Studies of WR 279,396

Four toxicology studies (conducted in compliance with good laboratory practices) have been performed using the previous Iowa formulation of WR 279,396. Topical administration of WR 279,396 did not produce systemic effects in rats ([Levine-1995](#Levine1995)); WR 279,396 was not a dermal sensitizer in guinea pigs ([Glaza-1994b](#Glaza1994b)) and WR 279,396 did not induce primary dermal irritation in rabbits (single-dose study) ([Glaza-1995](#Glaza1995)). Although some photoactivation was noted in rabbits ([Glaza-1995](#Glaza1995)), only very weak photoallergic effects were observed in guinea pigs ([Glaza-1994a](#Glaza1994a)).

- - 1. Nonclinical Efficacy Studies of Paromomycin, Gentamicin, and WR 279,396

Paromomycin has been shown in both in vitro and in vivo to have activity against numerous *Leishmania* spp. In vitro, the ED_50_ (estimated dose for 50% inhibition of growth) values for paromomycin against amastigotes of selected Old World and New World *Leishmania* species are listed below ([Neal et al-1995](#Neal1995)):

*L major* (ED_50_ =0.9-3.9 µM; 5 strains),

*L tropica* (ED_50_ =0.9-4.9 µM; 3 strains),

*L donovani* (ED_50_ =6.1-165.7 µM; 9 strains),

*L panamensis* (ED_50_ =4.4 µM),

*L braziliensis* (ED_50_ =12.6-38.0 µM; 2 strains), and

*L mexicana* (ED_50_ =39.4 µM)

Paromomycin sulfate has not been shown in vitro to be active against *L amazonensis* under the conditions of the assay ([Neal et al-1995](#Neal1995)). There is no convincing evidence that gentamicin by itself in concentrations up to 15% has direct activity on *Leishmania* (negative studies for *L major* and *L mexicana*) ([Grogl et al-1999](#Grogl1999); [Carter et al-1989](#Carter1989)).

The combination of paromomycin with gentamicin has been shown in BALB/c mice to be more effective than either agent alone. For example, in the study results shown in Table 5, WR 279,396 was compared to paromomycin formulated with MBCL, in groups of mice infected with *L major, L mexicana, L panamensis,* and *L amazonensis* ([Grogl et al-1999](#Grogl1999)). For ulcers due to *L major* or to *L mexicana,* >90% of lesions treated with WR 279,396 or paromomycin-MBCL healed by Day 20 after therapy. No lesions treated with WR 279,396 relapsed by Day 70 after therapy, but 18% of lesions treated with paromomycin/MBCL relapsed by Day 70 after therapy. For lesions due to *L panamensis* or *L amazonensis,* all lesions treated with WR 279,396 healed by Day 20 after therapy and did not relapse by Day 70 after therapy. For lesions treated with paromomycin‑MBCL, 83% and 30% were healed by Day 20 after therapy, respectively, and all lesions relapsed by Day 70 after therapy. In addition to being active, WR 279,396 was not toxic in this model and had a cosmetic effect (promoting hair growth, healing, and limiting the size of the scar).

Table 5: Efficacy of WR 279,396 (WR) Compared with Paromomycin-MBCL-Paraffin (PM) in *L major*-, *L mexicana*-, *L panamensis*-, and *L amazonensis*‑infected BALB/c Mice

|  | *L major* | | *L major* | | *L mexicana* | | *L mexicana* | |
| --- | --- | --- | --- | --- | --- | --- | --- | --- |
|  | % Healedc | | Mean Lesion Size | | % Healeda | | Mean Lesion Sizeb | |
| Day | PM | WR | PM | WR | PM | WR | PM | WR |
| 0 | 0 | 0 | 165 | 75 | 0 | 0 | 75 | 75 |
| 10 | 70 | 93 | 25 | 40 | 80 | 93 | 15 | 10 |
| 20 | 90 | 100 | 5 | 0 | 100 | 100 | 0 | 0 |
| 30 | 100 | 100 | 0 | 0 | 100 | 100 | 0 | 0 |
| 40 | 93 | 100 | 5 | 0 | 100 | 100 | 0 | 0 |
| 50 | 96 | 100 | 15 | 0 | 100 | 100 | 5 | 0 |
| 60 | 93 | 100 | 20 | 0 | 90 | 100 | 10 | 0 |
| 70 | 82 | 100 | 25 | 0 | 83 | 100 | 25 | 0 |
|  | *L panamensis* | | *L panamensis* | | *L amazonensis* | | *L amazonensis* | |
|  | % Healeda | | Mean Lesion Sizeb | | % Healeda | | Mean Lesion Sizeb | |
| Day | PM | WR | PM | WR | PM | WR | PM | WR |
| 0 | 0 | 0 | 180 | 145 | 0 | 0 | 55 | 75 |
| 10 | 10 | 80 | 90 | 30 | 13 | 63 | 55 | 50 |
| 20 | 83 | 100 | 15 | 0 | 30 | 100 | 45 | 0 |
| 30 | 40 | 100 | 30 | 0 | 37 | 100 | 25 | 0 |
| 40 | 30 | 100 | 50 | 0 | 10 | 100 | 45 | 0 |
| 50 | 23 | 100 | 60 | 0 | 0 | 100 | 65 | 0 |
| 60 | 20 | 100 | 145 | 0 | 0 | 100 | 120 | 0 |
| 70 | 0 | 100 | 185 | 0 | 0 | 100 | 145 | 0 |

a Percentage of mice with completely healed lesions. Some mice died presumably due to natural causes: data represent mice with healed lesions/number treated (range 27-30).

b Lesion size in mm^2^.

- - 1. Clinical Studies
       1. Efficacy

The US Army has conducted one Phase 1, seven Phase 2, and one Phase 3 studies evaluating WR 279,396 to treat Old World and New World CL based on application of the drug for 20 days. Particularly relevant to the current clinical trial are a recently completed Phase 3 study in Tunisia and a Phase 2 study in Panama. The data for these two studies are presented below.

In a Phase 3 of *L major* CL in Tunisia, patients from 5 to 65 years of age with uncomplicated CL received 20 days of treatment with WR 279,396, Paromomycin Alone, or Vehicle cream (125 patients in each group). The final clinical cure rates for the two active treatment groups were nearly identical, 80.8% for the WR 279,396 group and 81.6% for the Paromomycin Alone group. The Vehicle group’s final clinical cure rate was significantly lower at 58.4% compared to WR 279,396 (p = .0001) and to Paromomycin Alone (p < .0001).

The Phase 2 in Panama was a single-site, randomized, double-blind, two group trial assessing the safety, pharmacokinetics, and efficacy of WR 279,396 compared to Paromomycin Alone in subjects with uncomplicated CL. Subjects were screened over a period up to 28 days for eligibility including parasitology for confirmation of ulcerative CL. As planned, 30 subjects were randomized in a 1:1 ratio to receive either WR 279,396 (n = 15) or Paromomycin Alone (n = 15) by topical application to CL lesions once daily for 20 days. Table 6 shows the efficacy endpoints for the Phase 2 Panama study. For the primary efficacy endpoint, subjects in the WR 279,396 group had a higher final clinical cure rate (index lesions) than those in the Paromomycin Alone group, 86.7% (13/15) versus 60.0% (9/15), respectively (chi-square test p = .099). Although this difference was not statistically significant, it was supportive of the hypothesis that WR 279,396 provides an improvement over Paromomycin Alone for curing New World CL. Secondary efficacy endpoints added further support to the improved efficacy of WR 279,396 over Paromomycin Alone. When taking all treated lesions into consideration, the final clinical cure rate for the WR 279,396 group remained the same at 86.7%; however, the rate for the Paromomycin Alone group dropped even further to 53.3% (chi-square test p = .046). The final clinical cure of all ulcerated lesions on a per lesion basis was 23/25 (92%) for the WR 279,396 group and was 19/28 (67.9%) for the Paromomycin Alone group (chi-square test p = .031).

Table 6: Primary and Secondary Efficacy Endpoints in Phase 2 Panama Study

| Endpoint | WR 279,396 | Paromomycin Alone |
| --- | --- | --- |
| Final Clinical Cure Rate – Index Lesion | 13/15 (86.7%) | 9/15 (60.0%) |
| Chi-square p-value | .099 | |
| Final Clinical Cure Rate by Subject – all Lesions | 13/15 (86.7%) | 8/15 (53.3%) |
| Chi-square p-value | .046 | |
| Final Clinical Cure rate by Lesion – all Lesions | 32/34 (94.1%) | 20/30 (66.7%) |
| Chi-square p-value | .005 | |
| Final Clinical Cure rate by Lesion – all Ulcerated Lesions | 23/25 (92.0%) | 19/28 (67.9%) |
| Chi-square p-value | .031 | |

- - - 1. Safety

In the Phase 3 study conducted in Tunisia (Protocol No. A-14134.1), WR 279,396 and Paromomycin Alone were safe, well tolerated, and showed comparable local toxicity. The majority of treatment emergent adverse events (AEs) (≥ 90%) were reported as mild, and primarily consisted of application site erythema, application site vesicles, local skin irritation, and superinfection. Of these, application site erythema (4.8%, 5.6%, and 3.2% in the WR 279,396, Paromomycin Alone, and Vehicle groups, respectively) and vesicles (24.8%, 25.6%, and 7.2% in the WR 279,396, Paromomycin alone, and Vehicle groups, respectively) were considered to be study drug related, whereas local skin irritation and superinfection were not. Systemic elicited AEs included querying the subject for evidence of vertigo or tinnitus and evaluation of serum creatinine as these are the most common side effects of concern when administering aminoglycoside antibiotics. None of these were reported in any subject. A single SAE occurred (post-streptococcal glomerulonephritis, WR 279,396 group) that was determined to be unrelated to the study drug and did not prevent the subject from either completing the study, or achieving final clinical cure of the index lesion.

In the Phase 2 study in Panama, all AEs were mild or moderate in severity with the exception of one, a migraine headache that occurred in one subject and was not considered to be related to the investigational product. The most frequently observed treatment-related AEs were application site reactions, with edema being reported in 13.3% of subjects in the WR 279,396 group and 20% in the Paromomycin Alone group. Erythema was reported in 20.0% of subjects in the WR 279,396 group and 13.3% in the Paromomycin Alone group. Application site pain was noted more frequently in the Paromomycin Alone group (33.3%) than in the WR 279,396 group (6.7%). Contact dermatitis reactions (due to the gauze and tape of the dressing) occurred about equally in both groups 40.0% for the WR 279,396 group and 53.3% for the Paromomycin Alone group. One subject in each group (two total) developed at nasal lesion (mucocutaneous CL) that was diagnosed at the Day 63 visit. Blood creatinine was measured prior to and on completion of treatment due to possible nephrotoxicity associated with aminoglycosides. Most subject’s creatinine levels remained within normal laboratory limits. When levels were outside normal laboratory limits, these levels were below the lower limit of the normal range. Elevations in serum creatinine are considered indicative of potential renal toxicity. Most subjects’ creatinine levels decreased during the study, with the highest increase just 0.1 mg/dL. Thus, there was no evidence of renal toxicity in this study.

- - - 1. Pharmacokinetics

Phase 2 studies recently completed in Peru and Panama evaluated the pharmacokinetics of WR 279,396 and Paromomycin Alone in 30 subjects with CL in each study. In the combined analysis of these two studies, three subjects treated with WR 279,396 did not have detectable levels of paromomycin on Day 1. By Day 20, all but one subject treated with WR 279,396 had detectable levels of paromomycin. Geometric mean concentrations were higher on Day 20 than those observed on Day 1. In general, plasma concentrations peaked between 1 and 4 hours after administration of the topical creams, then declined thereafter, with levels still detectable at 24 hours after administration. Following the first application of WR 279,396 (Day 1) in adults, the paromomycin AUC_(0-24)_ was 975.6 ± 1078 ng*hr/mL (Table 7). During Day 1, four subjects had no measurable paromomycin plasma concentrations. The C_max_ on Day 1 was 131 ± 112 ng/mL. On Day 20, paromomycin AUC_(0-24)_ and C_max_ estimates were greater than that noted on Day 1. On Day 20, AUC_(0-24)_ was 6037 ± 3956 ng*hr/mL and C_max_ was 660 ±486 ng/mL.

Values for the % paromomycin dose absorbed were highly variable ranging from 0.0% to 25.9%. The mean percentage of the dose absorbed was 9.68 ± 6.05% (Table 8).

At only a few collection times throughout the study did the gentamicin plasma concentrations exceed the lower limit of quantitation for total gentamicin and the isomers of gentamicin. For this reason, pharmacokinetic parameters for gentamicin could not be determined.

Table 7: Summary of Paromomycin Pharmacokinetics Following Administration of WR 279,396 Adults on Day 1 and Day 20

|  |  | Dose | CumDose | C_max_ | T_max_ | AUC_(0-24)_ | t_1/2_ | C_max_/D | AUC/D |
| --- | --- | --- | --- | --- | --- | --- | --- | --- | --- |
| **Day** | **Subject** | **(g)** | **(g)** | **(ng/mL)** | **(hr)** | **(ng*hr/mL)** | **(hr)** | **(1/ML)** | **(hr/ML)** |
|  | N | 13 | 13 | 13 | 9 | 13 | 6 | 13 | 13 |
| 1 | Mean | 2.6 | 2.6 | 131 | 2.89 | 975.6 | 4.15 | 56.6 | 344.3 |
|  | CV% | 54.7 | 54.7 | 85.7 | 76.3 | 110.5 | 35.2 | 98.5 | 89.8 |
|  | GeoMean | 2.3 | 2.3 | 35.6 | 2.33 | 125.7 | 3.96 | 18.6 | 65.69 |
|  | N | 13 | 13 | 13 | 12 | 13 | 12 | 13 | 13 |
| 20 | Mean | 3.4 | 57.8 | 660 | 2.46 | 6037 | 6.94 | 211 | 1926 |
|  | CV% | 55.4 | 43.7 | 73.7 | 53.1 | 65.5 | 47.6 | 61.5 | 76.1 |
|  | GeoMean | 2.8 | 52.1 | 354 | 2.07 | 2681 | 6.21 | 135 | 1022 |

a Cumulative dose.

b Maximum plasma concentration.

c Time of maximum plasma concentration.

d Area under the plasma concentration time curve from time 0 to 24 hours.

e Apparent terminal exponential half-life.

f Dose.

Table 8: Predicted Percentage of the Topical Dose of Paromomycin Absorbed and Paromomycin Accumulation Following Administration of WR 279,396 to Adult Subjects

| Day |  | Dose (g) | CumDose (g) | AUC_(0-24)_ (ng*hr/mL) | AUC/D (hr/ML) | Amount Absorbed (mg) | % Dose |
| --- | --- | --- | --- | --- | --- | --- | --- |
|  | N | 13 | 13 | 13 | 13 |  |  |
| 1 | Mean | 2.6 | 2.6 | 975.6 | 344.3 |  |  |
|  | CV% | 54.7 | 54.7 | 110.5 | 89.8 |  |  |
|  | GeoMean | 2.3 | 2.3 | 125.7 | 65.69 |  |  |
|  | N | 13 | 13 | 13 | 13 | 13 | 13 |
| 20 | Mean | 3.4 | 57.8 | 6037 | 1926 | 46.1 | 9.68 |
|  | SD | 1.9 | 25.3 | 3956 | 1466 | 28.7 | 6.05 |
|  | CV% | 55.4 | 43.7 | 65.5 | 76.1 | 62.3 | 62.5 |
|  | GeoMean | 2.8 | 52.1 | 2681 | 1022 | 21.2 | 4.96 |

The potential systemic absorption of and exposure to paromomycin and gentamicin suggests a very high safety margin for systemic exposure of these two aminoglycosides relative to the typical systemic exposure of patients being treated parenterally with these drugs. As expected for measurements of systemic absorption following topical administration, pharmacokinetics parameters were highly variable and may reflect the amounts of drug applied and the degree of tissue damage and accumulation. In the case of gentamicin, no clinically significant systemic absorption appeared to occur in adults or children. On Day 20, for the Paromomycin Alone group, the % dose and amount systemically absorbed were 9.68 ± 6.05% (0% to 25.9%) and 46.1 ± 28.7 mg (0 to 79.6 mg). Comparing paromomycin plasma concentrations in this study to other reported ranges for IM administration, plasma concentrations on Day 20 were 5% to 9% of those noted or expected for IM administration of paromomycin at 15 mg/kg/day to adults (the recommended dose for the systemic treatment of visceral leishmaniasis (VL). Paromomycin exposure on Day 20 was five to six times greater than at Day 1 and was probably a result of increased systemic absorption during multiple dosing due to drug saturation of skin tissue.

- 1. Known and Potential Risks and Benefits to Human Subjects
     1. Risks/Discomfort to Subjects and Precautions to Minimize Risk

Outlined below are anticipated and unexpected adverse reactions, and a brief description of procedures to ameliorate risks and symptoms. All known risks and precautions described here are explained in detail in the informed consent.

- - - 1. Skin/Local Reactions

The cream may cause skin irritations, such as vesicles, redness or pain in the area where it is applied.

- - - 1. Allergic Reactions to the Dressing

Some subjects experience allergic reactions to the dressing. A hypoallergenic tape is being used to minimize these reactions. Topical steroids may be administered to treat these local reactions.

- - - 1. Systemic Reactions: Hearing and Kidneys

The antibiotics in WR 279,396, including paromomycin, belong to a group of drugs that may affect the ear (including hearing and balance) and the kidney, if these drugs are present in the body in large amounts. However, in this study, WR 279,396 is being applied only to a small area of skin. The antibiotics should not be absorbed by the body in significant amounts, and previous studies have shown often undetectable to very low levels of drug in blood. Also, previous studies with WR 279,396 showed that the drug had no clinically significant effect on subjects’ balance, hearing, or kidney function.

- - - 1. Pregnancy

Risks to unborn babies are unknown at this time; pregnant females will be excluded from this study. Study subjects should not become pregnant for at least 1 week after the last dose of investigational product.

- - - 1. Breast Feeding

Risks to nursing infants are unknown at this time; breastfeeding females will be excluded from this study.

- - - 1. Unknown Risks

As with all research, there is the remote possibility of risks that are unknown or that cannot be foreseen based on current information.

- - 1. Research-Related Injuries

In Panama, the population is entitled to free medical care at a Regional Hospital or Heath Clinic funded by the Ministry of Health. In each region, there is a Regional Hospital and several Health Clinics affiliated with the Regional Hospital. People may go to the Regional Hospital or Health Clinic for free medical care within the region where they live.

For all routine health care needs not related to this study, the subject will receive medical care at the healthcare facility of the subject’s choice (private clinic or Ministry of Health medical facility closest to his/her residence).

In this study, subjects will participate at the Instituto Conmemorativo Gorgas de Estudios de la Salud in Panama City, at the Regional Hospital at Penonome, Cocle (Hospital Aquilino Tejeira), and at the Health Center in the town of El Silencio, near Changuinola, Bocas del Toro (Centro Materno Infantil “Sandra Hernández”). These are both the clinical trial sites and the places where subjects will receive care.

If a subject is injured as a direct result of his/her participation in this study and requires medical care for that injury, he or she will receive medical care, free of charge, at his or her participating clinical trial site as described below:

1. At the Instituto Conmemorativo Gorgas de Estudios de la Salud, which is not a hospital, subjects will be accompanied to the Hospital Santo Tomás (for adults) or the Hospital del Niño (for children). Both of these hospitals are located within 200 m of the Instituto Conmemorativo Gorgas de Estudios de la Salud.
2. At the Hospital Aquilino Tejeira en the city of Penonome (Cocle Province), subjects requiring medical care will receive care at this site.
3. At the Centro Materno Infanti Sandra Hernandez in the town of El Silencio (Bocas del Toro province), subjects requiring medical care will be accompanied to the Hospital Regional of Changuinola.

However, it is possible that the participating clinical trial site may not offer or have on hand some medicines or supplies that may be needed for the most appropriate treatment for the patient’s research-related injury. In this case, the PI of the Instituto Conmemorativo Gorgas de Estudios de la Salud has set aside $10,000 (USD) in the study contract budget to purchase drugs or other supplies, just in case they are not available.

- - 1. Alternatives to this IND Product or Study

Alternatives to this IND product are the standard treatments available in Panama. These standard treatments include observation, IV injection of antimony, or intramuscular injection of antimony at a dose of 10-20 mg/kg/day for 10-20 days.

- - 1. Intended Benefit for Subjects

Direct benefits of this study to all subjects include daily wound care during the 20 days of treatment that may reduce instances of bacterial infection, the possibility that WR 279,396 and Paromomycin Alone topical creams will cure CL lesions without injections and other systemic side effects inherent with standard of care antimonial treatment (Glucantime). Another benefit for those who do not live near a regional hospital is free transportation to and from the hospital for clinical visits. Although this study intends to show that WR 279,396 has superior efficacy compared with Paromomycin Alone, Paromomycin Alone was shown in the prior Phase 2 study to have a 60% cure rate against CL caused by *L panamensis.* An added benefit to the patient who had already failed prior antimonial therapy, could be treatment with an effective salvage therapy with either WR 279,396 or Paromomycin Alone.

- 1. Route of Administration, Dosage Regimen, Treatment Period, and Justification

Study drug will be applied topically, once daily for 20 days. The rationale is that in previous Phase 2 and Phase 3 studies of WR 279,396 with this regimen has shown good efficacy for CL.

- 1. Compliance Statement

The study will be conducted according to the protocol and in compliance with International Conference on Harmonization (ICH) Good Clinical Practice (GCP), Belmont Principles, and other applicable US/Panama regulatory and DoD requirements. All identified study personnel will be trained to perform their roles and will carry out their responsibilities in accordance with ICH GCP guidelines and clinic site study specific procedures (SSPs). Roles and responsibilities of study staff are presented in Appendix A.

- 1. Study Population

The study population includes a total of 400 eligible patients 2 years of age and older with a parasitological confirmation of CL by microscopic evaluation of lesion scrapings that was positive by culture for promastigotes or visualization of amastigotes in stained smears on slides. Refer to section 12.2 for a statistical justification of the sample size.

- 1. Study Sites

The study will be conducted at 3 sites: Instituto Conmemorativo Gorgas de Estudios de la Salud, Panama City, Hospital Aquilino Tejeira, Penonome (Cocle), and Centro Materno Infantil “Sandra Hernández”, El Silencio, Changuinola (Bocas del Toro). The sites in Penonomé and El Silencio are considered satellite sites to the site in Panama City, where the study will be conducted under the direction of the PI, Dr. Sosa.

1. Trial Objectives and Purpose
   1. Primary Objectives

The primary objective of this study is to determine if WR 279,396 results in statistically superior final clinical cure rates of an index lesion when compared with Paromomycin Alone for the treatment of CL in Panama expected to be caused by *L panamensis*.

- 1. Secondary Objectives

Secondary objectives include evaluating other efficacy parameters including: 1) percentage of subjects with all lesions cured; 2) percentage of all lesions cured; 3) lesion sizes over time; 4) cure rates over time, and 5) median time to initial cure to determine if these parameters are superior in the WR 279,396 group compared with the Paromomycin Alone group. An additional secondary objective is to determine the safety of both topical creams in this study population.

1. Trial Design

This study is a pivotal Phase 3, randomized, double-blind, 3-site, two-group trial assessing the efficacy and safety of WR 279,396 Topical Cream and Paromomycin Alone Topical Cream in subjects with CL in Panama. Subjects will be screened over a period up to 28 days for eligibility including parasitology for confirmation of ulcerative CL. If eligible, subjects will be randomized using site as a stratification variable in a target ratio of 1:1 to receive either WR 279,396 (15% paromomycin + 0.5% gentamicin topical cream) (target n=200) or Paromomycin Alone (15% paromomycin topical cream) (target n=200) by topical application to CL lesions once daily for 20 days. Safety will be assessed by monitoring AEs, lesion site reactions, and blood creatinine, aspartate aminotransferase (AST), and alanine aminotransferase (ALT) levels.

After completing treatment, subjects will have an in-clinic follow-up on Study Days 35± 2 days, 49± 4 days, 63± 7 days, 100± 14 days, and 168 ± 14 days. Follow-up evaluations on 63 ± 7 days, 100 ± 14 days, and 168 ± 14 days include assessment of AEs, medication use, lesion measurements, physical examination of the oral and nasal mucosa for presence of mucosal leishmaniasis, and lesion photographs.

The primary efficacy analysis will be by evaluation of an index lesion with secondary efficacy analyses including all lesions. Efficacy will be assessed by measuring the size of the index lesion ulcer, non-index lesions ulcers, and overall size of other non-ulcerated lesions at baseline (before the start of treatment), and on Study Days 20, 35 ± 2 days, 49 ± 4 days, 63 ± 7 days, 100 ± 14 days, and 168 ± 14 days. A notation will be made if clinical evidence of parasite persistence is observed at the Day 63 and beyond visits including significant erythema and induration when a lesion has otherwise completely re-epithelialized to document any subjects removed from the study early if the investigator judges them to be in need of rescue treatment.

- 1. Study Endpoints
     1. Primary Efficacy Endpoint

The primary efficacy endpoint is percentage of subjects with final clinical cure. **Final clinical cure** is defined as follows:

- Subject has initial clinical cure (100% re-epithelialization of index lesion by nominal Day 63); OR,
- Subject has initial clinical improvement (> 50% re-epithelialization of index lesion by nominal Day 63) followed by 100% re-epithelialization of the index lesion on or before nominal Day 100; AND,
- Subject has no relapse of index lesion.

**Relapse** is defined as an index lesion meeting the criteria for initial clinical cure that had any new ulceration/nodule (> 0 x 0 mm measurement) by nominal day 168, or an index lesion meeting the criteria for initial clinical improvement that subsequently enlarged by nominal Day 168.

**Failure** is the opposite of cure: Subjects who drop out of the study early (ie, are not assessed at the final Day 168 visit) will also be considered clinical failures.

In addition, a notation will be made if clinical evidence of parasite persistence is observed at the Day 63 and beyond visits including significant erythema and induration when a lesion has otherwise completely re-epithelialized to document any subjects removed from the study early if the investigator judges them to be in need of rescue treatment to document reason for clinical failure when complete re-epithelialization is reported.

- - 1. Secondary Efficacy Endpoints

Secondary efficacy endpoints are as follows:

- Percentage of subjects with all lesions cured, defined as:
  - Final clinical cure as defined above (which is based solely on the index lesion); AND,
  - Cure of all other lesions by nominal Day 100 (100% re-epithelialization of all ulcerated lesions and resolution of all other types of lesions)
- Percentage of all lesions cured at Day 168 (ignores per subject cure rate)
- Area of ulceration of the index lesion at each measurement time point
- Area of ulceration all treated lesions at each measurement time point
- Ulcerated lesion cure rate at each measurement time point (cure is defined as 100% re-epithelialization of an ulcerated lesion)
- Median time to initial clinical cure (100% re-epithelialization of the index lesion)
  - 1. Safety Endpoints

Safety endpoints are as follows:

- AEs including application site reactions including elicited examination for pain, and clinician examination for erythema/redness, swelling/edema, and vesicles. Physical examination findings of evidence of mucosal leishmaniasis will be reported as an AE.
- Blood creatinine, AST, and ALT
  1. Overall Study Design

The trial design is illustrated in Table 9.

- 1. Measures Taken to Minimize/Avoid Bias

Subjects will be randomized sequentially at each site to blinded treatment with WR 279,396 or Paromomycin Alone. This study will be double-blinded; therefore, tubes of both creams will be indistinguishable and their contents will appear identical. Neither the subject nor the investigative team will know the treatment to which the subject is assigned.

Table 9: Schedule of Procedures

|  | Screening | Treatment Period | | | Follow-up Period | | | | |
| --- | --- | --- | --- | --- | --- | --- | --- | --- | --- |
| Procedure | -28 to -1 | Day -1/1 | Day 2-19 | Day 20 | Day 35 ± 2 | Day 49 ± 4 | Day 63 ± 7 | Day 100 ± 14 | Day 168 ± 14 |
| Informed consent/assent | X |  |  |  |  |  |  |  |  |
| Demographics | X |  |  |  |  |  |  |  |  |
| Medical history | X |  |  |  |  |  |  |  |  |
| Leishmaniasis history | X |  |  |  |  |  |  |  |  |
| Physical examination | X |  |  |  |  |  | X | X | X |
| Parasitology | X |  |  |  |  |  |  |  |  |
| Vital signs | X |  |  |  |  |  |  |  |  |
| Lesion measurements | X | X |  | X | X | X | X | X | X |
| Clinical chemistry | X |  |  | X |  |  |  |  |  |
| Pregnancy test | X | X |  |  | X |  |  |  |  |
| Eligibility checklist |  | X |  |  |  |  |  |  |  |
| Randomization |  | X |  |  |  |  |  |  |  |
| Study drug application |  | X | daily | X |  |  |  |  |  |
| Photograph of lesions |  | X |  | X | X | X | X | X | X |
| Adverse events |  | X | daily | X | X | X | X | X | X |
| Prior and concomitant medications | X | X | once per week | X | X | X | X | X | X |

a Physical examination includes a general physical exam and at baseline and follow-up an intensive evaluation of the mucosa.

b Parasitology includes smear and culture of lesion scrapings/aspirates for promastigotes, microscopic identification of *Leishmania* amastigotes on Giemsa stain, and PCR.

c Vital signs include oral temperature, and sitting blood pressure and heart rate.

d Clinical chemistry measurements include creatinine, AST, and ALT during screening and Day 20. After the sponsor’s approval, biochemistry can be repeated in the case of abnormal results and if the causes of these results could not be determined.

e On Day 1.

- - 1. Blinding

This study is double-blinded (neither the investigative team nor the subject will know the identity of the investigational product that they are receiving). Blinded study supplies will be provided to the clinical site coded only by a randomization number.

For the final analysis, a formal approved statistical analysis plan (SAP) will be in place before study data is unblinded for the analysis. The database will be locked for both of these analyses before unblinding takes place. Any adjudication of data and address of missing data plans will be performed while the data is still blinded. Any changes to the SAP after un-blinding has occurred will be documented in the final clinical study report.

- 1. Investigational Product
     1. Description

The investigational products are WR 279,396 Topical Cream and Paromomycin Alone Topical Cream. Both are manufactured by Teva Pharmaceuticals USA, 650 Cathill Road, Sellersville, Pennsylvania, 19454 for USAMMDA. The lot number is stamped on the tube crimp; however, for blinded studies, the lot number is obscured to preserve the study blind. Each gram of WR 279,396 cream contains 150 mg (15% w/w) paromomycin USP base and 5 mg (0.5% w/w) gentamicin USP base. Each gram of Paromomycin Alone cream contains 150 mg (15% w/w) paromomycin USP base. Additional components of the emulsion include emollients and emulsifiers, a humectant and acidifying agent, a nonionic detergent, a surfactant, and various other inactive ingredients which comprise a proprietary formulation. This formulation also includes urea 6.75%, which is believed to facilitate the penetration of paromomycin and gentamicin into the dermal lesion where the intracellular parasites reside. Urea is also thought to act as a moisturizer and keratolytic agent.

- - 1. Investigational Product Packaging and Labeling

The investigational product is packaged in white, unprinted blind-end aluminum tubes (7/8″ diameter x 5″ long) with taper-piercing unlined plastic caps. The internal liner of the tube is PE‑1090-21 (no wax).

Investigational products (tubes of cream) will be provided to the clinical site with the following label:

Randomization#: XXXX
 Subject ID #: ________________
 Subject Alpha ID: ____________
 Storage: 2-8˚C Tube: A
 Protocol No.: S-12-21
 WR# 279396
 USAMRMC, Fort Detrick, MD, USA
 CAUTION: New drug - limited by Federal law to investigational use only

*XXXX is a 4-digit sequential number assigned to 3 tubes of investigational product for each subject. Tubes are designated A, B, or C – which will be used as the unique tube identifier.

Three tubes with each randomization number will be provided to the clinical site. The label described above will have the following information written in the appropriate spaces by study staff member who performed the randomization: the subject identification (ID) number and alpha code.

- - 1. Investigational Product Storage

Investigational Products should be stored at 2° to 8°C in monitored and secure refrigerators at the clinical site. Tubes of drug will be removed from the refrigerator, the drug will be applied to the subject’s lesions, and then tubes will be returned to the refrigerator.

- 1. Duration of Subject Participation

Each subject will participate in the study for about 196 days (up to 28 days for screening and 168 days for treatment and follow-up).

- 1. Dose-adjustment Criteria
     1. Safety Criteria for Dose Adjustment or Stopping Doses

If one study-drug-related serious or unexpected AE evaluated by the principal investigator, local research monitor, and sponsor’s representative and determined to be an unacceptable risk to the health and safety of other investigational product recipients occurs, administration of investigational product will be discontinued. Administration will not resume until a thorough review of the events is undertaken by the investigators, the Gorgas Institutional Bioethics Committee which is the Ethics Committee of record, local research monitor, and/or sponsor’s representative.

- - 1. Study Termination

The principal investigator, local research monitor, sponsor’s representative, the Gorgas Institutional Bioethics Committee, the United States Army Medical Research and Materiel Command (USAMRMC) Office of Research Protections, Human Research Protection Office (ORP HRPO), or the FDA may stop or suspend the use of this product at any time.

- 1. Investigational Product Accountability

The clinical site will be provided forms for study drug accountability and application. The following information will be recorded on the forms supplied by the sponsor:

- Total amount of the study drug received from the sponsor (number of tubes, date of receipt, condition of containers on receipt, location and conditions of storage until use in clinic)
- Identification (study number and initials) of the subject to whom the study drug was applied
- Date the study drug is applied to each lesion to the subject
- Initials of the person who dispensed the study drug and the initial pre-weight of tubes
- Initials of the person who received the study drug for administration to the subject
- At the end of the treatment period, all tubes must be returned for a post-administration weight determination.

The investigator is responsible for ensuring all study drug accountability. Any discrepancies must be described in writing. Records for dispensing study drug must be available for inspection by the clinical study monitor throughout the study. All tubes (unused, partially used, and spent) will be retained by the study staff for accountability. No tubes should be destroyed or disposed without specific instructions from the sponsor. This occurs after the study monitor has completed the final accountability inspection. The disposition records will account for all remaining investigational products.

All clinical supplies and investigational products intended for use in the clinical study cannot be used, under any circumstances, for any purpose other than that described in the protocol.

- 1. Trial Treatment Randomization Codes

A permuted block randomization method will be used to generate the treatment randomization which will be performed by the Data Management Center. As the study will be conducted at three sites in Panama; the site will be used as stratification variable for randomization. A site-specific randomization list will be maintained at each clinical site that contains the study drug kit number to be assigned to each sequential subject that is eligible for the study. A Master Randomization List will also be maintained at the Data Management Center. The list maintained at the Data Management Center will also contain the identity of the investigational product associated with each randomization number. The identity of the investigational product associated with each randomization number will be maintained blinded to the rest of the study team, by the Data Management Center.

If insufficient study drug is provided in a single kit of three tubes of drug, then the site coordinator will contact USAMMDA or the Data Management Center for assignment of an additional drug kit for the subject.

- 1. Identification of Data to be Recorded on the Case Report Forms

No source data will be recorded directly on the case report form (CRF) without prior written or record of data. The transcribed data will be consistent with the source documents or the discrepancies will be explained. For more information on data handling, refer to section 16.

1. Selection and Withdrawal of Subjects

Recruitment of Subjects

Potential subjects will be recruited from three areas in Panama know to be prevalent with *L panamensis* CL: 1) Panama City, 2) Penonome (Cocle), and 3) El Silencio, Changuinola (Bocas del Toro). Interested candidates who are seeking treatment for suspicious CL lesions will meet with the investigator or designated investigational staff to receive an explanation of the study purpose and requirements. If still interested after receiving an explanation of the study, the candidate or legal guardian will be given an opportunity to review, inquire about, and sign the informed consent form or assent form, as applicable. A copy of the signed consent/assent form will be provided to the subject.

Subjects who sign informed consent/assent and fail to meet the inclusion and/or exclusion criteria are defined as screen failures. The investigator will maintain a screening log that documents the Subject ID number, Alpha Code, and if a subject was randomized and enrolled to the study or was a screen failure. A screen failure Case Report Form (CRF) will be completed for all subjects who do not meet study eligibility criteria to capture the reason that the subject was not eligible for the study.

Informed Consent Process

Interested subjects who will be available during the study period will be invited to have a meeting with the study team, principal investigator, and/or Subinvestigators for a more detailed explanation about the protocol and to receive a copy of the informed consent form to read. If the subject would like to go home with the consent and return, this is encouraged. The subject is also encouraged to read the informed consent form carefully and to ask for any clarification of the material they read before signing the form.

No study related evaluations or procedures will be conducted until the subject has read and signed an informed consent form that has been approved by the Gorgas Institutional Bioethics Committee and USAMRMC ORP HRPO.

- 1. Eligibility Screening

Subjects properly consented to the study will have the following data collected after informed consent and prior to the initiation of study cream application. Subjects will be notified if any completed tests or examinations exclude them from the study, and recommendations for referral will be made as appropriate.

- - 1. Demographics

Demographic data including the subject’s sex, date of birth, ethnicity and race will be collected. Contact information will be collected and kept only in the subject’s medical record.

- - 1. Medical History

To monitor the health of all study subjects, medical history will be collected prior to participation in the study. A review of systems will be conducted by the site principal investigator/study physician to assure medical fitness.

- - 1. Physical Exam

A physical exam of the oral cavity; head; eyes; ears; nose; and throat; cardiovascular system; lungs; abdomen (liver/spleen); extremities; skin; lymphatic system; neuropsychiatric mental status and sensory/motor status; musculoskeletal system; and general appearance will be performed during screening. In addition, the principal investigator or designee will examine the nasal and oral mucosa of each subject. Each of 5 possible sites (nasal skin, nasal mucosa, palate, pharynx, and larynx) will be evaluated for 4 possible signs of disease (erythema, edema, infiltration, and erosion). To be eligible for the study, no sign of disease can be present at any of the sites. The exam of the lymphatic system will focus on particularly on lymph nodes in the drainage area of the ulcer. The examination of the nasal and oral mucosa will be repeated at follow-up visits scheduled at Days 63 ± 7 days, 100 ± 14 days, and 168 ± 14 days.

- - 1. Pregnancy Test

A serum pregnancy test that measures human β-chorionic gonadotropin will be used for women of child-bearing potential during screening, at day -1/1 (only if the preceding pregnancy test exceeds 24 hours), and at Day 35 ± 2 days.

- - 1. Vital Signs

Vital signs to be checked during screening include tympanic temperature and sitting blood pressure and heart rate.

- - 1. Blood for Chemistry and Pregnancy Tests

All adult subjects will have one tube of approximately 8 mL of blood for clinical chemistry, including creatinine, ALT, and AST, collected during screening and another tube of approximately 8 mL at Day 20. The screening clinical chemistry evaluation may be repeated if the results are abnormal without an assignable cause, with the written permission of the sponsor. The results of the initial test and repeat tests will be recorded on the subject’s CRF.

If the subject is female of child-bearing potential, a serum pregnancy test will be performed and the tube collected for clinical chemistry during screening will also be used for the pregnancy test. However, if the pregnancy test obtained during screening was done more than 24 hours before Day -1/1, an additional 8 mL of blood will be needed for another pregnancy test on Day -1/1. In addition, another 8 mL will be collected for a final pregnancy test at Day 35 ± 2 days.

For children, the same procedures will apply using 3 mL of blood.

- - 1. Leishmaniasis History

During screening, the subject will be queried about the time of appearance of the oldest current lesion, any past history of CL, and any prior and any current treatments for CL.

The size (length/width) of each lesion will be recorded as well as other characteristics of the lesion (ulcerated versus non-ulcerated), and anatomical location (see Body Chart for numbering system in Appendix B).

- - 1. Diagnosis of Leishmaniasis

The investigator will select ulcerative lesion(s) on the subject and decide on the appropriate method for sample collection (scraping versus aspiration). The lesion must be sampled during the 28-day period before starting investigational product application. The majority of the time, it is expected that specimens will be collected by scraping. Specimens will be smeared onto microscope slides, inoculated in culture medium, and analyzed by the Instituto Conmemorativo Gorgas de Estudios de la Salud. Proof of infection due to *Leishmania* will be documented through either the demonstration of motile promastigotes in culture medium or microscopic identification of *Leishmania* amastigotes by DifQuik or Giemsa staining. Whenever possible, species identification will also be performed using isoenzyme analysis or PCR. PCR analysis will be performed by the Instituto Conmemorativo Gorgas de Estudios de la Salud. In addition, parasite DNA samples may be sent to Department of Parasitology, U.S. Naval Medical Research Unit No. 6, Lima, Peru for FRE-based real time PCR assay. Isoenzyme analysis will be performed by Walter Reed Army Institute of Research, Silver Spring, Maryland.

- 1. Eligibility Checklist

An eligibility checklist will be reviewed upon completion of screening (on or before Study Day 1) and must be completed before the start of treatment and signed and dated by the principal investigator or a subinvestigator.

- 1. Subject Inclusion Criteria

Subjects must meet all of the following criteria to be included in the study:

- Male or female at least 2 years-of-age
- Subject or legal guardian able to give written informed consent or assent, as appropriate
- Diagnosis of CL in at least one lesion by at least one of the following methods: 1) positive culture for promastigotes or 2) microscopic identification of amastigotes in stained lesion tissue
- At least one ulcerative lesion ≥ 1 cm and ≤ 5 cm that has a diagnosis of CL
- Willing to forego other forms of treatments for CL including other investigational treatments during the study
- In the opinion of the investigator, the subject (or their legal guardian) is capable of understanding and complying with the protocol
- If female of child-bearing potential, must have a negative serum pregnancy test during screening and agree to use an acceptable method of birth control during the treatment phase and for 1 week after treatment is completed
  1. Subject Exclusion Criteria

Subjects meeting any of the following criteria will be excluded from the study:

- Lesion due to *Leishmania* that involves the nasal or oral mucosa or any signs of mucosal disease that might be due to *Leishmania*
- Only a single lesion on the ear with erosive cartilage
- Signs and symptoms of disseminated disease in the opinion of the investigator
- More than 10 lesions
- Female who is breast-feeding or pregnant
- Significant organ abnormality, chronic disease such as diabetes, severe hearing loss, evidence of renal or hepatic dysfunction, or creatinine, aspartate aminotransferase (AST), or alanine aminotransferase (ALT) greater than 15% above the upper limit of normal (ULN) as defined by the clinical laboratory defined normal ranges
- Received treatment for leishmaniasis including any medication with pentavalent antimony including sodium stibogluconate (Pentostam), meglumine antimoniate (Glucantime); amphotericin B (including liposomal amphotericin B and amphotericin B deoxycholate); or other medications containing paromomycin (administered parenterally or topically) or methylbenzethonium chloride (MBCL); gentamicin; fluconazole; ketoconazole; pentamidine; miltefosine, azithromycin or allopurinol that was completed within 56 days of starting study treatments
- After the sponsor’s approval, biochemistry can be repeated in the case of abnormal results and if the causes of these results could not be determined
- History of known or suspected hypersensitivity or idiosyncratic reactions to aminoglycosides
- Any other topical disease/condition which would interfere with the objectives of this study

NOTE: The index lesion size for study inclusion is maximum diameter of the ulceration and surrounding induration.

- 1. Subject Withdrawal Criteria

Each subject may withdraw consent at any time during the study without penalty. Counseling about the subject's health will be provided if he/she decides to discontinue participation in the study. Medical advice regarding what is in the best interest of the subject will be provided.

The PI may discontinue the subject’s activity without the subject’s consent if any of these criteria is met:

- A subject fails to comply with study procedures
- A subject’s safety or health may be compromised by further participation
- A subject’s CL lesion fails to respond to treatment [eg, lesion enlargement by Day 63 or evidence of clinically significant erythema with induration (in the judgment of the investigator) at Day 100 even if the ulcer has healed].

NOTE: If the subject met the criteria for therapy failure but was undergoing treatment for new lesions, the subject can continue in the study through Day 168, if the investigator decides it is in the best interest of the subject to do so and the subject signs a consent addendum.

- - 1. When and How to Withdraw Subjects

A subject may withdraw, or be withdrawn by a parent or legal guardian, from the study at any time and for any reason without penalty, if he or she wishes to do so. The investigator will make a reasonable effort to determine the reason for the withdrawal from the study and to complete termination procedures as described in section 8.5.4. Reasonable efforts to contact the subject include telephone calls or contact with the subject at their home for those subjects typically driven to clinic appointments who do not have telephones.

The investigator may also withdraw a subject for reasons including, but not limited to: if continuing participation is believed to be harmful to the subject’s well-being, protocol violations, non-compliance, positive pregnancy test during active treatment, serious intercurrent illness, or the need to initiate rescue therapy using alternative therapies for treatment of CL lesions.

Note: Subjects who fail therapy will be taken off study and referred to their personal physician for alternative treatments except in the case as described in the Note above (section 8.5). Another option for subjects who fail therapy is to receive standard of care treatment (Glucantime™) provided by the study team. If a subject chooses to receive standard of care treatment with Glucantime, this will be noted in the subject’s research records and reported on the Subject Disposition CRF.

If a subject meets withdrawal conditions for a concomitant medication violation or noncompliance, this should clearly be stated in the source document and the study termination CRF.

When a subject withdraws due to an AE or is withdrawn by the principal investigator due to an AE, the USAMRMC Regulatory Affairs must be notified within 72 hours at (usarmy.detrick.medcom-usammda.mbx.usamrmc-regulatory-affairs@mail.mil). Investigators must follow specific policy at each institution regarding the timely reporting of AEs and SAEs to the Gorgas Institutional Bioethics Committee (section 11.5.1.2). In all cases, the principal investigator will make a reasonable effort to complete study termination procedures.

- - 1. Data Collected for Withdrawn Subjects

All data collected up to the time of withdrawal will be reported. The subject disposition CRF will be completed, with the reason for withdrawal specified.

- - 1. Replacement of Subjects

Not applicable.

- - 1. Follow-up for Withdrawn Subjects

In the event that the subject withdraws from the study before the completion of the 20 days of treatment, s/he will be asked to come to the clinic for a final assessment including AEs, lesion measurements, and concomitant medications use. If the subject withdraws during the follow-up period, s/he will be contacted, if possible, and requested to come to the clinic for evaluation. If a female subject becomes pregnant during the 20 day treatment period through the Day 28 follow-up (the time frame during which she must use birth control), if possible, the pregnancy should be followed until the end of the pregnancy. Should a female become pregnant after the Day 28 follow-up, the study would not require the pregnancy to be followed.

1. Treatment of Subjects
   1. Baseline Assessments Prior to Application of the Study Drugs

The following will be done on study Day -1/1 prior to investigational product application:

- Baseline assessment of lesions and surrounding skin for future assessments of application site reactions including severity of pain, erythema/redness, swelling/edema, and vesicles.
- Lesion measurements
- Record changes in concomitant medications
- For female subjects of child-bearing potential, repeat pregnancy test if screening test was performed more than 24 hours prior to the start of treatment
- Complete eligibility checklist
- Randomization
- Study drug application (Day 1)
- Assess and record and AEs after treatment (Day 1)
  - 1. Identification of the Index Lesion

The index lesion chosen for primary efficacy analysis will be selected according to Figure 1. Briefly, the index lesion will be the one that is the uppermost on the body, primarily ulcerative, and parasitologically positive. If two lesions are equally uppermost, the subject’s left uppermost primary ulcerative lesion will be selected. If a subject has a single lesion on the ear with erosive cartilage, then this lesion is not amenable to topical treatment and the subject should be excluded from the study. There is a preference for non-facial and non-ear lesions as the index lesion.

Measurement of possible index lesions for study inclusion is the maximum diameter of the ulceration and surrounding induration. Thereafter, the size of ulcerated lesions will only be the largest diameter of the ulcer.

Figure 1: Decision Tree for Selection of the Index Lesion

- - 1. Lesion Photographs

After cleaning and debriding all lesions, all lesions will be photographed with a digital camera for a record of its baseline appearance and appearance at all scheduled and any unscheduled follow-up visits.

- 1. Application of the Study Drug

The investigational cream to which the subject was assigned will be applied topically to all lesions once daily through Day 20. Application of investigational cream daily to Day 20 will be continued even if the lesion has obtained 100% re-epithelialization prior to Day 20. A local anesthetic cream may be applied or lidocaine injection may be performed before the cleaning and debridement procedure to minimize pain. Prior to the first application, the lesions will be cleaned and debrided and then dried. At each subsequent application of the investigational cream, the previous day’s application and dressing will be removed, the lesion will be cleaned with soap, water, and sterile 0.9% saline, then dried using sterile cotton gauze sponges, and re-dressed. Lesions will be left undisturbed until the next application. The investigational cream will be applied with a gloved finger to cover the area of the ulcer or raised area of non-ulcerated lesions and rubbed into the lesion. The weight of the 3 tubes in one drug kit will be taken through the use of an analytical balance before the start of treatment and again after completing the 20 days of treatment. The difference in weight will indicate how much investigational cream was applied. Each site will possess an analytical balance.

If the subject develops a new lesion(s) during the study which can undergo and complete 20 days of treatment prior to Day 168, the new lesion(s) can be treated with investigational cream once daily for 20-days as long the Principal Investigator judges the overall clinical picture of the subject and the new lesion(s) to be amenable to treatment. The new lesion will be cleaned, debrided, and measured before the start of treatment, at the end of treatment, then again at every regularly scheduled visit. At the end of this treatment course, the tubes will be weighed again to determine the total amount of cream applied. A separate CRF will be completed for data collected for new lesions that are treated.

- 1. Instructions for Subjects

Subjects will be instructed to leave the dressing in place after daily application until the next visit either at the clinic or at another location (e.g. at the subject’s home or at a relative’s home), and to keep the dressing as dry as possible (no swimming). Daily bathing is best performed in the morning before coming to the clinic for daily application of study drug without concerns of wetting the dressing or lesion area. Saran or plastic wrap may be used to protect the dressing, if the subject is performing an activity other than daily bathing during which s/he suspects that the dressing may become wet. Nevertheless, subjects will be instructed that occlusion of the lesion via plastic wrap should only be used in these unusual circumstances and should not be left in place after completing these activities. As above, it is acceptable to bathe without the plastic covering in the morning before the clinic appointment to have the dressing changed.

- 1. Concomitant Medications

All medications including over-the-counter products taken by the subject 56 days before signing consent, after consent during screening and up to the Day 168 follow-up assessment will be recorded on a Prior and Concomitant Medications CRF. Subjects must be willing to forego other treatments for CL including other investigational treatments during the study.

The following medications are also prohibited from 56 days prior to the start of the study and through the final follow-up (Day 168): any medication with pentavalent antimony including stibogluconate sodium (in any form such as Pentostam), meglumine antimoniate (Glucantime); amphotericin B including liposomal amphotericin B and amphotericin B deoxycholate; or other medications containing paromomycin (administered parenterally or topically), or miltefosine.

- 1. Procedures for Monitoring Subject Compliance

A record will be made of daily application of the study drug by the study staff.

- 1. Study Days 2 through 19

The following will be done:

- Assess and record AEs and concomitant medications
- Examine lesions and surrounding skin for application site reactions including: severity of erythema/redness and swelling/edema, vesicles, and pain
- Clean lesions, apply investigational product to lesions, and record time
  1. Study Day 20

The following will be done:

- Assess and record AEs and concomitant medications
- Examine lesions and surrounding skin for application site reactions including: severity of erythema/redness and swelling/edema, and pain
- Measure lesions
- Collect blood for clinical chemistry
- Clean lesions, apply investigational product to lesions, and record time
- Photograph lesions
- Weigh investigational product tubes after the last application
  1. Study Days 35 ± 2 days and 49 ±4 days

The following will be done:

- Assess and record AEs and concomitant medications
- Examine lesions and surrounding skin for application site reactions including: severity of erythema/redness and swelling/edema, and pain
- Measure lesions
- Photograph lesions
- Collect blood for pregnancy test (for female subjects of childbearing potential on Day 35 ± 2)
  1. Study Days 63 ±7 days, 100 ± 14 days and 168 ± 14 days

The following will be done:

- Assess and record AEs and concomitant medications
- Physical examination of mucosa and palate
- Examine lesions and surrounding skin for application site reactions including: severity of erythema/redness and swelling/edema, and pain
- Measure lesions
- Examine lesions and make a notation whether subject is to be withdrawn from the study early due to apparent lack of clinical cure indicated by significant induration and erythema of the lesion in the absence of an open ulcer
- Photograph lesions

1. Efficacy Assessments
   1. Specification of Efficacy Endpoints

Primary and secondary endpoints are listed in sections 7.1.1 and 7.1.2, respectively.

- 1. Methods/Timing for Assessing, Recording, and Analyzing Efficacy Endpoints

The length and width of all ulcerated lesions will be measured in millimeters (mm) on Day 1 prior to the start of treatment and on nominal Study Days 20, 35, 49, 63, 100, and 168. Non-ulcerative lesions will also be measured, and the length and diameter of these lesions will be reported (these data will be used to calculate exposure surface area). Non-ulcerated lesions have the appearance of a fried egg with a raised center that may be verrucous with a surrounding area of swelling or erythema. The raised center of these lesions will be the areas to be treated and measured. Digital pictures of the index lesion will be taken prior to therapy, at the end of therapy (Day 20), and at all other study visits after this time. A metric ruler will be placed adjacent to each lesion and a digital picture taken. If a lesion has healed, the ruler will be placed adjacent to the area of the prior lesion and the digital picture will be taken. Physical features identifying the subject, if present, will be hidden in digital pictures. The subject’s ID code, lesion number, Study Day and date will be recorded on a label which will be placed near the lesion and included in the digital picture. Digital images files will be maintained locally and transferred to the Data Management Center for storage in the main study database, and later provided to the sponsor’s representative.

The area of ulceration will be calculated using the area calculation for an ellipse as follows:

Area = A/2*B/2*Pi mm^2^

Where A = longest diameter of ulceration in mm

B = perpendicular to “A” diameter of ulceration in mm

Pi = 3.1416

The same area calculation applies to non-ulcerated lesions for surface exposure measurements.

1. Safety Assessments

Safety monitoring will be conducted throughout the study; therefore safety concerns will be identified by continuous review of the data by the principal investigator, study staff, clinical monitor, research monitor, and USAMMDA CSSD.

**Study Safety Management:** A data safety monitoring board (DSMB) is not required for this study.

**Research Monitor:** The research monitor will function as an independent safety advocate for subjects per AR 70-25, DoD Instruction 3216.02. The independent research monitor is required to review all unanticipated problems involving risk to subjects or others, SAEs, and all subject deaths associated with the protocol and provide an unbiased written report of the event. At a minimum the research monitor should comment on the outcomes of the event or problem and, in the case of an SAE or death, comment on the relationship to participation in the study. The research monitor should also indicate whether he/she concurs with the details of the report provided by the study investigator. Reports for events determined by either the investigator or research monitor to be possibly or definitely related to participation and reports of events resulting in death should be promptly forwarded to the Gorgas Institutional Bioethics Committee, USAMMDA Safety Surveillance Branch, ORP HRPO, and USAMMDA Regulatory Affairs.

**USAMMDA CSSD:** CSSD is responsible for coordinating and integrating the review of safety data regarding The Surgeon General, Department of the Army IND-sponsored products. The Product Safety Surveillance Branch reviews each SAE report for medical consistency, accuracy, and completeness and follows each event until it is satisfactorily resolved.

- 1. Specification of Safety Endpoints

**Adverse Events.** AEs will be assessed by asking subjects general questions about their well-being at each study visit, by assessing lesions during treatment, by evaluating clinical chemistry measurements at the end of treatment, and by physical examination of the mucosa and palate for evidence of mucosal leishmaniasis. A research nurse, physician, or medically trained staff will assess AEs. If an AE is reported that requires medical attention, it should be reported to a study physician immediately. The type of AE, severity of the AE and the relationship of the AE to the investigational product will be recorded on the source document and on an AE CRF, according to the procedures described in section 11.4.3.

**Application Site Reactions.** Prior to and during treatment, application site areas will be examined for the appearance of swelling/edema, vesicles, and erythema/redness and presence or absence of these reactions and an assessment of their severity. The subject will be asked if the lesions are painful prior to each day’s drug application. If evidence of worsening from baseline is observed, the change from baseline will be recorded as an AE. The assessment of the severity of these AEs will be recorded in accordance with the criteria in section 11.4.3. The worst severity of any lesion will be recorded.

- 1. IND Safety Reporting

The following terms, as defined by 21 CFR 312.32, apply to IND safety reporting.

- - 1. Adverse Event or Suspected Adverse Reaction

AE means “any untoward medical occurrence associated with the use of a drug in humans, whether or not considered drug related.”

Suspected adverse reaction means any adverse event for which there is a reasonable possibility that the drug caused the AE. For the purposes of IND safety reporting, “reasonable possibility” means there is evidence to suggest a causal relationship between the drug and the AE. Suspected adverse reaction implies a lesser degree of certainty about causality than adverse reaction, which means any AE caused by a drug.”

For this study, AEs will include events reported by the subject, as well as clinically significant abnormal findings on physical examination or laboratory evaluation. A new illness, symptom, sign or clinically significant clinical laboratory abnormality or worsening of a pre-existing condition or abnormality is considered an AE. Stable chronic conditions which are present prior to clinical trial entry and do not worsen are not considered AEs. All AEs must be recorded on the AE CRF.

Application site reactions will be recorded as an AE; however, typical weeping, scabbing, and scaling appearance of CL lesions are not considered an AE, unless these significantly worsen and in the judgment of the Investigator are clinically significant. The appearance of scars at healed lesion sites will not be reported as an AE.

- - 1. Serious Adverse Event or Serious Suspected Adverse Drug Reaction

An AE or suspected adverse reaction is considered “serious” if, in the view of either the investigator or sponsor, it results in any of the following outcomes:

- Death
- Life-threatening adverse event
- Inpatient hospitalization or prolongation of existing hospitalization
- Persistent or significant incapacity or substantial disruption of the ability to conduct normal life functions
- Congenital anomaly/birth defect.

An AE or suspected adverse reaction is considered “life-threatening” if, in the view of either the investigator or sponsor, its occurrence places the patient or subject at immediate risk of death. It does not include an AE or suspected adverse reaction that, had it occurred in a more severe form, might have caused death.

Important medical events that may not result in death, be life-threatening, or require hospitalization may be considered serious when, based upon appropriate medical judgment, they may jeopardize the patient or subject and may require medical or surgical intervention to prevent one of the outcomes listed in this definition. Examples of such medical events include allergic bronchospasm requiring intensive treatment in an emergency room or at home, blood dyscrasias or convulsions that do not result in inpatient hospitalization, or the development of drug dependency or drug abuse.

- - 1. Unexpected Adverse Event or Unexpected Suspected Adverse Reaction

An AE or suspected adverse reaction is considered “unexpected” if:

- it is not listed in the investigator brochure or
- is not listed at the specificity or severity that has been observed; or, if an investigator brochure is not required or available,
- is not consistent with the risk information described in the general investigational plan or elsewhere in the current application, as amended.

For example, under this definition, hepatic necrosis would be unexpected (by virtue of greater severity) if the investigator brochure referred only to elevated hepatic enzymes or hepatitis. Similarly, cerebral thromboembolism and cerebral vasculitis would be unexpected (by virtue of greater specificity) if the investigator brochure listed only cerebral vascular accidents. “Unexpected,” as used in this definition, also refers to adverse events or suspected adverse reactions that are mentioned in the investigator brochure as occurring with a class of drugs or as anticipated from the pharmacological properties of the drug, but are not specifically mentioned as occurring with the particular drug under investigation.

- 1. Relationship to Investigational Product

The investigator must assign a relationship of each AE to the receipt of the investigational product. The investigator will use clinical judgment in conjunction with the assessment of a plausible biologic mechanism, a temporal relationship between the onset of the event in relation to receipt of the investigational product, and identification of possible alternate etiologies including underlying disease, concurrent illness or concomitant medications. The following guidelines should be used by investigators to assess the relationship of an AE to study product administration. **ONLY A PHYSICIAN CAN MAKE THIS DETERMINATION.**

**Not related:** No relationship to investigational product. Applies to those events for which evidence exists that there is an alternate etiology.

**Unlikely:** Likely unrelated to the investigational product. Likely to be related to factors other than investigational product, but cannot be ruled out with certainty.

**Possible:** An association between the event and the administration of investigational product cannot be ruled out. There is a reasonable temporal association, but there may also be an alternative etiology such as the subject’s clinical status or underlying factors including other therapy.

**Probable:** There is a high degree of certainty that a relationship to the investigational product exists. There is a reasonable temporal association, and the event cannot be explained by known characteristics of the subject’s clinical state or factors including other therapy.

**Definite:** An association exists between the receipt of investigational product and the event. An association to other factors has been ruled out.

The categories of definite, probable, and possible will be considered investigational product related with regards to summary statistics.

- 1. Recording Adverse Events
     1. Methods/Timing for Assessing, Recording, and Analyzing Safety Endpoints

AEs and SAEs will be collected at all study visits, documented in the source records, and recorded on the CRFs using accepted medical terms and/or the diagnoses that accurately characterize the event. When a diagnosis is known, the AE term recorded on the AE CRF will be the diagnosis rather than a constellation of symptoms. The investigator will assess all AEs for seriousness, relationship to investigational product, severity, and other possible etiologies. When an event has not resolved by study closure, it will be documented on the AE CRF as “ongoing”.

The timeframe for the collection of AEs and SAEs begins at the first administration of investigational product through the end of the study.

- - 1. Duration of Follow-Up of Subjects after Adverse Events

Investigators are required to follow SAEs to resolution, even if this extends beyond the prescribed reporting period. Resolution is the return to baseline status or stabilization of the condition with the probability that it will become chronic. The SAE outcomes will be reported to the sponsor’s representative using the Serious Adverse Event Report CRF.

The investigator will monitor subjects for the occurrence of AEs from the first study drug application through nominal Day 168 and record all observed AEs in the CRF. All AEs, regardless of seriousness or relationship to the study drug are to be recorded on the CRF. Whenever possible, symptoms should be grouped as a single syndrome or diagnosis. The investigator should specify the date of onset, date of resolution, maximum severity grade, corrective therapy given (if applicable), outcome, and his/her opinion as to association between the AE and the administration of the study drug. Clinically significant AEs ongoing at nominal Day 168 will be followed to resolution or until stabilized including the administration of any concomitant medications.

Investigators are not obligated to actively seek SAEs in former subjects; however, if a SAE, considered to be related to the investigational product is brought to the attention of the investigator at any time following completion of the study, the event will be reported as defined in section 11.5.1.

- - 1. Severity Assessment

All AEs will be assessed for severity by the investigator. Inherent in this assessment is the medical and clinical consideration of all information surrounding the event including any medical intervention required. Any grade 4 (life-threatening) AE must be reported as an SAE.

The CRF for AEs will reflect only the highest severity for continuous days an event occurred.

All AEs will be graded according to the definitions provided below. Assignment of grade based on the intensity of symptoms and the degree of limitation of usual daily activities will be done according to severity using the following criteria:

**Grade 1**: Mild symptoms invoking a minimum degree of discomfort that are easily tolerated.

**Grade 2**: Moderate symptoms that result in a reduction in normal daily activity, but is not totally incapacitating. This may or may not require medical intervention.

**Grade 3**: Severe symptoms that may be totally incapacitating or result in marked reduction in normal daily activity. Medical intervention is usually required.

**Grade 4**: Potentially life-threatening event that requires emergency intervention or hospitalization.

**Application site reactions** including erythema/redness, swelling/edema, vesicles will be scored according the following criteria. If there is a change in the application site compared to baseline (ie, an increase in grade), then this will be recorded as an AE.

Each application site will be assessed for the above reactions and the highest severity grade of any application site will be recorded on the AE CRF.

**Grade 0:** No evidence of erythema/redness, swelling/edema, or vesicles.

**Grade 1:** Visibly present but not associated with any other symptoms.

**Grade 2:** Visibly present, large area around lesion site, and associated with other symptoms such as itching or pain. Medical intervention may be required.

**Grade 3:** Severe symptoms that require medical intervention.

**Pain in the lesions** prior to study drug administration (assessed before the daily lesion cleaning) will be graded according to the following criteria:

**Grade 0:** None (feels no pain)

**Grade 1:** Mild pain (does not interfere with daily activity)

**Grade 2:** Moderate pain (interferes with daily activity)

**Grade 3:** Severe pain (daily activities are interrupted)

- - 1. AE Actions and Outcomes

For each AE that is reported the actions taken with respect to study drug including: 1) none, 2) temporarily discontinued, or 3) permanently discontinued will be recorded on the AE CRF. Also, outcomes will also be recorded including: 1) resolved; 2) resolved with sequelae; 3) ongoing, and 4) unknown. If the AE required any treatment, this will also be recorded.

- 1. Reporting Adverse Events

The principal investigator will report all AEs to the sponsor’s representative (USAMRMC Division of Regulated Activities and Compliance) and the Gorgas Institutional Bioethics Committee in the appropriate safety, annual, and/or final reports. The study site will provide data files to the sponsor’s representative for preparation of annual and final reports to the FDA.

- - 1. Reporting Serious and Unexpected Adverse Events

Contact information for reporting SAEs is provided in Table 10.

- - - 1. Reporting to the Sponsor

**All SAEs and unexpected AEs** must be reported promptly (within 72 hours) to the sponsor’s representative as per 21 CFR 312.64, whether or not the event is considered related to study product. Further, the investigator should comply with relevant study site SOPs on reporting SAEs.

The minimum information that the investigator will provide to the USAMMDA Division of Regulated Activities and Compliance is specified in Table 11. The sponsor’s representative may request additional information for purposes of the study.

In order to comply with regulations mandating sponsor notification of specified SAEs to the FDA within 7 calendar days, investigators must submit additional information as soon as it is available. The sponsor’s representative will report unexpected SAEs associated with the use of the drug to the FDA as specified at 21 CFR 312.32 (c).

Investigators must follow all relevant regulatory requirements as well as specific policy at each institution regarding the timely reporting of SAEs to the local Ethics Committee, research monitor, and the USAMRMC ORP HRPO.

Reporting to the sponsor’s representative does not fulfill the investigator’s duty to report all unanticipated problems involving risk to human subjects or others to the Ethics Committee. The principal investigator will notify the local Ethics Committee, the ORP HRPO, USAMMDA CSSD, and the research monitor.

Table 10: Study Contacts for Reporting Serious Adverse Events

| **Sponsor’s Regulatory Advisor Division of Regulated Activities and Compliance** | US Army Medical Research and Materiel Command ATTN: MCMR-UMR 1430 Veterans Drive Fort Detrick, MD 21702-5009 Tel: +1-301-619-0317 Fax: +1-301-619-0197 Email: usarmy.detrick.medcom-usammda.mbx.usamrmc-regulatory-affairs@mail.mil |
| --- | --- |
| **Sponsor’s Representative Product Safety Surveillance Branch** | U.S. Army Medical Materiel Development Activity ATTN: CSSD-PSSB  1430 Veterans Drive, Fort Detrick, MD 21702-5009 Fax: 301-619-7790 Telephone: 301-619-1106  Email: usarmy.detrick.medcom-usammda.mbx.sae-reporting@mail.mil |
| **Institutional Review Board** | Institutional Bioethics Committee Gorgas Memorial Institute Avenida Justo Arosemana, Entre calles 35 y 36 Panama City, Panama Tel: +507-527-4823 |
| **USAMRMC Office of Research Protections**  **Human Research Protections Office** | USAMRMC, Office of Research Protections (ORP) Human Research Protections Office (HRPO) US Army Medical Research and Materiel Command ATTN: MCMR-RPI, 504 Scott Street Fort Detrick, Maryland 21702 Tel: +1 (301) 619-6240 Fax: +1 (301) 619-4165 Email: usarmy.detrick.medcom-usamrmc.other.irb-office@mail.mil |
| **Research Monitor** | Maria Eugenia Barnett de Antinori, MD Regional Training Center, Gorgas Memorial Institute Avenida Justo Arosemena, Entre Calles 35 y 36 Panama City, Panama Tel: +507-6615-1338  E-mail: mebantinori@cwpanama.net |

Table 11: SAE Information to be Reported to the Sponsor's Representative

| Notification Method | Information to be Provided |
| --- | --- |
| **Email or Telephone (within 72 hours)** | IND number, sponsor study number, name of the investigational product, and investigator name and contact number |
|  | Subject identification number and initials |
|  | AE, onset date, date of investigational product administration, severity, relationship, and subject’s current status |
| **AND** |  |
| **Email or Fax** | Cover sheet or letter |
|  | Adverse event case report form |
|  | Serious adverse event report form |
|  | Concomitant medication case report form or a list of concomitant medications |
|  | Medical record progress notes including pertinent laboratory/diagnostic test results |
| NOTE: When submitting SAE reports via email, the subject line of each email notification will read as follows:  **SAFETY REPORT – IND # _____, Sponsor Study #_____, Subject# _____, Event term: _____** | |

- - - 1. Reporting to the Ethics Committee

Unanticipated problems involving risk to subjects or others (including study staff), SAEs related to participation in the study and all subject deaths should be promptly reported by phone, email, or fax to the Gorgas Institutional Bioethics Committee.

Investigators are required to forward safety information provided by the sponsor’s representative to the Gorgas Institutional Bioethics Committee.

- - - 1. Reporting to ORP HRPO

Unanticipated problems involving risk to subjects or others, SAEs related to participation in the study, and all subject deaths related to participation in the study should be promptly reported by telephone, email, or fax to the USAMRMC ORP HRPO. A complete written report should follow the initial notification.

- - 1. Reporting Additional Immediately Reportable Events to the Sponsor’s Representative and ORP HRPO
       1. Pregnancy

Each pregnancy must be reported **immediately (within 72 hours of identification)** by email or fax to the sponsor’s representative and the ORP HRPO.

Subjects who become pregnant after Day 1 until 1 week after completion of study cream application will be followed to term, and the following information will be gathered for outcome: date of delivery, health status of the mother and child including the child’s gender, height and weight. Complications and or abnormalities should be reported including any premature terminations. A pregnancy is reported as an AE or SAE only when there is suspicion that the investigational product may have interfered with the effectiveness of contraception or there was a serious complication in the pregnancy including a spontaneous abortion or an elective termination for medical rationale.

- - - 1. AE-related Withdrawal of Consent

Any AE-related withdrawal of consent during the study must be reported **immediately (within 72 hours of identification)** by email or fax to the sponsor’s representative and the ORP HRPO.

- - - 1. Pending Inspections/Issuance of Reports

The knowledge of any pending compliance inspection/visit by the FDA, Office for Human Research Protections (Department of Health and Human Services), or other government agency concerning clinical investigation or research, the issuance of Inspection Reports, FDA Form 483, warning letters, or actions taken by any Regulatory Agencies including legal or medical actions and any instances of serious or continuing noncompliance with the regulations or requirements will be reported immediately to USAMMDA ORP HRPO and the sponsor’s representative.

- - 1. IND Annual Report to the FDA

The principal investigator will be responsible for the preparation of a detailed annual synopsis of clinical activity, including AEs, for submission to the sponsor’s representative. Each annual report will summarize IND activity for 1 year beginning approximately 3 months before the IND FDA anniversary date. The sponsor’s representative will notify the principal investigator of the due date with sufficient time for the principal investigator to assemble the required information.

- - 1. Final Report

A final study report will be prepared in accordance with “Guidance for Industry: Submission of Abbreviated Reports and Synopses in Support of Marketing Applications” and ICH E3 Guideline “Structure and Content of Clinical Study Reports” and provided to the sponsor’s representative for review and approval. The sponsor’s representative will use this report to prepare the final clinical study report for submission to the FDA.

The PI will report all AEs to the sponsor’s representative (USAMMDA Division of Regulated Activities and Compliance) and the Ethics Committee in the appropriate safety, annual, and/or final reports. The study site will provide CRFs to the Data Management Center that will provide Statistical Analysis System (SAS) data sets transfer files to the sponsor’s representative for preparation of annual and final reports to the FDA.

1. Statistics

Detailed statistical procedures, listings, table shells and figures will be provided in a separate statistical analysis plan (SAP) written shortly after protocol approval but before any subject enrollment. The SAP will be finalized before study close-out and database lock.

Descriptive statistics will be used to present study data. Continuous variables will be presented as number of observations (n), mean, standard deviation (SD), median, minimum and maximum values. Categorical variables will be presented as counts and percentages. All data will be presented separately by treatment group. Both the primary and secondary efficacy endpoints will be presented for the modified intention-to-treat (mITT) and evaluable analysis sets. SAS version 9.2 will be used to perform statistical analyses. All statistical tests will be two-sided. P-values < 0.05 will be considered statistically significant.

- 1. Description of Statistical Methods
     1. Analysis Addressing the Primary Study Efficacy Objective

Final clinical cure rates of the index lesion will be compared between the two treatment groups by uncorrected chi-square test. The difference in cure rates and 95% CI for the difference will also be presented. Subjects with missing data, making it impossible to determine if they can be counted as clinical cures, will be considered failures in these analyses.

- - 1. Analysis Addressing the Secondary Study Objectives

No adjustments will be made to correct for multiplicity of comparisons of secondary efficacy endpoints.

- - - 1. Percentage of Subjects with All Lesions Cured

Final clinical cure rates of all lesions in a subject will be compared between the two treatment groups by uncorrected chi-square test. The difference in cure rates and 95% CI for the difference will also be presented. Subjects with missing data, making it impossible to determine if they can be counted as clinical cures, will be considered failures in these analyses.

- - - 1. Percentage of All Lesions Cured

Final clinical cure rates of all lesions treated (ignoring subject) will be compared between the two treatment groups by uncorrected chi-square test. The difference in cure rates and 95% CI for the difference will also be presented. Subjects with missing data, making it impossible to determine if they can be counted as clinical cures, will be considered failures in these analyses.

- - - 1. Lesion Area Measurements

Summary statistics for area measurements including total area of ulceration of the index lesion and all ulcerated lesions by subject will be presented both in tabular form and graphically. Areas over time will be compared between the two treatment groups by mixed effects models using an appropriate transformation to normalize the data.

- - - 1. Ulcerated Lesion Cure Rate

The percentage of subjects at each measurement time point with complete cure of the index lesion and all ulcerated lesions will be presented. A complete cure of an ulcerated lesion is defined as 100% re-epithelialization of the lesion (a measurement of ulceration of 0 x 0 mm). The percentages of subjects with 100% re-epithelialization of the index lesion and all ulcerated lesions over time will be compared between treatment groups using a mixed effects model with treatment and time in the model. If data are not normally distributed appropriate transformations will be performed.

- - - 1. Time to Initial Clinical Cure

Median time to initial clinical cure will be determined using time to events methods. Time to initial clinical cure will be compared between the two groups using log-rank test. Missing data will be censored for this analysis. It should be noted that with the limited number of time points for cure to be assessed, that this analysis may not be very sensitive to differences.

- - - 1. New Lesions Incidence

The number of new lesions (if any) that develop during the study and the cure rates (100% re‑epithelialization of ulcerated lesions or resolution of non-ulcerated lesions) will also be presented in a summary table.

- - - 1. Subset Analysis by Infecting Leishmania Species

The proportions and 95% CI of the difference in proportions of subjects with final clinical cure of the index lesion and all lesions by infecting *Leishmania* species will be presented.

- - 1. Safety Analyses
       1. Demographics and Treatment Compliance

Summaries of the subject demographics including age, gender, ethnicity and race, number of baseline lesions, lesion areas, and length of time between initial presence of current lesions and treatment for the mITT and evaluable populations will be provided. Compliance with scheduled treatments will also be summarized, including total exposure (number of days and total dose) to study drug. Protocol compliance will be presented as percentages of subjects attending scheduled visits over the duration of the study.

By-subject listings will be provided for demographic data, medical history, physical examination results, baseline vital signs, clinical laboratory measurements, clinical response measurements, AEs, concomitant medication use, SAEs, discontinuation due to AEs, and other significant AEs.

AEs will be coded using the most recent version of Medical Dictionary of Regulatory Activities (MedDRA) by assigning a preferred term and will be grouped by system, organ, and class (SOC) designation. The severity, frequency, and relationship of AEs to study drug will be presented by preferred term by SOC grouping. Listings of each individual AE including start date, stop date, severity, relationship, outcome, and duration will be provided. Laboratory data and vital signs will be presented as summary statistics by Study Day including changes from baseline as well as in by-subject data listings.

Blood creatinine, aspartate aminotransferase (AST), and alanine aminotransferase (ALT) levels will be presented as summary statistics of serum concentrations at baseline and Day 20 and change from baseline. A summary and listing of analyte concentrations for subjects with levels outside normal laboratory limits will also be presented.

Vital signs including blood pressure, heart rate, and temperature will be presented as summary statistics for values at baseline.

Drug exposure data will be presented as estimates using the following data: 1) amount of weight in gram of cream expelled from tubes for application; and, 2) number of days of application.

- 1. Planned Enrollment and Reason for Sample Size

The original sample size for this study was planned at 300 randomized subjects. This was based on the previous Phase 2 study in Panama of similar design and entry criteria, in which 13 of 15(86.7%) of the subjects treated with WR 279,396 had final clinical cure of the index lesion and 9 of 15 (60.0%) of subjects treated with Paromomycin Alone had final clinical cure of the index lesion. Final cure rates for subjects based on all lesions cured were 13 of 15 (86.7%) for WR 279,396 and 8 of 15 (53.3%) for Paromomycin Alone. Based on the endpoints in this study, sample sizes were estimated for effect sizes of 5%, 10%, 15%, and 20% differences in the final cure rates in the two groups as shown in Table 12. As the difference in cure rates between the two groups in the Phase 2 study was 26.7%, and recognizing the small size of this study and the inherent error based on the small sample size, a total of 150 subjects per treatment group were selected..

Table 12: Sample Size Estimates

| Estimated % Final Clinical Cure Rate for the WR 279,396 Group | Estimated % Final Clinical Cure Rate for the Paromomycin Alone Group | % Difference between groups | n |
| --- | --- | --- | --- |
| 80 | 75 | 5 | 1094 |
| 80 | 70 | 10 | 294 |
| 80 | 65 | 15 | 138 |
| 80 | 60 | 20 | 82 |

a Sample size per group based on uncorrected chi-square test estimated n with 80% power two-sided alpha = 0.05 performed with nQuery version 6.02.

During the conduct of the study, new *Leishmania* species were identified in Panama that were not previously found during the conduct of the Phase 2 study. Other species include *Leishmania guyanensis* (*L guyanensis*) and *Leishmania braziliensis* (*L braziliensis*) in addition to *L panamensis*. Of 149 subjects randomized to the study for which speciation data were available (treatment groups blinded and combined) 74% of subjects had *L panamensis* and the others were split between *L guyanensis* (19%) and *L braziliensis* (6%). Since only *L panamensis* was identified in the Phase 2 study, and responses to treatments for Leishmaniasis can vary by species, then the effect of the species on the observable cure rates is unknown. Conservatively assuming the cure rates in the other species is 60% for WR 279,396 and is 50% in the Paromomycin Alone group, then the observable cure rate becomes 75% in WR 279,396 [(0.75 x 80%) + (0.25 x 60%)] and 61.25% in Paromomycin Alone [(0.75 x 65%) + (0.25 x 50%)]. A sample size of 200 subjects per arm will provide at least 80% statistical power to detect the specified difference in cure rates between WR 279,396 and Paromomycin Alone. Therefore, the total sample size was adjusted to 400 subjects.

- 1. Interim Analysis

Not applicable.

- 1. Accounting for Missing, Unused, and Spurious Data

Subjects who do not provide lesion response measurements at nominal Days 63 and 168 will be considered clinical failures in the mITT analysis, unless the subject has documented clinical cure of the index lesion before Day 63 and does not relapse by Day 168 (ie, there must be an assessment at nominal Day 168 but nominal Day 63 can be missing). If a subject misses the nominal Day 63 lesion assessment but had 100% re-epithelialization of all lesions before this visit and continues to have 100% re-epithelialization of all lesions by nominal Day 168, s/he will be considered a final clinical cure without relapse. Missing lesion measurements will be ignored when presented as summary statistics of lesion areas. Missing data in the time to clinical response assessments will be censored at the last available data point.

- 1. Procedures for Reporting Deviations from the Original Statistical Plan

Any deviation(s) from the original statistical plan as indicated in the protocol will be described in an amendment to the protocol and the SAP.

- 1. Selection of Subjects to be Included in Analyses

**Modified Intention-to-treat (mITT) Subjects:** The mITT analysis will include all subjects who received any administration of investigational product.

**Evaluable Group:** The evaluable subset will include all subjects who received daily doses of investigational product for at least 18 of the total 20 days and had lesion measurements at Day 63 and 168.

**Safety Group:** The safety population includes all subjects who received any administration of investigational product (ie, same as mITT).

1. Direct Access to Source Data/Documents

Subjects will be identified on CRFs by a unique subject identification code. No personal identifier will be used in any publication or communication used to support this research study. The subject identification code will be used if it becomes necessary to identify data specific to a single subject. Representatives of USAMMDA, the sponsor’s representative, the Gorgas Institutional Bioethics Committee, and the FDA are eligible to review medical and research records related to this study as a part of their responsibility to protect human subjects in clinical research. Personal identifiers will be removed from photocopied medical and research records.

- 1. Study Monitoring

Study monitoring will be the responsibility of the Fast-Track Drugs & Biologics, LLC and overseen by the USAMMDA CSSD. Upon successful approval of the protocol and establishment of the Regulatory File, the clinical monitor will establish a clinical monitoring plan. To ensure that the investigator and the study staff understand and accept their defined responsibilities, the clinical monitor will maintain regular correspondence with the site and may be present during the course of the study to verify the acceptability of the facilities, compliance with the investigational plan and relevant regulations, and the maintenance of complete records.

Monitoring visits by a designated clinical monitor will be scheduled to take place at the initiation of the study, during the study at appropriate intervals, and after the last subject has completed the study. A report of monitoring observations will be provided to the principal investigator (for corrective actions), USAMMDA Division of Regulated Activities and Compliance, and the product manager.

- 1. Audits and Inspections

Authorized representatives of the sponsor, the FDA, the Gorgas Institutional Bioethics Committee may visit the site to perform audits or inspections, including source data verification. The purpose of the audit or inspection is to systematically and independently examine all study‑related activities and documents to determine whether these activities were conducted, and data were recorded, analyzed, and accurately reported according to the protocol, GCP guideline of the ICH, and any applicable regulatory requirements.

The investigator should contact the sponsor’s representative and ORP HRPO immediately if contacted by a regulatory agency about an inspection.

- 1. Ethics Committee

The principal investigator must obtain Gorgas Institutional Bioethics Committee approval for the investigation. Initial Ethics Committee approval, and all materials approved by the Ethics Committee for this study including the subject consent forms and recruitment materials must be maintained by the investigator and made available for inspection.

The principal investigator will be responsible for preparing and submitting continuing review reports per institution and Gorgas Institutional Bioethics Committee policies. The principal investigator or a designee will submit the approved continuing review reports and the Gorgas Institutional Bioethics Committee approval notifications to HRPO as soon as the documents are available.

The principal investigator or a designee will transmit the approved final study report and the Gorgas Institutional Bioethics Committee approval notification to the USAMRMC ORP HRPO as soon as the documents are available.

1. Quality Control and Quality Assurance

To ensure compliance with GCP and all applicable regulatory requirements, the sponsor’s representative may conduct quality assurance audits. Refer to section 13.2 for more details regarding the audit process.

Auditing of the clinical trial may be conducted at any time during the study to ensure continued compliance with regulations, policies and procedures. Auditing will be undertaken, as needed, by independent personnel designated by the Quality Office, USAMMDA. Audit findings will be documented in a formal audit report that will detail the conduct of the audit and summarize the observations noted.

1. Ethics
   1. Ethics Review

The study is based on suitably performed laboratory and animal experimentation; the study will be conducted under a protocol reviewed by the Gorgas Institutional Bioethics Committee the study is to be conducted by scientifically and medically qualified persons; the benefits of the study are in proportion to the risks; the rights and welfare of the subjects will be respected; the physicians conducting the study will ensure that the hazards do not outweigh the potential benefits; the results to be reported will be accurate; subjects will give their informed consent and will be competent to do so and not under duress; and all study staff will comply with the ethical principles in 21 CFR Part 50 and the Belmont Principles.

- - 1. Review/Approval of Study Protocol

Before a clinical study can be initiated, the study protocol and other required documents will be submitted to the following departments in the order listed for review and/or approval, with the final review by the FDA:

- Integrated Product Team
- Independent Scientific Review Committee
- Sponsor’s Representative Team (Division of Regulated Activities and Compliance, USAMMDA)
- Gorgas Institutional Bioethics Committee
- National Health Authorities in the country of the clinical site
- Commander
- Office of Research Protections, Human Research Protection Office (ORP HRPO)
- Sponsor’s Representative (acting for The Surgeon General of the Army)
- USAMRMC Commanding General

Enrollment in this protocol may not begin until all approvals have been obtained and the formal authorization letter is received by the principal investigator from the sponsor’s representative.

- - 1. Protocol Modifications

All modifications to the protocol and supporting documents (informed consent, recruitment materials, etc) must be reviewed and approved prior to implementation. Any protocol amendment will be agreed upon and approved by the sponsor’s representative prior to submission to the Gorgas Institutional Bioethics Committee and prior to implementation of said change or modification. The informed consent document must be revised to concur with any amendment as appropriate and must also be reviewed and approved with the amendment. Any subject already enrolled in the study will be informed about the revision and asked to sign the revised informed consent document if the modification directly affects the individual’s participation in the study. A copy of the revised, signed, and dated informed consent document will be offered to the subject. All original versions of the informed consent document will be retained in the subject’s source documents.

All major modifications and any modification that could potentially increase risk to subjects must be submitted to the HRPO for approval prior to implementation. Documentation that the Gorgas Institutional Bioethics Committee reviewed and approved the modifications also will be submitted. All other amendments will also be submitted to the HRPO for inclusion in the HRPO study file.

- - 1. Protocol Deviation Procedures

All deviations from the protocol (eg, failure to return for follow-up visits within the time indicated in the protocol) are to be documented. The principal investigator or designee will be responsible for identifying and reporting all deviations, which are defined as isolated occurrences involving a procedure that did not follow the study protocol or study-specific procedure. Deviations will be reported annually in the continuing review report to the Gorgas Institutional Bioethics Committee and HRPO and in the final study report. Action taken in response to the deviation, and the impact of the deviation will be assessed by the principal investigator or subinvestigator and recorded as significant or non-significant.

If a protocol deviation jeopardizes the safety or rights of a subject or scientific integrity of the study, the deviation will be reported immediately to the sponsor’s representative, Gorgas Institutional Bioethics Committee, and the ORP HRPO.

- 1. Ethical Conduct of the Study

This study will be conducted in accordance with all applicable local, US Federal and DoD human research protections requirements and the Belmont Principles of respect for persons, beneficence, and justice.

The procedures set out in this study are designed to ensure that the sponsor’s representative and all study personnel abide by the principles of the ICH GCP Guideline and the CFR. The principal investigator confirms this by signing this study protocol and Form FDA 1572.

- - 1. Confidentiality

In this research, the subject’s health information will be collected and used to conduct the study; to monitor the subject’s health status; to measure effects of the investigational product; to determine research results, and possibly to develop new tests, procedures, and commercial products. Health information is used to report results of research to the sponsor’s representative and US Federal regulators and may be reviewed during study audits for compliance with study plans, regulations, and research policies. After the study ends, each subject has the right to see and receive a copy of his/her information.

Representatives of the The Surgeon General as the sponsor, USAMMDA as the sponsor’s representative, HQ USAMRMC IRB, the Gorgas Institutional Bioethics Committee, the DoD, and the FDA are eligible to photocopy and review records related to this protocol as a part of their responsibility to protect the participants of this treatment protocol. No personal identifier will be used in any publication or communication used to support this research study. The subject’s identification number will be used in the event it becomes necessary to identify data specific to a single subject.

- - 1. Compensation for Participation

Subjects will be compensated for any loss of days’ work or transportation costs caused by their participation in the study as recommended by the Gorgas Institutional Bioethics Committee.

- 1. Written Informed Consent

The informed consent process and document will be reviewed and approved by the Gorgas Institutional Bioethics Committee and sponsor’s representative prior to initiation of the study. The consent document contains a full explanation of the possible risks, advantages, and alternate treatment options, and availability of treatment in the case of injury, in accordance with 21 CFR 50. The consent document indicates that by signature, the subject permits access to relevant medical records by the sponsor’s representative and by representatives of the FDA. The sponsor’s representative will submit a copy of the initial Ethics Committee - and sponsor’s representative-approved consent form to the FDA and will maintain copies of revised consent documents that have been reviewed and approved by the Gorgas Institutional Bioethics Committee.

A written informed consent document, in compliance with local regulations, 21 CFR Part 50, 32 CFR Part 219, DoDI 3016.02 and the Belmont Principles, will be signed by the subject before any study-related procedures are initiated for each subject. Separate consents for adult study participants and parents/legal guardians of children ages 2 to 17 years will be used. In addition, an assent form will also be used for children ages 7 to 17. Verbal assent will be obtained from children ages 2 to 6 years. Written assent is waived for children ages 2 to 4; however, consent of the parents/legal guardians of these children will be required for their participation. The consent/assent documents must be retained by the investigator as part of the study records. The investigators or their designees will present the protocol in lay terms to individual subjects. Questions on the purpose of the protocol, protocol procedures, and risks to the subjects will then be solicited. Any question that cannot be answered by the staff will be referred to the principal investigator. No subject should grant consent/assent until questions have been answered to his/her satisfaction. The subject should understand that the investigational product is an investigational drug and is not licensed by the FDA for commercial use, but is permitted to be used in this clinical research. Informed consent/assent includes the principle that it is critical the subject be informed about the principal potential risks and benefits. This information will allow the subject to make a personal risk versus benefit decision and understand the following general principles:

- Participation is entirely voluntary,
- Subjects may withdraw from participation at any time,
- Refusal to participate involves no penalty,
- The individual is free to ask any questions that will allow him/her to understand the nature of the protocol; and
- A description of this clinical trial will be available on http://www.ClinicalTrials.gov, as required by US law.

If the candidate desires to participate in the study, s/he will be asked to sign the Informed Consent or Assent, as appropriate. No study procedure will be performed prior to obtaining Informed Consent/Assent. Subjects who refuse to participate or who withdraw from the study will be treated without prejudice. All study subjects will be offered a copy of the signed Informed Consent/Assent.

Should the protocol be modified, the subject consent document will be revised as appropriate to reflect the changes to the protocol. If a previously enrolled subject is directly affected by the change, the subject will be informed of the changes and given a copy of the revised informed consent/assent document to review. The subject will then be asked to sign and date the revised informed consent/assent, and a copy of the signed consent/assent will be provided to the subject or legal guardian.

1. Data Handling and Recordkeeping

Source documents will be maintained for each subject who consents to the study. The source documents will be retained at the site.

Data will be collected at the study site on source documents, which will be transcribed at the site into CRFs. The CRFs will be supplied by Fast-Track Drugs and Biologics, LLC (Fast-Track). CRFs are intended to be completed on an ongoing basis during the study. The source documents are the original records for verification of data. CRFs should be completed according to the instructions in the CRF Completion Instructions Manual. Completed CRFs will be collected after monitoring and submitted to Fast-Track. The Investigator is responsible for maintaining accurate, complete and up-to-date records for each subject. The Investigator is also responsible for maintaining any source documentation related to the study, including lesion digital pictures, clinical laboratory data, informed consent forms, and drug accountability records.

A detailed data management plan will be written and approved by the Integrated Product Team, sponsor’s representative, and the principal investigator. The plan will be drafted prior to study initiation but will be finalized before study close-out and database lock.

All research data will be entered, using a double-data entry procedure, into a computerized database, designed in accordance with 21 CFR Part 11 and based on protocol requirements defined by the sponsor’s representative in association with the principal investigator and clinical data manager. Monitored data received at Fast-Track will be reviewed and verified prior to being entered into the main study database. If incomplete or inaccurate data are found, a data clarification request will be forwarded to the clinical site for a response. The site will resolve data inconsistencies and errors prior to returning corrected CRFs to Fast-Track. All corrections and changes to the data will be reviewed prior to being entered into the main study database.

- 1. Inspection of Records

The sponsor’s representative or designee will be allowed to visit the investigation facilities for the purpose of monitoring any aspect of the study. The investigator agrees to allow the monitor to inspect the drug storage area, investigational product stocks, drug accountability records, subject charts, study source documents, and other records relative to study conduct.

Subjects’ health information is used to report results of research to the sponsor’s representative and Federal regulators and may be reviewed during study audits for compliance with study plans, regulations, and research policies. The consent document indicates that by signature, the subject permits access to relevant medical records by the sponsor’s representative and by representatives of the FDA.

Upon a subject’s termination from the trial, completed CRFs will be ready and available for on-site review by the sponsor’s representative at scheduled monitoring visits.

- 1. Retention of Records

The principal investigator must maintain all documentation relating to the study for a period of at least 2 years after the last marketing application approval, or if not approved for 2 years following the discontinuance of the investigational product for investigation. If it becomes necessary for the sponsor’s representative or designee or the FDA to review any documentation relating to the study, the investigator must permit access to such records.

Completed, monitored CRFs will be stored in a secure location by the sponsor’s representative or designee. A copy of each completed CRF will be retained by the investigator.

The principal investigator will be responsible for retaining sufficient information about each subject, ie, name, address, telephone number, and subject identifier in the study, so that the sponsor’s representative, the Gorgas Institutional Bioethics Committee, the FDA, employees of USAMRMC, or other regulatory authorities may have access to this information should the need arise.

It is the policy of the USAMRMC that data sheets are to be completed for all subjects participating in research. The data sheets will be entered into this Command’s Volunteer Registry Database. The information to be entered into this confidential data base includes the subject’s name, address, study title, and dates of participation. The intent of this data base is twofold: first, to readily answer questions concerning an individual’s participation in research sponsored by USAMRMC; and second, to ensure that USAMRMC can exercise its obligation to ensure research subjects are adequately warned (duty to warn) of risks and to provide new information as it becomes available. The information will be stored at USAMRMC for a minimum of 75 years. The Volunteer Registry Database is a separate entity and is not linked to the study database.

1. Publication Policy

All data collected during this study will be used to support IND 50098. All data may be published in the open medical or military literature with the identity of the subjects protected. Anyone desiring to publish or present data obtained during the conduct of the study will conform to Instituto Conmemorativo Gorgas de Estudios de la Salud policies and then forward the publication for review to the Commander, USAMMDA or designee and usarmy.detrick.medcom-usamrmc.list.clearances@mail.milprior to submission.

All data to be published must be in agreement with the data analyzed from the locked database for submission to the FDA.

1. List of References

Alrajhi AA, Ibrahim EA, De Vol EB, Khairat M, Faris RM, Maguire JH. Fluconazole for the treatment of cutaneous leishmaniasis caused by *Leishmania major*. N Engl J Med. 2002;346:891‑5.

Andrade-Narvaez FJ, Vargas-Gonzalez A, Canto-Lara SB, Damian-Centeno AG. Clinical picture of cutaneous leishmaniases due to *Leishmania (Leishmania) mexicana* in the Yucatan peninsula, Mexico. Mem Inst Oswaldo Cruz. 2001;96:163-7.

Arevalo J, Ramirez L, Adaui V, et al. Influence of *Leishmania (Viannia)* species on the response to antimonial treatment in patients with American tegumentary leishmaniasis. J Infect Dis. 2007;195:1846-51.

Armed Forces Health Surveillance Center. Leishmaniasis (ICD-9: 085.0 to 085.9). Deployment‑related conditions of special surveillance interest, U.S. Armed Forces, by months and service, January 2003-January 2001. Medical Surveillance Monthly Report (MSMR). 2011 Feb; 18(2):15.

Aronson NE, Sanders JW, Moran KA. In harm’s way: Infections in deployed American military forces. Clin Infect Dis. 2006;45:1045-51.

Asilian A, Jalayer T, Nilforooshzadeh M, Ghassemi RL, Peto R, Wayling S, Olliaro P, Modabber F. Treatment of cutaneous leishmaniasis with aminosidine (paromomycin) ointment: double-blind, randomized trial in the Islamic Republic of Iran. Bull World Health Organ. 2003;81:353-9.

Asilian A, Jalayer T, Whitworth JA, Ghasemi RL, Nilforooshzadeh M, Olliaro P. A randomized, placebo-controlled trial of a two-week regimen of aminosidine (paromomycin) ointment for treatment of cutaneous leishmaniasis in Iran. Am J Trop Med Hyg. 1995;53:648-51.

Bellazoug S, Neal RA. Failure of meglumine antimonate to cure lesions due to *L major* in Algeria. Trans R Soc Trop Med Hyg. 1986;80:670.

Berman JD. Chemotherapy for leishmaniasis: biochemical mechanisms, clinical efficacy, and future strategies. Rev Infect Dis. 1988;10:560-86.

Byrnes KP (General). Memorandum for General Richard A. Cody, Vice Chief of Staff, U.S. Army, 201 Army Pentagon, Washington, D.C. December 10, 2004.

Carreira PF, Maingon R, Ward RD, Noyes H, Ponce C, Belli A, Arana B, Zeledon R, Sousa OE. Molecular techniques in the characterization of Leishmania isolates from Central America. Ann Trop Med Parasitol. 1995;89 Suppl 1:31-36.

Carter KC, Alexander J, Baillie AJ. Studies on the topical treatment of experimental cutaneous leishmaniasis: the therapeutic effect of methyl benzethonium chloride and the aminoglycosides, gentamicin and paromomycin. Ann Trop Med Parasitol. 1989;83:233-9.

Choi C, Lerner EA. Leishmaniasis as an emerging infection. J Investig Dermatol Symp Proc. 2001;6:175-82.

Christensen HA VA, Petersen JL. Short report: Epidemiologic studies on cutaneous leishmaniasis in eastern Panama. Am J Trop Med Hyg. 1999;60:54-57.

Daneshvar H, Coombs GH, Hagan P, Phillips RS. Leishmania mexicana and Leishmania major: attenuation of wild-type parasites and vaccination with the attenuated lines. J Infect Dis. 2003;187:1662-8.

Daneshvar H, Burchmore R, Hagan P, Phillips RS. Leishmania major H-line attenuated under pressure of gentamicin, induces a Th1 response which protects susceptible BALB/c mice against infection with virulent *L. major*. Parasitology. 2009;136:1243-50.

Daneshvar H, Molaei MM, Kamiabi H, Burchmore R, Hagan P, Stephen Phillips R. Gentamicin-attenuated Leishmania infantum: cellular immunity production and protection of dogs against experimental canine leishmaniasis. Parasite Immunol. 2010;32:722-30.

Davies C, Reithinger R, Campbell-Lendrum D, Feliciangeli D, Borges R, Rodriguez N. The epidemiology and control of leishmaniasis in Andean countries. Cad Saude Publica. 2000;16:925-50.

Edrissian G, Mohammadi M, Kanani A, Afshar A, Hafezi R, Ghorbani M, Gharagozloo AR. . Bacterial infections in suspected cutaneous leishmaniasis lesions. Bull World Health Organ. 1990;68:473-7.

Fontes CO, Carvalho, MA, Nicoli JR, Hamdan JS, Mayrink W, Genaro O, Carmo LS, Farias LM. Identification and antimicrobial susceptibility of micro-organisms recovered from cutaneous lesions of human American tegumentary leishmaniasis in Minas Gerais, Brazil. J Med Microbiol. 2005;54:1071-6.

Glaza S. Study Report: Photoallergic contact dermatitis study of WR 279,396, an antileishmanial topical cream in guinea pigs (Armstrong Method). September 30, 1994a.

Glaza SM. Study Report: Dermal sensitization study of WR 278,396, an antileishmanial topical cream in guinea pigs – closed patch technique. September 30, 1994b.

Glaza S. Study Report: Primary dermal photoactivated irritation study of WR 279,396, an antileishmanial topical cream in rabbits. Hazleton Wisconsin, Inc, 1995. Laboratory Project Identification No. HWI 40501110. 1995.

Goncalves GS, Fernandes AP, Souza RC, Cardoso JE, de Oliveira-Silva F, Maciel FC, Rabello A, Ferreira LA. Activity of a paromomycin hydrophilic formulation for topical treatment of infections by *Leishmania (Leishmania) amazonensis* and *Leishmania (Viannia) braziliensis*. Acta Trop. 2005;93:161-7.

Gontijo B, de Carvalho ML. [American cutaneous leishmaniasis]. Rev Soc Bras Med Trop. 2003;36:71-80.

Grogl M, Schuster BG, Ellis WY, Berman JD. Successful topical treatment of murine cutaneous leishmaniasis with a combination of paromomycin (Aminosidine) and gentamicin. J Parasitol. 1999;85:354-9.

Hepburn NC. Cutaneous leishmaniasis: an overview. J Postgrad Med. 2003;49:50-4.

Herwaldt BL, Berman JD. Recommendations for treating leishmaniasis with sodium stibogluconate (Pentostam) and review of pertinent clinical studies. Am J Trop Med Hyg. 1992;46:296-306.

Isaac-Marquez AP, Lezama-Davila CM. Detection of pathogenic bacteria in skin lesions of patients with chiclero's ulcer. Reluctant response to antimonial treatment. Mem Inst Oswaldo Cruz. 2003;98:1093-5.

Lesho EP, Wortmann G, Neafie R, Aronson N. Nonhealing skin lesions in a sailor and a journalist returning from Iraq. Cleve Clin J Med. 2005;72:93-4, 96, 98-9 passim.

Levine BS WC. Four week dermal toxicity study of WR 279,396 in CD® rats. USAMRMC Contract No. DAMD17-92-C-2001, UIC/TRL Study No. 176. 1995.

Miranda A, Carrasco R, Paz H, Pascale JM, Samudio F, Saldana A, Santamaria G, Mendoza Y, Calzada JE. Molecular epidemiology of American tegumentary leishmaniasis in Panama. Am J Trop Med Hyg. 2009;81:565-71.

Murray HW, Berman JD, Davies CR, Saravia NG. Advances in leishmaniasis. Lancet. 2005;366:1561-77.

Navin TR, Arana BA, Arana FE, Berman JD, Chajon JF. Placebo-controlled clinical trial of sodium stibogluconate (Pentostam) versus ketoconazole for treating cutaneous leishmaniasis in Guatemala. J Infect Dis. 1992;165:528-34.

Neal RA, Allen S, McCoy N, Olliaro P, Croft SL. The sensitivity of *Leishmania* species to aminosidine. J Antimicrob Chemother. 1995;35:577-84.

Netto EM, Marsden, P.D., Llanos-Cuentas EA, Costa JM, Cuba CC, Barreto AC, Badaró R, Johnson WD, Jones TC. . Long-term follow-up of patients with *Leishmania (Viannia) braziliensis* infection and treated with Glucantime. Trans R Soc Trop Med Hyg. 1990;84:367-70.

Organizacion Panamericana de la Salud (OPS). Salud en las Americas 2007. Publicacion Cientifica y Tecnica No. 622. Vol. II (Paises). Washington, DC: 2007.

Pearson R. Agents Active against Parasites and Pneuocystis carinii. In Mandell: Principles and Practice of Infectious Diseases, 5th edition. 2000. Churchill Livingston, Inc 2000.

Ramírez JL, Guevara, P. Persistent infections by *Leishmania (Viannia) braziliensis*. Mem Inst Oswaldo Cruz. 1997;92:333-8.

Reithinger R, Dujardin JC, Louzir H, Pirmez C, Alexander B, Brooker S. Cutaneous leishmaniasis. Lancet Infect Dis. 2007;7:581-96.

Republica de Panamá, Ministerio de Salud, Dirección General de Salud Pública, Departamento de Epidemilogía, Cuadro Comparativo de Casos de Leishmaniasis 2000-2007.

Rodrigues A, Hueb M, Santos TA, Fontes CJ. Factors associated with treatment failure of cutaneous leishmaniasis with meglumine antimoniate. Rev Soc Bras Med Trop. 2006;39:139-45.

Romero G, Guerra, MV, Paes MG, Macêdo VO. Comparison of cutaneous leishmaniasis due to Leishmania (Viannia) braziliensis and L. (V.) guyanensis in Brazil: therapeutic response to meglumine antimoniate. Am J Trop Med Hyg. 2001;65:456-65.

Rotreau B, Joubert M, Clyti E, et al. Leishmaniasis among gold miners, French Guiana. Emerging Infectious Diseases. 2006;12:1169-1170.

Saenz-Anduaga E, Chavez-Mancilla, M. Leishmaniasis en el Hospital Militar Central: studio clinico epidemiologico. Dermatologia Peruana. 2004:4:110-120.

Saldanha A, Romero, GAS, Merchan-Hamann E, Magalhaes AV, Macedo VO. Comparative study between sodium stibogluconate BP 88® and meglumine antimoniate for cutaneous leishmaniasis treatment: Efficacy and safety. Revista da Sociedade Brasileira de Medicina Tropical. 1999;32:383-387.

Schubach A, Marzochi MC, Cuzzi-Maya T, Oliveira AV, Araujo ML, Oliveira AL, Pacheco RS, Momen H, Conceicao-Silva F, Coutinho SG, Marzochi KB. Cutaneous scars in American tegumentary leishmaniasis patients: a site of Leishmania (Viannia) braziliensis persistence and viability eleven years after antimonial therapy and clinical cure. Am J Trop Med Hyg. 1998;58:824-7.

Soto J, Grogl M, Berman J, Olliaro P. Limited efficacy of injectable aminosidine as single-agent therapy for Colombian cutaneous leishmaniasis. Trans R Soc Trop Med Hyg. 1994;88:695-8.

Soto J, Soto P. [Current situation and future of antileishmanial therapy in Colombia]. Biomedica 2006;26 Suppl 1:194-206.

Soto-Mancipe J, Grogl M, Berman JD. Evaluation of pentamidine for the treatment of cutaneous leishmaniasis in Colombia. Clin Infect Dis. 1993;16:417-25.

Vásquez A, Paz H, Alvar J, Perez D, Hernandez C, 1998. Informe final: Estudios sobre la epidemiología de la leishmaniasis en la parte occidental de la República de Panamá. Panamá: Instituto Conmemorativo Gorgas de Estudio de la Salud, MINSA.

WHO. Leishmaniasis Fact Sheet. Updated January 2014. http://www.who.int/leishmaniasis/en/ (Accessed 4June2014).

Zakraoui H, Ben Salah A, Ftaiti A, Marrakchi H, Zaatour A, Zaafouri B, Ahmadi Z, Garraoui A, Ben Osman A, Dellagi K, et al. [Spontaneous course of lesions of *Leishmania major* cutaneous leishmaniasis in Tunisia]. Ann Dermatol Venereol. 1995;122:405-7.

1. Study Personnel Roles and Responsibilities

**Principal Investigator:** Signs the protocol agreement and the Form FDA 1572. The principal investigator is responsible for protocol adherence and execution, passage of the protocol through the Gorgas Institutional Bioethics Committee, overall data integrity, applying for and receiving approval for any modifications to the protocol or informed consent/assent form, training of sub-investigators, ensuring safety of the volunteers, briefing potential participants, obtaining proper informed consent/assent, determining study eligibility based on screening data and the exclusion criteria, reporting of any AEs or protocol deviations, and full accountability and proper storage of investigational products. The principal investigator assures overall coordination of the study and the filing of a final clinical study report with the sponsor.

**Sub-investigators:** As designated by the principal investigator, responsible for protocol adherence and execution, passage of protocol through local protocol committees, data integrity, applying for and receiving approval for any modifications to the protocol or consent/assent form, ensuring safety of the study subjects, managing and reporting of any AEs, briefing potential participants, obtaining proper informed consent/assent, determining study eligibility based on screening data and the exclusion criteria, and recording all observations and data in the individual subject records.

**Research Monitor:** Responsible for providing safety monitoring of research subjects for conditions that may arise during the conduct of the study. The research monitor is required to review all unanticipated problems involving risk to subjects or to others, SAEs, and all subjects deaths associated with the protocol and provide an unbiased written report of the event. At a minimum, the research monitor should comment on the outcomes of the event or problem and, in the case of a SAE or death, comment on the relationship to participation in the study. The research monitor should also indicate whether he/she concurs with the details of the report provided by the study investigator. Reports for events determined by either the investigator or research monitor to be possible or definitely related to participation and reports of events resulting in death should be promptly forwarded to the sponsor and the USAMRMC ORP HRPO. The research monitor will forward reports to the US Army Medical Research and Materiel Command, ATTN: MCMR-ZB-QH, 504 Scott Street, Fort Detrick, MD 21702-5012 USA and to usarmy.detrick.medcom-usammda.mbx.usamrmc-regulatory-affairs@mail.mil

**Clinical Trial Monitors:** Monitor the conduct of the study to determine if it is being executed according to the protocol and report their findings to the sponsor. The monitors will assure that submitted data are accurate and in agreement with source documentation; verify that investigational products are properly stored and accounted for, verify that subjects’ consent/assent for study participation has been properly obtained and documented, confirm that research subjects entered into the study meet inclusion and exclusion criteria, and assure that all essential documentation required by GCP guidelines are appropriately filed.

**Experts and Consultants:** Provide expert advice to the principal investigator, the sponsor, and the study team during the planning and conduct of the study on matters of the nature of the disease process, the treatment of the disease, and other matters to assist in the conduct of the study.

**Product Manager:** Responsible for the overall management of the product development effort.

**Clinical Data Management and Analysis:** Prepare and provide CRFs to clinical site; design, develop, and validate the clinical trial database; perform data entry into the clinical trial database; and perform database quality control and data analysis.

1. Body Chart of Lesion Sites


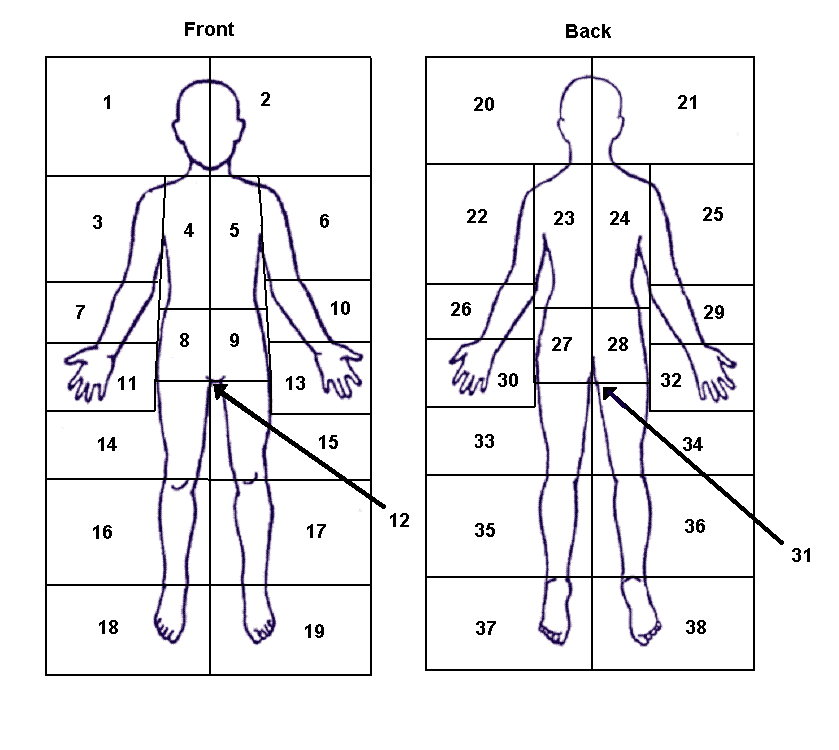

Supplement: S2 Appendix — (DOCX) [file pntd.0007253.s003.docx]
