# Supplementary material for: Topical paromomycin for New World cutaneous leishmaniasis
Source: PLoS Negl Trop Dis. 2019 May 2;13(5):e0007253. doi: 10.1371/journal.pntd.0007253 (PMC6497224; doi:10.1371/journal.pntd.0007253)
Supplement: S3 Appendix — (PDF) [file pntd.0007253.s004.pdf]

STATISTICAL ANALYSIS PLAN

for

PROTOCOL NO.: S-12-21

A Randomized, Double-blind, Pivotal Phase 3 Study of WR 279,396 (Paromomycin + Gentamicin Topical Cream) and Paromomycin Alone Topical Cream for the Treatment of Cutaneous Leishmaniasis in Panama

Protocol Version No.: 8.0   Version Date: 25 August 2014

Study Sponsor:

The Surgeon General, Department of the Army

Plan Authors:

Charles Scott, PhD
Senior Biostatistician
Janet H. Ransom, PhD
President
Fast-Track Drugs & Biologics, LLC
5 Paramus Ct.
North Potomac, Maryland 20878

Version Number 2.0

Date: 10Jul2015

STATISTICAL ANALYSIS PLAN
IND 50098
PROTOCOL NO.: S-12-21

A Randomized, Double-blind, Pivotal Phase 3 Study of WR 279,396 (Paromomycin + Gentamicin Topical Cream) and Paromomycin Alone Topical Cream for the Treatment of Cutaneous Leishmaniasis in Panama

Date: 10 July 2015

Prepared by: [signature] Date: 7/16/15
Charles Scott, PhD
Senior Biostatistician
Fast-Track Drugs & Biologics, LLC

Prepared by: Janet H. Ransom Date: 7/20/2015
Janet Ransom, PhD
President
Fast-Track Drugs & Biologics, LLC

Approved by: W F McCarthy Date: 7/16/15
William McCarthy, PhD
Chief, Biostatistics
USAMMDA, CSSD

Table of Contents

1. List of Abbreviations 4
2. Introduction 5
3. Protocol Summary 6
   - 3.1. Study Objectives 6
   - 3.2. Study Design 6
   - 3.3. Study Endpoints 7
4. Definition of Analysis Sets 8
5. Assessment and Justification of Study Endpoints 8
   - 5.1. Efficacy Endpoints 8
   - 5.2. Safety Endpoints 11
6. Hypotheses to be Tested 13
7. Sample Size Considerations 13
8. Data Quality Assurance 15
9. Procedure for Unblinding Data 17
10. Statistical Considerations 17
    - 10.1. General Considerations 17
    - 10.2. Participant Accountability and Protocol Deviations 17
    - 10.3. Demographics and Other Baseline Characteristics 18
    - 10.4. Study Drug Exposure 18
    - 10.5. Efficacy Analysis 18
      - 10.5.1. Primary Efficacy Analysis 19
      - 10.5.2. Secondary Efficacy Analysis 19
      - 10.5.3. Percentage of Subjects with All Lesions Cured 19
      - 10.5.4. Percentage of All Lesions Cured 19
      - 10.5.5. Lesion Area Measurements 19
      - 10.5.6. Ulcerated Lesion Cure Rates 20
      - 10.5.7. Time to Initial Clinical Cure 20
      - 10.5.8. Subgroup Analyses 20
      - 10.5.9 Additional Descriptive Analyses 20
        - 10.5.8.1. New Lesions Incidence 20
        - 10.5.8.2. Evidence of Infiltration of Lesions 21
    - 10.6. Safety Analysis 21
      - 10.6.1. Adverse Events and Serious Adverse Events 21
      - 10.6.2. Mucosal Examinations 21
      - 10.6.3. Clinical Laboratory Data 21
      - 10.6.4. Concomitant Medications 21
      - 10.6.5. Handling of Missing Data 21
11. Validation of Programming Code 22
12. Appendix A: Table Shells 23
13. Appendix B: Figure Shells 69
14. Appendix C: Listing Shells 74

1. List of Abbreviations

Abbreviation	*Definition*
AE	Adverse event
ALT	Alanine aminotransferase
AST	Aspartate aminotransferase
ATC	Anatomic, Therapeutic, and Chemical
CFB	Change from baseline
CI	Confidence interval
CL	Cutaneous leishmaniasis
CRF	Case report form
FDA	Food and Drug Administration
g	Grams
ID	Identification
I/E	Inclusion/Exclusion
L braziliensis	*Leishmania braziliensis*
L guyanensis	*Leishmania guyanensis*
L panamensis	*Leishmania panamensis*
MedDRA	Medical Dictionary for Regulatory Activities
max	Maximum
mg/dL	Milligrams per deciliter
min	Minimum
mITT	Modified intention-to-treat
mmHg	Millimeters of mercury
n	Number of observations
PCR	Polymerase chain reaction
QA	Quality Assurance
RFLP	Restriction fragment length polymorphism
SAE	Serious adverse event
SAP	Statistical Analysis Plan
SD	Standard Deviation
SOC	System, Organ, Class
SOP	Standard operating procedure
SPA	Special protocol assessment
U/L	Units per liter
USAMMDA	U.S. Army Medical Materiel Development Activity
USAMRMC	U.S. Army Medical Research and Material Command
WHO	World Health Organization

2. Introduction

This analysis plan describes the core set of table summaries, data listings, graphic representations, and pre-planned statistical analyses to address the study objectives for protocol number S-12-21. In addition, *post hoc* exploratory analyses may also be performed to aid in interpretation of the data. This plan will be subject to final review and approval by the Surgeon General, Department of the Army. Any prospective changes to the plan will be made, reviewed, and approved before the database is locked and unblinded for analysis. Changes to the planned conduct of the analysis will be documented and justified in the final clinical study report. Statistical analyses and report preparation will be performed by Fast-Track Drugs and Biologics, LLC (Fast-Track) statisticians.

3. Protocol Summary

3.1. Study Objectives

The primary objective of this double-blind study is to determine if WR 279,396 results in statistically superior ($p<0.05$) final clinical cure rates of an index lesion when compared with Paromomycin Alone for the treatment of New World cutaneous leishmaniasis (CL) in Panama.

Secondary objectives include evaluating other efficacy endpoints including: 1) percentage of subjects with all lesions cured; 2) percentage of all lesions cured; 3) lesion sizes over time; 4) cure rates over time, 5) median time to initial cure, and 6) relapse rates to determine if these endpoints are superior in the WR 279,396 group compared with the Paromomycin Alone group. An additional secondary objective is to determine the safety of both topical creams in this study population.

3.2. Study Design

This study is a pivotal Phase 3, randomized, double-blind, 3-site, two-group trial assessing the efficacy and safety of WR 279,396 Topical Cream and Paromomycin Alone Topical Cream in subjects with CL in Panama. The study schema is shown in Figure 1. Subjects will be recruited from three regions in Panama known to be endemic for *L panamensis* CL. Subjects will be screened over a period up to 28 days for eligibility including parasitology for confirmation of ulcerative CL and absence of early signs of mucosal disease that might be due to *Leishmania*. Other screening measures include vital signs, physical examination including the nasal and oral mucosa, medical history, and clinical chemistry tests. If eligible, subjects will be randomized using site as a stratification variable in a target ratio of 1:1 to receive either WR 279,396 (15% paromomycin + 0.5% gentamicin topical cream) (target n = 150) or Paromomycin Alone (15% paromomycin topical cream) (target n = 150) by topical application to CL lesions once daily for 20 days. Safety will be assessed by monitoring AEs, lesion site reactions, and blood creatinine, aspartate aminotransferase (AST), and alanine aminotransferase (ALT) levels.

After completing treatment, subjects will have an in-clinic follow-up on Study Days 35 ± 2 days, 49 ± 4 days, 63 ± 7 days, 100 ± 14 days, and 168 ± 14 days. Follow-up evaluations on 63 ± 7 days, 100 ± 14 days, and 168 ± 14 days include assessment of AEs, medication use, lesion measurements, physical examination of the oral and nasal mucosa for presence of mucosal leishmaniasis, and lesion photographs.

The primary efficacy analysis will be by evaluation of cure of an index lesion with secondary efficacy analyses including cure of all lesions in a subject and cure of all lesions per treatment group. Efficacy will be assessed by measuring the size of the index lesion ulcer, non-index lesions ulcers, and overall size of other non-ulcerated lesions at baseline (before the start of treatment), and on Study Days 20, 35 ± 2 days, 49 ± 4 days, 63 ± 7 days, 100 ± 14 days, and 168 ± 14 days. A notation will be made if clinical evidence of parasite persistence (infiltration) is observed at the Day 63 and beyond visits including significant erythema and induration when a lesion has otherwise completely ulcerated to document any subjects removed from the study early if the investigator judges them to be in need of rescue treatment.

Figure 1. Study Schema

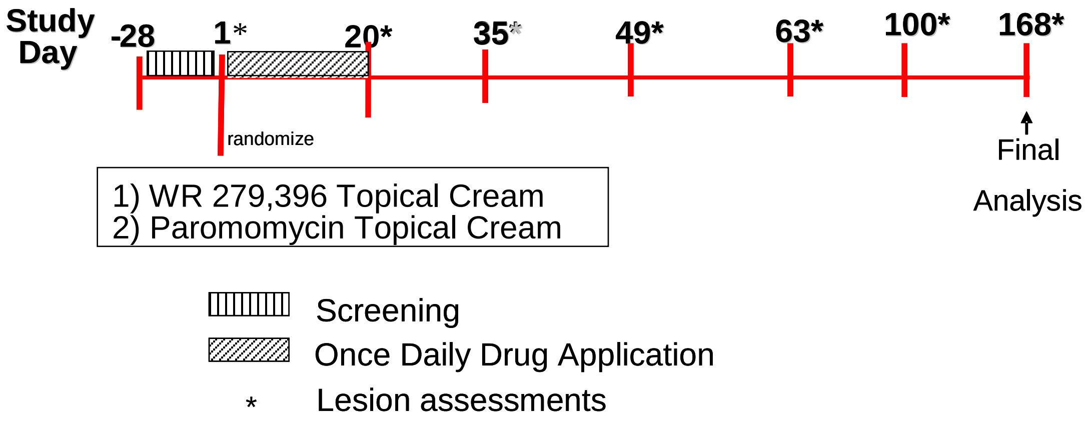

3.3. Study Endpoints

Efficacy Endpoints Include:

Primary Efficacy Endpoint

The primary efficacy endpoint is percentage of subjects with final clinical cure. **Final clinical cure** is defined as follows:

1. Subject has initial clinical cure (100% re-epithelialization of index lesion by nominal Day 63); OR, subject has initial clinical improvement (> 50% re-epithelialization of index lesion by nominal Day 63) followed by 100% re-epithelialization of the index lesion on or before nominal Day 100;

AND

2. Subject has no relapse of index lesion.

Relapse is defined as an index lesion meeting the criteria for initial clinical cure that had any new ulceration/nodule (> 0 x 0 mm measurement) by nominal day 168, or an index lesion meeting the criteria for initial clinical improvement that subsequently enlarged by nominal Day 168.

Failure is the opposite of cure: Subjects who drop out of the study early (ie, are not assessed at the final Day 168 visit) will also be considered clinical failures.

Secondary Efficacy Outcomes:

Secondary efficacy endpoints are as follows:

1) Percentage of subjects with all lesions cured, defined as:
   a. Final clinical cure as defined above (which is based solely on the index lesion); AND,
   b. Cure of all other lesions by nominal Day 100 (100% re-epithelialization of all ulcerated lesions and resolution of all other types of lesions)

2) Percentage of all lesions cured at Day 168
3) Area of ulceration of the index lesion at each measurement time point
4) Area of ulceration all treated lesions at each measurement time point
5) Ulcerated lesion cure rate at each measurement time point (complete cure is defined as 100% re-epithelialization of an ulcerated lesion)
6) Median time to initial clinical cure (100% re-epithelialization of the index lesion)

Safety Endpoints Include:

Safety endpoints are as follows:

1) AEs including application site reactions including elicited examination for pain, and clinician examination for erythema/redness, swelling/edema, and vesicles. Physical examination findings of evidence of mucosal leishmaniasis will be reported as an AE.
2) Blood creatinine, AST, and ALT

4. Definition of Analysis Sets

For Efficacy Outcome Measures:

Modified Intention-to-Treat (mITT) Analysis Set: The mITT analysis set includes all subjects who received any administration of an investigational product.

Evaluable Analysis Set: The evaluable analysis set will include all subjects who received daily administrations of investigational product for at least 18 of the total 20 days and had lesion measurements at Day 63 and 168.

For Safety Outcome Measures:

Safety Analysis Set: The safety analysis set includes all subjects who received any administration of investigational product. This analysis set is the same as the mITT analysis set.

5. Assessment and Justification of Study Endpoints

The schedule of Assessments for the study is shown in **Table 1**.

5.1. Efficacy Endpoints

CL Lesion Assessments. During screening, the number, anatomical location, general appearance (ulcerated, non-ulcerated), and evidence of a secondary infection of each CL lesion to be treated will be recorded. At the discretion of the investigator, appropriate ulcerative lesions will be scraped and aspirated and the sample will be smeared onto a slide for Giemsa staining. Additionally, a sample will be placed into culture medium for diagnostic confirmation of *Leishmania*. Those parasites from lesions for which a diagnostic specimen was collected will be identified by isoenzyme analysis and/or polymerase chain reaction with restriction fragment length polymorphism (PCR/RFLP), if possible. An index lesion will be identified.

Efficacy will be assessed by measuring the size of the index lesion ulcer, non-index lesions ulcers, and overall size of other non-ulcerated lesions at baseline (before the start of treatment), and on Study Days 20, 35, 49, 63, 100, and 168. The length and width of all ulcerated lesions will be measured in millimeters (mm). Non-ulcerative lesions will also be measured, and the length and diameter of these lesions will be reported. Non-ulcerated lesions have the appearance of a fried egg with a raised center that may be verrucous or plaques with a surrounding area of swelling or erythema. The raised center of these lesions will be the areas to be treated and measured. Evidence of infiltration (clinical evidence of parasite persistence) will be recorded at follow-up visits.

Re-epithelializations of ulcerated lesions are the generally accepted definition of clinical cure for CL. However, parasites may still be present in completely re-epithelialized skin and lesions may recur. Thus, the primary efficacy outcome measure includes evaluations of lesion sites for 168 days after the start of treatment to determine if a recurrence (relapse of disease) has occurred.

Parasitology of ulcerated lesions will be performed by lesion scraping and aspiration followed by culture and microscopic examination. Proof of infection due to *Leishmania* will be documented through either the demonstration of motile promastigotes in aspirate cultures or microscopic identification (ID) of leishmania amastigotes by DifQuik or Giemsa staining. A positive result from either method of at least one lesion that meets the criteria for use as an index lesion will be considered a confirmed diagnosis of CL during screening. Whenever possible, species identification will also be performed using isoenzyme analysis or PCR/RFLP methodologies. PCR/RFLP analysis will be performed by the Instituto Conmemorativo Gorgas de Estudios de la Salud. Isoenzyme analysis will be performed by Walter Reed Army Institute of Research, Silver Spring, Maryland. Isoenzyme analysis is considered the gold standard for species identification; however, not all specimens will result in active cultures that can be expanded to provide sufficient parasites for this assay. PCR, RFLP and other molecular techniques are in use in a few specialized, reference centers.

Table 1. Schedule of Procedures

	Screening	Treatment Period			Follow-up Period				
**Procedure**	**-28 to -1**	**Day -1/1**	**Day 2-19**	**Day 20**	**Day 35±2**	**Day 49±4**	**Day 63±7**	**Day 100±14**	**Day 168±14**
Informed consent/assent	X								
Demographics	X								
Medical history	X								
Leishmaniasis history	X								
Physical examination[a]	X						X	X	X
Parasitology[b]	X								
Vital signs[c]	X								
Lesion measurements	X	X		X	X	X	X	X	X
Clinical chemistry[d]	X			X					
Pregnancy test	X								
Eligibility checklist		X							
Randomization[e]		X							
Study drug application		X	daily	X					
Photograph of lesions		X		X	X	X	X	X	X
Adverse events		X	daily	X	X	X	X	X	X
Prior and concomitant medications	X	X	once per week	X	X	X	X	X	X

[a] Physical examination includes a general physical exam and at baseline and follow-up an intensive evaluation of the mucosa.
[b] Parasitology includes culture of aspirated lesions for promastigotes, microscopic identification of Leishmania amastigotes on Giemsa stain, and PCR/RFLP.
[c] Vital signs include oral temperature, and sitting blood pressure and heart rate.
[d] Clinical chemistry measurements include creatinine, AST, and ALT during screening and Day 20.
[e] Day 1.

5.2. Safety Endpoints

Adverse Events. AEs will be assessed by asking subjects general questions about their well being at each study visit, by assessing lesions during treatment, by evaluating clinical chemistry measurements at the end of treatment, and by physical examination of the mucosa and palate for evidence of mucosal leishmaniasis.

Severity: All AEs (except application site reactions and pain in the lesions described below) will be graded according to the following definitions. Assignment of grade based on the intensity of symptoms and the degree of limitation of usual daily activities will be done according to severity using the following criteria:

Grade 1: Mild symptoms invoking a minimum degree of discomfort that are easily tolerated.

Grade 2: Moderate symptoms that result in a reduction in normal daily activity, but is not totally incapacitating. This may or may not require medical intervention.

Grade 3: Severe symptoms that may be totally incapacitating or result in marked reduction in normal daily activity. Medical intervention is usually required.

Grade 4: Potentially life-threatening event that requires emergency intervention or hospitalization.

Application site reactions including erythema/redness, swelling/edema, and vesicles will be scored according the following criteria. If there is a change in the application site compared to baseline (ie, an increase in grade), then this will be recorded as an AE. Each application site will be assessed for the above reactions and the highest severity grade of any application site will be recorded on the AE CRF.

Grade 0: No evidence of erythema/redness, swelling/edema, or vesicles.

Grade 1: Visibly present but not associated with any other symptoms.

Grade 2: Visibly present, large area around lesion site, and associated with other symptoms such as itching or pain. Medical intervention may be required.

Grade 3: Severe symptoms that require medical intervention.

Pain in the lesions prior to study drug administration (assessed before the daily lesion cleaning) will be graded according to the following criteria:

Grade 0: None (feels no pain)

Grade 1: Mild pain (does not interfere with daily activity)

Grade 2: Moderate pain (interferes with daily activity)

Grade 3: Severe pain (daily activities are interrupted)

Relationship: The degree of certainty for which the AE/SAE is attributed to the investigational product or alternative causes will be determined by the investigator's use of clinical judgment in conjunction with the assessment of a plausible biologic mechanism, a temporal relationship between the onset of the event in relation to receipt of the investigational product, and identification of possible alternate etiologies including underlying disease, concurrent illness or concomitant medications.

The following drug relationships will be used for this clinical study:

Not related: There is no relationship to investigational product. Applies to those events for which evidence exists that there is an alternate etiology.

Unlikely: Likely unrelated to the investigational product. Likely to be related to factors other than investigational product, but cannot be ruled out with certainty.

Possible: An association between the event and the administration of investigational product cannot be ruled out. There is a reasonable temporal association, but there may also be an alternative etiology such as the subject's clinical status or underlying factors including other therapy.

Probable: There is a high degree of certainty that a relationship to the investigational product exists. There is a reasonable temporal association, and the event cannot be explained by known characteristics of the subject's clinical state or factors including other therapy.

Definite: An association exists between the receipt of investigational product and the event. An association to other factors has been ruled out.

The categories of definite, probable, and possible will be considered investigational product related with regards to summary statistics.

AE Actions and Outcomes: For each AE that is reported the actions taken with respect to study drug including: 1) none, 2) temporarily discontinued, or 3) permanently discontinued will be recorded on the AE CRF. Also, outcomes will also be recorded including: 1) resolved; 2) resolved with sequelae; 3) ongoing, and 4) unknown. If the AE required any treatment, this will also be recorded.

Serious Adverse Events (SAE). Each AE or reaction will be classified by the study investigator as serious or non-serious and the reason why it qualifies as an SAE in accordance with the following:

- results in death;
- is life-threatening; (NOTE: The term "life-threatening" in the definition of "serious" refers to an event in which the subject was at risk of death at the time of the event; it does not refer to an event which hypothetically might have caused death if it were more severe.)
- requires inpatient hospitalization or prolongation of existing hospitalization;
- results in persistent or significant disability/incapacity;
- is a congenital anomaly/birth defect; or
- important medical events that may not result in death, be life-threatening, or require hospitalization may be considered a serious adverse drug reaction, when based on appropriate medical judgment that may jeopardize the subject and may require medical or surgical intervention to prevent one of the outcomes listed above.

Physical Examination. A physical exam of the oral cavity, head, eyes, ears nose and throat, cardiovascular system, lungs, abdomen (liver/spleen), extremities, skin, lymphatic system, neuropsychiatric mental status and sensory/motor status, musculoskeletal system and general

appearance will be performed during screening. The Principal Investigator or designee will examine the nasal and oral mucosa of each subject. Each of 5 possible sites (nasal skin, nasal mucosa, palate, pharynx, and larynx) will be evaluated for 4 possible signs of disease (erythema, edema, infiltration, and erosion). On Days 63, 100, and 168 the nasal and oral mucosal examination will be repeated to determine if there is evidence of the *Leishmania* mucosal involvement.

Blood Chemistry. Creatinine, AST, and ALT will be measured during screening and on Day 20. Serum samples will be sent to the local hospital laboratory for analysis using standard clinical chemistry methods. The National Clinical Reference Laboratory (Laboratorio Central de Referencia en Salud Publica) will inspect and certify the local hospital laboratories at the subsites at Penonome and Bocas del Toro.

Vital signs to be measured during screening include oral temperature, sitting blood pressure, and heart rate.

6. Hypotheses to be Tested

The primary efficacy objective of this randomized, double-blind study is to demonstrate that in this population of persons with CL, WR 279,396 will achieve a higher index lesion cure rate compared to Paromomycin Alone. The null hypothesis for this superiority trial is that there is no difference in final index lesion cure rate between the two treatments. Although the primary interest in the superiority of WR 279,396, the study null hypothesis will be tested using a two-sided ($\alpha = 0.05$) uncorrected chi-square test for two independent proportions (final index cure rates).

7. Sample Size Considerations

In the previous Phase 2 study in Panama of similar design and entry criteria, 13 of 15 (86.7%) of the subjects treated with WR 279,396 had final clinical cure of the index lesion and 9 of 15 (60.0%) of subjects treated with Paromomycin Alone had final clinical cure of the index lesion. Final cure rates for subjects based on all lesions cured were 13 of 15 (86.7%) for WR 279,396 and 8 of 15 (53.3%) for Paromomycin Alone. Based on the results in this study, it is assumed that WR 279,396 would have a clinical cure rate of the index lesion of 80% (minimum). Sample sizes were estimated for effect sizes of 5%, 10%, 15%, and 20% differences in the final cure rates in the two groups as shown in Table 2 [based on uncorrected chi-square test, 80% power, 5% 2-sided type-one error rate (alpha)]. As the difference in cure rates between the two groups in the Phase 2 study was 26.7%, and recognizing the small size of this study and the inherent variability in the estimated effect size based on the small sample size, a total of 150 subjects per treatment group -- representing a difference between groups of 15% -- were initially selected for this Phase 3 study. Assuming a clinical cure rate of 80% for WR 279,396 and 65% for Paramomoycin Alone, a sample size of 150 per group would have a power of 83% power to detect the difference of 15 percentage points.

Table 2: Sample Size Estimates

True Final Clinical Cure Rate (%) for the WR 279,396 Group	True Final Clinical Cure Rate (%) for the Paromomycin Group	Difference (%) between groups	n[a]
80	75	5	1094
80	70	10	294
80	65	15	138
80	60	20	82

[a] Sample size per group based on uncorrected chi-square test with 80% power and two-sided alpha = 0.05. Calculations were performed with nQuery version 6.02.

During the conduct of the study, new *Leishmania* species were identified in Panama that were not previously observed during the conduct of the Phase 2 study. Other species included *Leishmania guyanensis* (*L guyanensis*) and *Leishmania braziliensis* (*L braziliensis*) in addition to *L panamensis*. Of 149 subjects randomized to the study for which speciation data were available (treatment groups blinded and combined) in August 2014, 74% of subjects had *L panamensis* and the others were split between *L guyanensis* (19%) and *L braziliensis* (6%). Since only *L panamensis* was identified in the Phase 2 study, and responses to treatments for Leishmaniasis may vary by species, then the effect of the species on the expected cure rates is unknown. Conservatively assuming the cure rates in the other species is 60% for WR 279,396 and is 50% in the Paromomycin Alone group, then the expected cure rate becomes 75% in WR 279,396 [(0.75 x 80%) + (0.25 x 60%)] and 61.25% in Paromomycin Alone [(0.75 x 65%) + (0.25 x 50%)]. Under these asumptions, the expected difference in cure rates is reduced from 15% (80% - 65%) to 13.75% (75% - 61.25%). A sample size of 200 subjects per arm will provide at least 84% statistical power to detect a difference of 13.75% in cure rates between WR 279,396 and Paromomycin Alone. Therefore, the total sample size was adjusted to 400 subjects (200 per group). This change was instituted in version 8 of the protocol (22Aug2014). An amendment to the special protocol assessment (SPA) was sent and approved by the FDA for this change.

8. Data Quality Assurance

Data management will be performed according to the data management plan prepared for this study and approved by the study sponsor. The clinical data management and analysis team from Fast-Track Drugs & Biologics, LLC is responsible for preparing and providing case report forms (CRFs) to the clinical site. They will design, develop, and validate the clinical trial database, perform data entry into the clinical trial database, perform database quality control and data analysis. All study data will be collected on CRFs.

Data quality assurance will start with training of clinical investigative staff on data collection and assessment procedures including a Study Procedures Manual that described what data to collect, how to assess and report AEs, and procedures for completion of CRFs. All data will be subjected to 100% audit against source documents by USAMMDA or Fast-Track clinical monitors.

CRFs were created using an established data dictionary conforming to CDISC Study Data Tabulation Model naming conventions for each variable including the field name, field type, field attributes, and coding for variables. Range checks, alpha-numeric requirements, and null/not null parameters were programmed as applicable. Subject identification (ID) numbers will be subjected to database lookup constraints. The back end database application will be Microsoft Access. The data management application was validated according to Fast-Track's standard operating procedures. All CRF data will undergo a double-data entry procedure prior to being exported to the clinical database.

After data entry, programmed edits checks of database will be performed using MS Access Query and SAS. A study statistician working together with Fast-Track data management will create edit check specifications. Edit checks will be written to detect anomalies in the database. These checks will address inconsistencies (within visits, across visits), invalid/unusual values, missing values, and protocol violations. Edit checking will be validated on test data. In addition to programmed edit checks, quality control examination of data will also be performed by reviews of data listings. The edit check plan is included in the data management plan for the study.

Some of the data included in the database will be coded by a qualified coder including AEs using the MedDRA dictionary (current version) using a system organ class (SOC) for AEs and medical history findings and preferred term for both. Medications will also be coded using World Health Organization (WHO) drug codes including medication preferred term, medication class, and medication Anatomic, Therapeutic, and Chemical (ATC) code.

Upon completion of data entry, review, and edit checking of the database, an independent Fast-Track quality assurance (QA) auditor will conduct a database audit prior to a database lock. The auditor will compare printed copies of the database listings to the data on the CRFs. QA will audit 100% of all data defined in the data management plan for all participants. The auditor will note any discrepancies and report them to data management. All discrepancies will be resolved by data queries or database error resolution issued by data management. The database will continue to be audited and corrected until it passes with a 0% error rate for critical fields and <0.5% error rate for non-critical data fields. Upon passing the quality assurance audit of the database, approval of the sponsor to lock the database, and issuance of the audit certificate, the database will be locked for clinical analysis.

9. Procedure for Unblinding Data

This study is a double-blind study. If the treatment assignment for an individual subject needs to be unblinded during the course of the trial, the Principal Investigator will contact the unblinded sponsor designee or the data management center biostatistician for the treatment assignment. A note to the file will be prepared by the person who performed the unblinding to indicate when the subject was unblinded, why the subject was unblinded, and to whom the unblinding information was given. Unblinding of a single subject should only be done in the event that the identity of the Topical Cream is needed to make decisions on what types of treatment to provide a subject in need of medical attention when this information will influence this decision. It is not anticipated that unblinding for a single subject will be needed during the course of the trial.

Blinding and unblinding procedures will be performed in accordance with Fast-Track's standard operating procedures (SOPs) CT017 Procedures for Unblinding Investigational Agent Identity for a Study or a Single Subject, CT024 Preparation of Randomization Codes and Maintenance of Treatment Assignment Schedules, CT033 Security and Access to Clinical Trial Databases, and CT045 Development of Clinical Data Management Plans.

A representative of the sponsor and a single biostatistician from Fast-Track as designated in the Data Management Plan will be unblinded during the study. The treatment assignments will not be added to the main study database until locking approval has been received prior to data analysis.

10. Statistical Considerations

10.1. General Considerations

Summary tables (descriptive statistics and/or frequency tables) and/or data listings will be provided for all baseline variables, efficacy variables, and safety variables as appropriate. Continuous variables will be summarized with the number of observations (n), mean, standard deviation (SD), median, minimum, and maximum values. Categorical variables will be presented as counts and percentages. Ninety five (95) percent confidence intervals (CI) will be presented as appropriate. All data will be presented separately by treatment group. Both the primary and secondary efficacy endpoints will be presented for the mITT and evaluable analysis sets. All statistical tests will be two-sided. P-values < 0.05 will be considered statistically significant. In addition, to pre-specified efficacy analyses, *post hoc* analyses may also be performed to further explore the data to aid in interpretation.

Analyses will be done using SAS v9.4.

10.2. Participant Accountability and Protocol Deviations

The disposition of all study subjects will be summarized including the total numbers screened, eligible, randomized, evaluable, withdrawn, and completed. Accountability data including study termination and reason will be provided in a summary table and listing for all subjects who were randomized. A summary of all significant protocol deviations and a listing of all protocol deviations will be provided.

10.3. Demographics and Other Baseline Characteristics

Summary tables of subject demographics including age, age groups, gender, ethnicity and race by group and in total will be presented. Summary tables of the number of baseline lesions, lesion areas, evidence of secondary infection, lesion characteristic (ulcerated versus non-ulcerated) and length of time between initial presence of current lesions, parasitology findings, and prior treatment for CL will also be presented by group and in total. Parasite species will be summarized for the index lesion by method (isoenzyme electrophoresis and PCR/RFLP). If the parasite species for the index lesion was not identified but another concurrent lesion in the subject was identified, then the primary infecting species will be that for any lesion for which a species could be determined. For this analysis, if the species was not identified by isoenzymes or PCR/RFLP, it will be assumed to be due to *L panamensis* because this species is most prevalent by epidemiology in this region. These data will be summarized for both the mITT and evaluable populations. Demographics for the subset of subjects who were categorized as having an *L panamensis* infection will also be presented. Screening medical history findings and vital signs will be presented in summary tables. By-subject listings will be provided for all of the above data.

The comparability among the treatment groups with respect to the demographic and baseline variables will be evaluated by appropriate statistical methods. These will include t-tests for continuous variables, chi-square tests for categorical, and Fisher's exact test for binary variables. If the continuous data is skewed then the Wilcoxon rank sum test will be used.

10.4. Study Drug Exposure

Summary statistics for the days of study drug application and total amount of drug applied (g) will be presented for the safety analysis set by treatment group during the 20-day treatment period. Daily exposure will be estimated by dividing the total exposure in grams by the number of days the subject was treated. Daily exposure by area of the ulcerated skin exposed will be estimated by dividing the total exposure in grams by the number of days the subject was treated by the average of the ulcerated lesion area of all lesions at baseline and end of treatment. As the ulcer area typically increases over time during treatment, the average area is at best an approximation of ulcer area during treatment. By subject listings of drug exposure will be provided.

The protocol allowed for the possibility of new lesions. These could occur during the initial treatment period or after the initial 20-day treatment period. In order to estimate the total drug exposure throughout the study to a single subject, the amount of study drug applied after the initial 20-day treatment period will also be summarized. This will be determined by subtracting the weight of all tubes assigned to the subject at the end of the study from the weight of these tubes at the end of the 20-day treatment period.

10.5. Efficacy Analysis

All efficacy analyses will be performed in both the mITT and evaluable sets. Analyses of endpoints for cure will consider subjects with missing data as failures. The area of ulceration of lesions will be calculated using the area calculation for an ellipse as follows:

Area = (A/2)*(B/2)*Pi mm^2

Where A = longest diameter of ulceration in mm

B = perpendicular to “A” diameter of ulceration in mm

Pi = 3.1416

The same area calculation applies to non-ulcerated lesions.

10.5.1. Primary Efficacy Analysis

Final clinical cure rates of the index lesion (proportions) will be compared between the two treatment groups by uncorrected chi-square test using the mITT group. The difference in cure rates and 95% confidence intervals (CI) for the difference will also be presented. A stepwise logistic regression analysis with the dependent variable of final cure and independent variables of treatment group, and possibly age of lesion, size of index lesion, species, and subgroups (Section 10.6.2) with significant interaction effects will be performed. Coefficients for the independent variables will be tested using the Wald statistic. Factors that do not meet the inclusion criteria of 0.20 will not be included in the final logistic regression model.

10.5.2. Secondary Efficacy Analysis

No adjustments will be made to correct for multiplicity of comparisons of secondary efficacy endpoints. By-subject listings will be provided for all the secondary efficacy endpoints.

10.5.3. Percentage of Subjects with All Lesions Cured

Final clinical cure rates based on the proportions of subjects with all lesions cured will be compared between the two treatment groups using uncorrected chi-square test. The difference in cure rates and 95% CI for the difference will also be presented.

10.5.4. Percentage of All Lesions Cured

Final clinical cure rates based on the proportion of all lesions treated will be compared between the two treatment groups by uncorrected chi-square test. The difference in cure rates and 95% CI for the difference will also be presented.

10.5.5. Lesion Area Measurements

Summary descriptive statistics for area measurements including total area of ulceration of the index lesion and all ulcerated lesions by subject, percentage change from baseline at each measurement time point for each index lesion and by all lesions will be presented both in tabular form and graphically. Areas over time will be compared between the two treatment groups using mixed effects models with treatment and time in the model as fixed effects and subjects as random effects. Data will be transformed to better satisfy the model assumptions. It has been observed that lesions that are close to one another prior to the start of treatment may have overlapping areas of ulceration by the end of treatment making it impossible to distinguish from one another. In these cases, the combined lesion is measured and recorded as the area of the largest of the lesions at baseline and the area for the smaller of the lesions is left blank as no separate measurement can be correctly made. After the combined lesions have completely re-epithelialized, then the lesion measurement for both lesions is recorded separately as 0 x 0.

Summary statistics for lesion areas will therefore have missing data for which no adjustments will be made.

10.5.6. Ulcerated Lesion Cure Rates

The percentage of subjects at each measurement time point with complete cure of the index lesion and all ulcerated lesions will be presented. A complete cure of an ulcerated lesion is defined as 100% re-epithelialization of the lesion (a measurement of ulceration of 0 x 0 mm). The percentages of subjects with 100% re-epithelialization of the index lesion and all ulcerated lesions over time will be compared between treatment groups using generalized estimating equations model with treatment and time in the model.

10.5.7. Time to Initial Clinical Cure

Time to initial clinical cure will be determined using product-limit method and will be presented graphically (Kaplan-Meier plots). Time to initial clinical cure will be compared between the two groups using log-rank test. Missing data will be considered censored at the time of the last assessment for this analysis. It should be noted that with the limited number of time points for cure to be assessed, that this analysis may not be very sensitive to differences.

10.5.8. Subgroup Analyses

The primary efficacy endpoint (proportion of index lesions that were cured) and the secondary efficacy endpoint of proportion of patients with all lesions cured will be analyzed for the following subgroups. The difference in cure rates between treatment groups within levels of the factor and 95% CI for the difference will also be presented. Within subgroup level two treatment groups will be compared by uncorrected chi-square test. A Cochran-Mantel-Haenszel will test if there are treatment differences across the subgroup levels. These analyses will be done for the mITT and evaluable sets of subjects.

- Gender (male and female)
- Age (<12 years, 12 to 17 years, and ≥ 18)
- Drug exposure: total grams (g) of cream applied (low: < median g of cream applied; high: ≥ median g of cream applied)
- Clinical signs of secondary infection at baseline
- Infecting species

10.5.9 Additional Descriptive Analyses

10.5.8.1. New Lesions Incidence

The number of subjects with new lesions and the number of new lesions (if any) that develop during the study will be summarized. There is a provision in the protocol that new lesions can be treated with the topical cream to which the subject was randomized; therefore, the cure rates (100% re-epithelialization of ulcerated lesions or resolution of non-ulcerated lesions) for those new lesions that were treated will also be presented in a summary table. There will be different follow-up periods for the new lesions that were treated, therefore, the table will show the cure rates, relative to the end of the course of treatment. As new lesions may develop at any time, the

time course of the development of new lesions will also be presented as a figure for both the number of patients and the number of lesions.

10.5.8.2. Evidence of Infiltration of Lesions

The number and percentage of lesions with clinical signs of infiltration will be presented for each follow-up time point by group. Infiltration was assessed at all time points regardless if there was still a lesion ulcer present.

10.6. Safety Analysis

10.6.1. Adverse Events and Serious Adverse Events

AEs will be coded using the most recent version of the Medical Dictionary of Regulatory

Activities (MedDRA) preferred terms and will be grouped by system, organ, and class (SOC) designation. The severity, frequency, and relationship of AEs to study drug will be presented by preferred term by SOC grouping by treatment group. A summary of AEs occurring in more than 5% of subjects in one group will also be provided. A summary of the start day and duration of each AE will also be presented. Listings of each individual AE including start date, stop date, start day, severity, relationship, outcome, and duration will be provided.

The duration of the event will be calculated by subtracting the start date of the event from the stop date of the event and adding the integer one. Thus, if an AE started and stopped on the same day, then the duration is 1 day. Or if the AE started one day and stopped the next day, the duration is 2 days.

A listing of all participants experiencing SAEs will be provided.

10.6.2. Mucosal Examinations

A table summarizing the number and percentage of subjects with any signs of nasal irritation at each follow-up time point will be presented by group. The numbers of subjects with nasal irritation that was confirmed to be due to *Leishmania* will be presented in the summaries of AEs.

10.6.3. Clinical Laboratory Data

Blood creatinine, AST and ALT levels will be presented as summary statistics of serum concentrations at baseline and Day 20 and change from baseline. A summary and listing of analyte concentrations for subjects with levels above normal laboratory limits will also be presented. Pregnancy test results will be summarized for screening and Day 35 tests.

10.6.4. Concomitant Medications

Medications will be coded using WHO drug codes including medication preferred term, medication class, and medication ATC code. A summary by preferred term and medication class will be provided separately for prior medications and concomitant medications. A listing all medications will be provided for the safety analysis set.

10.6.5. Handling of Missing Data

Subjects who do not provide lesion response measurements at nominal Days 63 and 168 will be

considered clinical failures in the mITT analysis of the primary efficacy endpoint and the analysis of the secondary efficacy endpoint of final clinical cure of all lesions, unless the subject has documented clinical cure of the index lesion or all lesions before Day 63 and does not relapse by Day 168 (ie, there must be an assessment at nominal Day 168 but nominal Day 63 can be missing). If a subject misses the nominal Day 63 lesion assessment but had 100% re-epithelialization of the index lesion/all lesions before this visit and continues to have 100% re-epithelialization of all lesions by nominal Day 168, s/he will be considered a final clinical cure without relapse. Missing lesion measurements will be ignored when presented as summary statistics of lesion areas. Thus, the analysis of the primary outcome measure assumes a worst case-scenerio. The reasons for subject drop-outs will be presented. Missing data in the time to clinical response assessments will be censored at the last available data point. Missing data will be represented by numbers of data points used in the analysis as shown in the data tables.

11. Validation of Programming Code

All SAS code used to generate tables, listings, and figures will be validated and reviewed before being finalized. The validation process will be used to determine that the numbers are produced by a statistically valid method and that the execution of the computations is correct. Qualified personnel who have not previously been involved in the production of the original programming code will perform the validation procedures. Methods of validation include independent programming and comparison to data listings. Tables will be reviewed for accuracy, consistency with this plan, consistency within tables, and consistency with corresponding output. Once validation is complete, a quality control reviewer will perform a final review of the documents for accuracy and consistency. Upon completion of validation and quality review procedures, all documentation will be collected and filed in the study documentation files at Fast-Track.

12. Appendix A: Table Shells

List of Tables

Table 1: Summary of Subject Disposition – All Consented Subjects 26
Table 2: Reasons for Not Meeting Eligibility Criterion - Non-Randomized Subjects 27
Table 3: Demographics – mITT Subjects 28
Table 4: Demographics – Evaluable Subjects 29
Table 5: Demographics – *L panamensis* mITT Subjects 30
Table 6: Baseline Lesions Characteristics – mITT Subjects 31
Table 7: Baseline Lesions Characteristics – Evaluable Subjects 32
Table 8: Baseline Leishmaniasis History – mITT Subjects 33
Table 9: Baseline Leishmaniasis History – Evaluable Subjects 33
Table 10: Baseline Leishmaniasis History – *L panamensis* mITT Subjects 33
Table 11: Summary of Medical History 34
Table 12: Baseline Vital Signs – mITT Subjects 34
Table 13: Baseline Vital Signs – Evaluable Subjects 35
Table 14: Protocol Compliance – mITT Subjects 36
Table 15: Baseline Parasitology – mITT Subjects 36
Table 16: Baseline Parasitology – Evaluable Subjects 37
Table 17: Drug Exposure During Days 1 to 20 – mITT Subjects 38
Table 18: Drug Exposure During Days 1 to 20 – Evaluable Subjects 39
Table 19: Drug Exposure During Treatment of New Lesions (Post Day 20) – mITT Subjects 40
Table 20: Drug Exposure During Treatment of New Lesions (Post Day 20) – Evaluable Subjects 40
Table 21: Numbers and Proportion of Subjects Meeting Criteria for Final Clinical Cure of Index Lesions – mITT Subjects 41
Table 22: Final Clinical Cure of Index Lesion Logistic Regression – mITT Subjects 41
Table 23: Numbers and Proportion of Subjects Meeting for Final Clinical Cure of Index Lesions – Evaluable Subjects 41
Table 24: Final Clinical Cure of Index Lesion Logistic Regression – Evaluable Subjects 41
Table 25: Numbers and Proportion of Subjects Meeting Criteria for Final Clinical Cure of All Lesions – mITT Subjects 42
Table 26: Numbers and Proportion of Subjects Meeting Criteria for Final Clinical Cure of All Lesions – Evaluable Subjects 42
Table 27: Area of Ulceration and Change from Baseline of the Index Lesion – mITT Subjects 43

Table 28: Area of Ulceration and Change from Baseline of the Index Lesion – Evaluable Subjects . 44

Table 29: Area of All Lesions and Change from Baseline – mITT Subjects 45

Table 30: Area of All Lesions and Change from Baseline – Evaluable Subjects................................ 46

Table 31: Numbers and Proportion of Index Lesions Meeting Criteria for Clinical Cure During the Study – mITT Subjects 47

Table 32: Numbers and Proportion of Index Lesions Meeting Criteria for Clinical Cure Throughout the Study – Evaluable Subjects 47

Table 33: Generalized Estimating Equations of Index Lesions Meeting Criteria for Clinical Cure Across Time – mITT Subjects... 48

Table 34: Generalized Estimating Equations of Index Lesions Meeting Criteria for Clinical Cure Across Time – mITT Subjects... 48

Table 35: Numbers and Proportion of All Lesions Meeting Criteria for Clinical Cure During the Study – mITT Subjects 49

Table 36: Numbers and Proportion of All Lesions Meeting Criteria for Clinical Cure During the Study – Evaluable Subjects 49

Table 37: Generalized Estimating Equations of All Lesions Meeting Criteria for Clinical Cure Across Time – mITT Subjects.. 50

Table 38: Generalized Estimating Equations of All Lesions Meeting Criteria for Clinical Cure Across Time – mITT Subjects.. 50

Table 39: Median Time to Initial Clinical Cure of Index Lesions – mITT Subjects......................... 50

Table 40: Median Time to Initial Clinical Cure of Index Lesions – Evaluable Subjects 50

Table 41: Numbers and Proportion of Subjects with Clinical Signs of Infection of the Index Lesion – mITT Subjects 51

Table 42: Numbers and Proportion of Subjects with Clinical Signs of Infection of the Index Lesion – Evaluable Subjects... 51

Table 43: Numbers and Proportion of Subjects with Clinical Signs of Infection of All Lesions – mITT Subjects 52

Table 44: Numbers and Proportion of Subjects with Clinical Signs of Infection of All Lesions – Evaluable Subjects... 52

Table 45: Numbers and Proportion of Subjects Meeting Criteria for Final Clinical Cure of Index Lesions: Gender by Treatment Group – mITT Subjects 53

Table 46: Numbers and Proportion of Subjects Meeting Criteria for Final Clinical Cure of Index Lesions: Gender by Treatment Group – Evaluable Subjects.. 53

Table 47: Numbers and Proportion of Subjects Meeting Criteria for Final Clinical Cure of Index Lesions: Age Group by Treatment Group – mITT Subjects 54

Table 48: Numbers and Proportion of Subjects Meeting Criteria for Final Clinical Cure of Index Lesions: Age Group by Treatment Group – Evaluable Subjects....................................... 54

Table 49: Numbers and Proportion of Subjects Meeting Criteria for Final Clinical Cure of Index Lesions: Clinical Signs of Secondary Infection at Baseline by Treatment Group – mITT Subjects 55

Table 50: Numbers and Proportion of Subjects Meeting Criteria for Final Clinical Cure of Index Lesions: Clinical Signs of Secondary Infection at Baseline by Treatment Group – Evaluable Subjects........ 55

Table 51: Numbers and Proportion of Subjects Meeting Criteria for Final Clinical Cure of Index Lesions by Infecting Species Between Groups – mITT Subjects........ 56

Table 52: Numbers and Proportion of Subjects Meeting Criteria for Final Clinical Cure of Index Lesions by Infecting Species Between Groups – Evaluable Subjects 56

Table 53: Numbers and Proportion of Index Lesions with Signs of Lesion Infiltration During the Study – mITT Subjects 57

Table 54: Numbers and Proportion of Index Lesions with Signs of Lesion Infiltration During the Study – Evaluable Subjects 57

Table 55: Cumulative Numbers of Subjects with New Lesions and Cumulative Number of New Lesions – mITT Subjects 58

Table 56: Cumulative Numbers of Subjects with New Lesions and Cumulative Number of New Lesions – Evaluable Subjects 58

Table 57: Cure Rates of New Lesions that Received Treatment – mITT Subjects 59

Table 58: Cure Rates of New Lesions that Received Treatment – Evaluable Subjects........ 59

Table 59: Overall Number of Adverse Events – Safety Subjects 60

Table 60: Summary of Adverse Events in WR 279,396 Group – Safety Subjects........ 61

Table 61: Summary of Adverse Events in Paromomycin Group – Safety Subjects........ 62

Table 62: Summary of Adverse Events (Number and Percentage) Occurring in at Least 5% of Subjects in Any Group – Safety Subjects........ 63

Table 63: Subjects with Evidence of Mucosal Erythema, Edema, Infiltration, or Erosion at Follow-up Visits During the Study 63

Table 64: Adverse Events Time to Onset – Safety Subjects 64

Table 65: Adverse Events Duration – Safety Subjects 64

Table 66: Clinical Chemistry: Day 1 and 20 – Safety Subjects........ 65

Table 67: Number (%) of Subjects with Laboratory Values above the Upper Limit of Normal – Safety Subjects 66

Table 68: Pregnancy Test Results........ 67

Table 69: Summary of Concomitant Medication Use (Number and Percentage of Subjects) – Safety Subjects 68

Table 1: Summary of Subject Disposition – All Consented Subjects

Study Drug	WR 279,396	Paromomycin
Total Consented	xx	xx
Randomized n (%)	xx (x.x)	xx (x.x)
Data below is n (%) of randomized		
Safety/mITT analysis set	xx (x.x)	xx (x.x)
Evaluable analysis set	xx (x.x)	xx (x.x)
Participants completing study	xx (x.x)	xx (x.x)
Participants terminating early[a]		
Lost to follow-up	xx (x.x)	xx (x.x)
Withdrawal of consent by subject	xx (x.x)	xx (x.x)
Withdrawal of patient by investigator	xx (x.x)	xx (x.x)
Clinical evidence of continued presence	xx (x.x)	xx (x.x)
of disease	xx (x.x)	xx (x.x)
Worsening of disease	xx (x.x)	xx (x.x)
Recurrence of disease	xx (x.x)	xx (x.x)
Adverse event	xx (x.x)	xx (x.x)
Death	xx (x.x)	xx (x.x)
Other	xx (x.x)	xx (x.x)
Drug suspended	xx (x.x)	xx (x.x)
Adverse events	xx (x.x)	xx (x.x)
Allergic reaction	xx (x.x)	xx (x.x)
Pregnancy test positive	xx (x.x)	xx (x.x)
Other medical condition	xx (x.x)	xx (x.x)
Not eligible	xx (x.x)	xx (x.x)
Withdrew consent	xx (x.x)	xx (x.x)
Other	xx (x.x)	xx (x.x)

[a] Subjects may have been terminated for more than one reason, thus numbers will not add up to the total numbers of subjects.

<Program Name: Time and Date>

Table 2: Reasons for Not Meeting Eligibility Criterion - Non-Randomized Subjects

**Reason**	**n= xx**
Subject withdrew consent	n (%)
Negative parasitology	n (%)
Pregnant	n (%)
Breastfeeding	n (%)
> 10 lesions	n (%)
Significant organ abnormality	n (%)
Hypersensitivity to aminoglycosides	n (%)
Evidence of mucosal disease	n (%)
Contraindicated medical condition	n (%)
Other	n (%)

Table 3: Demographics – mITT Subjects

Characteristic	WR 279,396<br>n=xx	Paromomycin<br>n=xx	All Subjects<br>n=xx	p-value[a]
**Sex**				
Male, n (%)	xx (x.x)	xx (x.x)	xx (x.x)	0.xxx
Female, n (%)	xx (x.x)	xx (x.x)	xx (x.x)	
**Age**				
(years at date of consent)				
Mean (SD)	xx.x (x.xx)	xx.x (x.xx)	xx.x (x.xx)	0.xxx
Median	xx.x	xx.x	xx.x	
Range	xx-xx	xx-xx	xx-xx	
**Age Strata**				
Adults, n (%)	xx (x.x)	xx (x.x)	xx (x.x)	0.xxx
Children (12 to 17), n, (%)	xx (x.x)	xx (x.x)	xx (x.x)	
Children (<12), n (%)	xx (x.x)	xx (x.x)	xx (x.x)	
**Race**				
n (%)	xx	xx	xx	
Mestizo	xx (x.x)	xx (x.x)	xx (x.x)	0.xxx
Other	xx (x.x)	xx (x.x)	xx (x.x)	
**Ethnicity**				
n (%)	xx	xx	xx	
Hispanic or Latino	xx (x.x)	xx (x.x)	xx (x.x)	0.xxx
Not Hispanic or Latino	xx (x.x)	xx (x.x)	xx (x.x)	
Not given	xx (x.x)	xx (x.x)	xx (x.x)	

[a] p-value by Fisher's Exact Test for discontinuous discrete variables, chi-square test for categorical, and t-test or appropriate non-parametric test for continuous variables or Wilcoxon rank sum test for data that are skewed.
<Program Name: Time and Date>

Table 4: Demographics – Evaluable Subjects

Characteristic	WR 279,396<br>n=xx	Paromomycin<br>n=xx	All Subjects<br>n=xx	p-value[a]
**Sex**				
Male, n (%)	xx (x.x)	xx (x.x)	xx (x.x)	0.xxx
Female, n (%)	xx (x.x)	xx (x.x)	xx (x.x)	
**Age**				
(years at date of consent)				
Mean (SD)	xx.x (x.xx)	xx.x (x.xx)	xx.x (x.xx)	0.xxx
Median	xx.x	xx.x	xx.x	
Range	xx-xx	xx-xx	xx-xx	
**Age Strata**				
Adults, n (%)	xx (x.x)	xx (x.x)	xx (x.x)	0.xxx
Children (12 to 17), n, (%)	xx (x.x)	xx (x.x)	xx (x.x)	
Children (<12), n (%)	xx (x.x)	xx (x.x)	xx (x.x)	
**Race**				
n (%)	xx	xx	xx	
Mestizo	xx (x.x)	xx (x.x)	xx (x.x)	0.xxx
Other	xx (x.x)	xx (x.x)	xx (x.x)	
**Ethnicity**				
n (%)	xx	xx	xx	
Hispanic or Latino	xx (x.x)	xx (x.x)	xx (x.x)	0.xxx
Not Hispanic or Latino	xx (x.x)	xx (x.x)	xx (x.x)	
Not given	xx (x.x)	xx (x.x)	xx (x.x)	

[a] p-value by Fisher's Exact Test for discontinuous discrete variables, chi-square test for categorical, and t-test or appropriate non-parametric test for continuous variables or Wilcoxon rank sum test for data that are skewed.
<Program Name: Time and Date>

Table 5: Demographics – *L panamensis* mITT Subjects

Characteristic	WR 279,396<br>n=xx	Paromomycin<br>n=xx	All Subjects<br>n=xx	p-value[a]
**Sex**				
Male, n (%)	xx (x.x)	xx (x.x)	xx (x.x)	0.xxx
Female, n (%)	xx (x.x)	xx (x.x)	xx (x.x)	
**Age**				
(years at date of consent)				
Mean (SD)	xx.x (x.xx)	xx.x (x.xx)	xx.x (x.xx)	0.xxx
Median	xx.x	xx.x	xx.x	
Range	xx-xx	xx-xx	xx-xx	
**Age Strata**				
Adults, n (%)	xx (x.x)	xx (x.x)	xx (x.x)	0.xxx
Children (12 to 17), n, (%)	xx (x.x)	xx (x.x)	xx (x.x)	
Children (<12), n (%)	xx (x.x)	xx (x.x)	xx (x.x)	
**Race**				
n (%)	xx	xx	xx	
Mestizo	xx (x.x)	xx (x.x)	xx (x.x)	0.xxx
Other	xx (x.x)	xx (x.x)	xx (x.x)	
**Ethnicity**				
n (%)	xx	xx	xx	
Hispanic or Latino	xx (x.x)	xx (x.x)	xx (x.x)	0.xxx
Not Hispanic or Latino	xx (x.x)	xx (x.x)	xx (x.x)	
Not given	xx (x.x)	xx (x.x)	xx (x.x)	

[a] p-value by Fisher's Exact Test for discontinuous discrete variables, chi-square test for categorical, and t-test or appropriate non-parametric test for continuous variables or Wilcoxon rank sum test for data that are skewed.
<Program Name: Time and Date>

Table 6: Baseline Lesions Characteristics – mITT Subjects

Characteristic	WR 279,396<br>n=xx	Paromomycin<br>n=xx	All Subjects<br>n=xx	p-value[a]
**Total Number of Lesions per subject**				
Mean (SD)	xx.x (x.xx)	xx.x (x.xx)	xx.x (x.xx)	0.xxx
Median	xx.x	xx.x	xx.x	
Range	xx-xx	xx-xx	xx-xx	
**All Lesions Characteristics**				
Ulcerated, n (%)	xx (x.x)	xx (x.x)	xx (x.x)	0.xxx
Non-Ulcerated, n (%)	xx (x.x)	xx (x.x)	xx (x.x)	
Secondary Infection, n (%)	xx (x.x)	xx (x.x)	xx (x.x)	
**Index Lesion Ulceration Area (mm^2)**				
Mean (SD)	xx.x (x.xx)	xx.x (x.xx)	xx.x (x.xx)	0.xxx
Median	xx.x	xx.x	xx.x	
Range	xx-xx	xx-xx	xx-xx	
**All Lesions Area (mm^2)**				
Mean (SD)	xx.x (x.xx)	xx.x (x.xx)	xx.x (x.xx)	0.xxx
Median	xx.x	xx.x	xx.x	
Range	xx-xx	xx-xx	xx-xx	
**Body Sites, n (%)**				
Face	xx (xx.x)	xx (xx.x)	xx (xx.x)	
Arm	xx (xx.x)	xx (xx.x)	xx (xx.x)	
Upper Torso	xx (xx.x)	xx (xx.x)	xx (xx.x)	
Abdomen	xx (xx.x)	xx (xx.x)	xx (xx.x)	
Hand	xx (xx.x)	xx (xx.x)	xx (xx.x)	
Groin	xx (xx.x)	xx (xx.x)	xx (xx.x)	
Leg	xx (xx.x)	xx (xx.x)	xx (xx.x)	
Foot	xx (xx.x)	xx (xx.x)	xx (xx.x)	
Back of Head	xx (xx.x)	xx (xx.x)	xx (xx.x)	
Buttocks	xx (xx.x)	xx (xx.x)	xx (xx.x)	
Anus	xx (xx.x)	xx (xx.x)	xx (xx.x)	
**Infecting Species, n (%)**				
L panamensis	xx (xx.x)	xx (xx.x)	xx (xx.x)	0.xxx
L guyanensis	xx (xx.x)	xx (xx.x)	xx (xx.x)	
L braziliensis	xx (xx.x)	xx (xx.x)	xx (xx.x)	

[a] p-value by Fisher's Exact Test for discontinuous discrete variables, chi-square test for categorical, and t-test or appropriate non-parametric test for continuous variables or Wilcoxon rank sum test for data that are skewed.

Note: Maximum of 10 lesions allowed at baseline.

<Program Name: Time and Date>

Table 7: Baseline Lesions Characteristics – Evaluable Subjects

Characteristic	WR 279,396<br>n=xx	Paromomycin<br>n=xx	All Subjects<br>n=xx	p-value[a]
**Total Number of Lesions per subject**				
Mean (SD)	xx.x (x.xx)	xx.x (x.xx)	xx.x (x.xx)	0.xxx
Median	xx.x	xx.x	xx.x	
Range	xx-xx	xx-xx	xx-xx	
**All Lesions Characteristics**				
Ulcerated, n (%)	xx (x.x)	xx (x.x)	xx (x.x)	0.xxx
Non-Ulcerated, n (%)	xx (x.x)	xx (x.x)	xx (x.x)	
Secondary Infection, n (%)	xx (x.x)	xx (x.x)	xx (x.x)	
**Index Lesion Ulceration Area (mm^2)**				
Mean (SD)	xx.x (x.xx)	xx.x (x.xx)	xx.x (x.xx)	0.xxx
Median	xx.x	xx.x	xx.x	
Range	xx-xx	xx-xx	xx-xx	
**All Lesions Area (mm^2)**				
Mean (SD)	xx.x (x.xx)	xx.x (x.xx)	xx.x (x.xx)	0.xxx
Median	xx.x	xx.x	xx.x	
Range	xx-xx	xx-xx	xx-xx	
**Body Sites, n (%)**				
Face	xx (xx.x)	xx (xx.x)	xx (xx.x)	
Arm	xx (xx.x)	xx (xx.x)	xx (xx.x)	
Upper Torso	xx (xx.x)	xx (xx.x)	xx (xx.x)	
Abdomen	xx (xx.x)	xx (xx.x)	xx (xx.x)	
Hand	xx (xx.x)	xx (xx.x)	xx (xx.x)	
Groin	xx (xx.x)	xx (xx.x)	xx (xx.x)	
Leg	xx (xx.x)	xx (xx.x)	xx (xx.x)	
Foot	xx (xx.x)	xx (xx.x)	xx (xx.x)	
Back of Head	xx (xx.x)	xx (xx.x)	xx (xx.x)	
Buttocks	xx (xx.x)	xx (xx.x)	xx (xx.x)	
Anus	xx (xx.x)	xx (xx.x)	xx (xx.x)	
**Infecting Species, n (%)**				
L panamensis	xx (xx.x)	xx (xx.x)	xx (xx.x)	0.xxx
L guyanensis	xx (xx.x)	xx (xx.x)	xx (xx.x)	
L braziliensis	xx (xx.x)	xx (xx.x)	xx (xx.x)	

[a] p-value by Fisher's Exact Test for discontinuous discrete variables, chi-square test for categorical, and t-test or appropriate non-parametric test for continuous variables or Wilcoxon rank sum test for data that are skewed.
Note: Maximum of 10 lesions allowed at baseline.
<Program Name: Time and Date>

Table 8: Baseline Leishmaniasis History – mITT Subjects

Study Drug	WR 279,396<br>n=xx	Paromomycin<br>n=xx	All Subjects<br>n=xx	p-value[a]
**Days before treatment that lesions were first noticed**				
n	xx	xx	xx	
Mean (SD)	xx.x (x.xx)	xx.x (x.xx)	xx.x (x.xx)	0.xxx
Median	xx.x	xx.x	xx.x	
Range	xx-xx	xx-xx	xx-xx	

[a] P-value by T-test or appropriate non-parametric test for continuous variables or Wilcoxon rank sum test for data that are skewed.
<Program Name: Time and Date>

Table 9: Baseline Leishmaniasis History – Evaluable Subjects

Study Drug	WR 279,396<br>n=xx	Paromomycin<br>n=xx	All Subjects<br>n=xx	p-value[a]
**Days before treatment that lesions were first noticed**				
n	xx	xx	xx	
Mean (SD)	xx.x (x.xx)	xx.x (x.xx)	xx.x (x.xx)	0.xxx
Median	xx.x	xx.x	xx.x	
Range	xx-xx	xx-xx	xx-xx	

[a] p-value by t-test or appropriate non-parametric test for continuous variables or Wilcoxon rank sum test for data that are skewed.
<Program Name: Time and Date>

Table 10: Baseline Leishmaniasis History – *L panamensis* mITT Subjects

Study Drug	WR 279,396<br>n=xx	Paromomycin<br>n=xx	All Subjects<br>n=xx	p-value[a]
**Days before treatment that lesions were first noticed**				
n	xx	xx	xx	
Mean (SD)	xx.x (x.xx)	xx.x (x.xx)	xx.x (x.xx)	0.xxx
Median	xx.x	xx.x	xx.x	
Range	xx-xx	xx-xx	xx-xx	

[a] p-value by t-test or appropriate non-parametric test for continuous variables or Wilcoxon rank sum test for data that are skewed.
<Program Name: Time and Date>

Table 11: Summary of Medical History

Medical History Preferred Term	WR 279,396<br>n=xx	Paromomycin<br>n=xx
Preferred Term No. 1	n (%)	n (%)
Preferred Term No. 2	n (%)	n (%)

Table 12: Baseline Vital Signs – mITT Subjects

Study Drug	WR 279,396<br>n=xx	Paromomycin<br>n=xx	All Subjects<br>n=xx	p-value[a]
**Temperature (C^{o})**				
Mean (SD)	xx.x (x.xx)	xx.x (x.xx)	xx.x (x.xx)	0.xxx
Median	xx.x	xx.x	xx.x	
Range	xx-xx	xx-xx	xx-xx	
**Systolic Blood Pressure (mm Hg)**				
Mean (SD)	xx.x (x.xx)	xx.x (x.xx)	xx.x (x.xx)	0.xxx
Median	xx.x	xx.x	xx.x	
Range	xx-xxx	xx-xxx	xx-xxx	
**Diastolic Blood Pressure (mm Hg)**				
Mean (SD)	xx.x (x.xx)	xx.x (x.xx)	xx.x (x.xx)	0.xxx
Median	xx.x	xx.x	xx.x	
Range	xx-xxx	xx-xxx	xx-xxx	
**Heart rate (beats/min)**				
Mean (SD)	xx.x (x.xx)	xx.x (x.xx)	xx.x (x.xx)	0.xxx
Median	xx.x	xx.x	xx.x	
Range	xx-xxx	xx-xxx	xx-xxx	

[a] p-value by t-test or appropriate non-parametric test for continuous variables or Wilcoxon rank sum test for data that are skewed.

<Program Name: Time and Date>

Table 13: Baseline Vital Signs – Evaluable Subjects

Study Drug	WR 279,396<br>n=xx	Paromomycin<br>n=xx	All Subjects<br>n=xx	p-value[a]
**Temperature (C^o)**				
Mean (SD)	xx.x (x.xx)	xx.x (x.xx)	xx.x (x.xx)	0.xxx
Median	xx.x	xx.x	xx.x	
Range	xx-xx	xx-xx	xx-xx	
**Systolic Blood Pressure (mmHg)**				
Mean (SD)	xx.x (x.xx)	xx.x (x.xx)	xx.x (x.xx)	0.xxx
Median	xx.x	xx.x	xx.x	
Range	xx-xxx	xx-xxx	xx-xxx	
**Diastolic Blood Pressure (mmHg)**				
Mean (SD)	xx.x (x.xx)	xx.x (x.xx)	xx.x (x.xx)	0.xxx
Median	xx.x	xx.x	xx.x	
Range	xx-xxx	xx-xxx	xx-xxx	
**Heart rate (beats/min)**				
Mean (SD)	xx.x (x.xx)	xx.x (x.xx)	xx.x (x.xx)	0.xxx
Median	xx.x	xx.x	xx.x	
Range	xx-xxx	xx-xxx	xx-xxx	

[a] p-value by t-test or appropriate non-parametric test for continuous variables or Wilcoxon rank sum test for data that are skewed.

<Program Name: Time and Date>

Table 14: Protocol Compliance – mITT Subjects

Visit Attended	WR 279,396 n (%) n=xx	Paromomycin n (%) n=xx
Day 1		
Day 2	xx (x.x)	xx (x.x)
…….daily to Day 20, 35, 49, 63, 100,	xx (x.x)	xx (x.x)
168	xx (x.x)	xx (x.x)

<Program Name: Time and Date>

Table 15: Baseline Parasitology – mITT Subjects

	WR 279,396 n=xx	Paromomycin n=xx	All Subjects n=xx	p-value[a]
**Microscopic Evaluation**				
Number of lesions tested	xx	xx	xx	
N (% positive for leishmaniasis)	xx (x.x)	xx (x.x)	xx (x.x)	0.xxx
**Culture Evaluation**				
Number of lesions tested	xx	xx	xx	
N (% positive for leishmaniasis)	xx (x.x)	xx (x.x)	xx (x.x)	0.xxx
**PCR/RFLP Speciation**				
**Number identified**	xx	xx	xx	
L panamensis n (%)	xx (x.x)	xx (x.x)	xx (x.x)	0.xxx
L guyanensis n (%)	xx (x.x)	xx (x.x)	xx (x.x)	
L braziliensis n (%)	xx (x.x)	xx (x.x)	xx (x.x)	
Other n (%)	xx (x.x)	xx (x.x)	xx (x.x)	
**PCR/RFLP Speciation with Missing Added**				
L panamensis n (%)	xx (x.x)	xx (x.x)	xx (x.x)	0.xxx
L guyanensis n (%)	xx (x.x)	xx (x.x)	xx (x.x)	
L braziliensis n (%)	xx (x.x)	xx (x.x)	xx (x.x)	
Other n (%)	xx (x.x)	xx (x.x)	xx (x.x)	
**Isoenzyme Speciation**				
**Number identified**	xx	xx	xx	
L panamensis n (%)	xx (x.x)	xx (x.x)	xx (x.x)	0.xxx
L guyanensis n (%)	xx (x.x)	xx (x.x)	xx (x.x)	
L braziliensis n (%)	xx (x.x)	xx (x.x)	xx (x.x)	
Other n (%)	xx (x.x)	xx (x.x)	xx (x.x)	

[a] p-value by Fisher's Exact Test for discontinuous discrete variables and chi-square test for categorical.

<Program Name: Time and Date>

Table 16: Baseline Parasitology – Evaluable Subjects

	WR 279,396<br>n=xx	Paromomycin<br>n=xx	All Subjects<br>n=xx	p-value[a]
**Microscopic Evaluation**				
Number of lesions tested	xx	xx	xx	
N (% positive for leishmaniasis)	xx (x.x)	xx (x.x)	xx (x.x)	0.xxx
**Culture Evaluation**				
Number of lesions tested	xx	xx	xx	
N (% positive for leishmaniasis)	xx (x.x)	xx (x.x)	xx (x.x)	0.xxx
**PCR/RFLP Speciation**				
**Number identified**	xx	xx	xx	
L panamensis n (%)	xx (x.x)	xx (x.x)	xx (x.x)	0.xxx
L guyanensis n (%)	xx (x.x)	xx (x.x)	xx (x.x)	
L braziliensis n (%)	xx (x.x)	xx (x.x)	xx (x.x)	
Other n (%)	xx (x.x)	xx (x.x)	xx (x.x)	
**PCR/RFLP Speciation with Missing Added**				
L panamensis n (%)	xx (x.x)	xx (x.x)	xx (x.x)	0.xxx
L guyanensis n (%)	xx (x.x)	xx (x.x)	xx (x.x)	
L braziliensis n (%)	xx (x.x)	xx (x.x)	xx (x.x)	
Other n (%)	xx (x.x)	xx (x.x)	xx (x.x)	
**Isoenzyme Speciation**				
**Number identified**	xx	xx	xx	
L panamensis n (%)	xx (x.x)	xx (x.x)	xx (x.x)	0.xxx
L guyanensis n (%)	xx (x.x)	xx (x.x)	xx (x.x)	
L braziliensis n (%)	xx (x.x)	xx (x.x)	xx (x.x)	
Other n (%)	xx (x.x)	xx (x.x)	xx (x.x)	

[a] p-value by Fisher's Exact Test for discontinuous discrete variables and chi-square test for categorical.

<Program Name: Time and Date>

Table 17: Drug Exposure During Days 1 to 20 – mITT Subjects

**Study Drug**	**WR 279,396**<br>**n=xx**	**Paromomycin**<br>**n=xx**
**Days of Application**		
n	xx	xx
Mean (SD)	xx.x (x.xx)	xx.x (x.xx)
Median	xx.x	xx.x
Range	xx-xx	xx-xx
**Total Weight of Drug Applied (g)**		
n	xx	xx
Mean (SD)	xx.x (x.xx)	xx.x (x.xx)
Median	xx.x	xx.x
Range	xx-xx	xx-xx
**Average Total Daily Exposure (g)**		
n	xx	xx
Mean (SD)	xx.x (x.xx)	xx.x (x.xx)
Median	xx.x	xx.x
Range	xx-xx	xx-xx
**Average Total Daily Exposure by Lesion area (g/mm^2)**	xx	xx
n	xx.x (x.xx)	xx.x (x.xx)
Mean (SD)	xx.x	xx.x
Median	xx-xx	xx-xx
Range		

<Program Name: Time and Date>

Table 18: Drug Exposure During Days 1 to 20 – Evaluable Subjects

**Study Drug**	**WR 279,396**<br>**n=xx**	**Paromomycin**<br>**n=xx**
**Days of Application**		
n	xx	xx
Mean (SD)	xx.x (x.xx)	xx.x (x.xx)
Median	xx.x	xx.x
Range	xx-xx	xx-xx
**Total Weight of Drug Applied (g)**		
n	xx	xx
Mean (SD)	xx.x (x.xx)	xx.x (x.xx)
Median	xx.x	xx.x
Range	xx-xx	xx-xx
**Average Daily Exposure**		
n	xx	xx
Mean (SD)	xx.x (x.xx)	xx.x (x.xx)
Median	xx.x	xx.x
Range	xx-xx	xx-xx
**Average Total Daily Exposure by Lesion area (g/mm^2)**	xx	xx
n	xx.x (x.xx)	xx.x (x.xx)
Mean (SD)	xx.x	xx.x
Median	xx-xx	xx-xx
Range		

<Program Name: Time and Date>

Table 19: Drug Exposure During Treatment of New Lesions (Post Day 20) – mITT Subjects

	WR 279,396<br>n=xx	Paromomycin<br>n=xx
**Days of Application**		
Number of new lesions	xx	xx
Mean (SD)	xx.x (x.xx)	xx.x (x.xx)
Median	xx.x	xx.x
Range	xx-xx	xx-xx
**Total Weight of Drug Applied (g)**		
Number of new lesions	xx	xx
Mean (SD)	xx.x (x.xx)	xx.x (x.xx)
Median	xx.x	xx.x
Range	xx-xx	xx-xx

<Program Name: Time and Date>

Table 20: Drug Exposure During Treatment of New Lesions (Post Day 20) – Evaluable Subjects

	WR 279,396<br>n=xx	Paromomycin<br>n=xx
**Days of Application**		
Number of new lesions	xx	xx
Mean (SD)	xx.x (x.xx)	xx.x (x.xx)
Median	xx.x	xx.x
Range	xx-xx	xx-xx
**Total Weight of Drug Applied (g)**		
Number of new lesions	xx	xx
Mean (SD)	xx.x (x.xx)	xx.x (x.xx)
Median	xx.x	xx.x
Range	xx-xx	xx-xx

<Program Name: Time and Date>

Table 21: Numbers and Proportion of Subjects Meeting Criteria for Final Clinical Cure of Index Lesions – mITT Subjects

	WR 279,396 n=xx	Paromomycin n=xx	p-value[a]
**Clinical Cure of Index Lesion**			
n (%)	xx (x.x)	xx (x.x)	0.xxx
Difference	xx		
95% CI[b]	(xxx.x-xx.x)		

[a] Uncorrected chi-square test (2-sided)
[b] CI=Confidence interval
<Program Name: Time and Date>

Table 22: Final Clinical Cure of Index Lesion Logistic Regression – mITT Subjects

Parameter		Estimate	SE	Wald Chi-Square	p-value
Intercept		xx.xxx	xx.xxx	xx.xxx	0.xxx
Treatment	WR 279,396	xx.xxx	xx.xxx	xx.xxx	0.xxx
Covariate		xx.xxx	xx.xxx	xx.xxx	0.xxx

Table 23: Numbers and Proportion of Subjects Meeting for Final Clinical Cure of Index Lesions – Evaluable Subjects

	WR 279,396 n=xx	Paromomycin n=xx	p-value[a]
**Clinical Cure of Index Lesion**			
n (%)	xx (x.x)	xx (x.x)	x.xxx
Difference	xx		
95% CI[b]	(xxx.x-xx.x)		

[a] Uncorrected chi-square test (2-sided)
[b] CI=Confidence interval
<Program Name: Time and Date>

Table 24: Final Clinical Cure of Index Lesion Logistic Regression – Evaluable Subjects

Parameter		Estimate	SE	Wald Chi-Square	p-value
Intercept		xx.xxx	xx.xxx	xx.xxx	0.xxx
Treatment	WR 279,396	xx.xxx	xx.xxx	xx.xxx	0.xxx
Covariate		xx.xxx	xx.xxx	xx.xxx	0.xxx

Table 25: Numbers and Proportion of Subjects Meeting Criteria for Final Clinical Cure of All Lesions – mITT Subjects

	WR 279,396<br>n=xx	Paromomycin<br>n=xx	P-value[a]
**Clinical Cure of All Lesions**			
n (%)	xx (x.x)	xx (x.x)	x.xxx
Difference	xx		
95% CI[b]	(xxx.x-xx.x)		

[a] Uncorrected chi-square test (2-sided)
[b] CI=Confidence interval
<Program Name: Time and Date>

Table 26: Numbers and Proportion of Subjects Meeting Criteria for Final Clinical Cure of All Lesions – Evaluable Subjects

	WR 279,396<br>n=xx	Paromomycin<br>n=xx	P-value[a]
**Clinical Cure of All Lesions**			
n (%)	xx (x.x)	xx (x.x)	x.xxx
Difference	xx		
95% CI[b]	(xxx.x-xx.x)		

[a] Uncorrected chi-square test (2-sided)
[b] CI=Confidence interval
<Program Name: Time and Date>

Table 27: Area of Ulceration and Change from Baseline of the Index Lesion – mITT Subjects

Study Day<br>Treatment	n	Mean	SD[a]	Median[a]	Max[a]	Min[a]
**Day 1**						
WR 279,396	xx	xx.x	xx.x	xx.x	xx.x	xx.x
Paromomycin	xx	xx.x	xx.x	xx.x	xx.x	xx.x
**Day 20**						
WR 279,396	xx	xx.x	xx.x	xx.x	xx.x	xx.x
Paromomycin	xx	xx.x	xx.x	xx.x	xx.x	xx.x
CFB[b]						
WR 279,396	xx	xx.x	xx.x	xx.x	xx.x	xx.x
Paromomycin	xx	xx.x	xx.x	xx.x	xx.x	xx.x
**Day 35**						
WR 279,396	xx	xx.x	xx.x	xx.x	xx.x	xx.x
Paromomycin	xx	xx.x	xx.x	xx.x	xx.x	xx.x
CFB						
WR 279,396	xx	xx.x	xx.x	xx.x	xx.x	xx.x
Paromomycin	xx	xx.x	xx.x	xx.x	xx.x	xx.x
**Day 49**						
WR 279,396	xx	xx.x	xx.x	xx.x	xx.x	xx.x
Paromomycin	xx	xx.x	xx.x	xx.x	xx.x	xx.x
CFB						
WR 279,396	xx	xx.x	xx.x	xx.x	xx.x	xx.x
Paromomycin	xx	xx.x	xx.x	xx.x	xx.x	xx.x
**Day 63**						
WR 279,396	xx	xx.x	xx.x	xx.x	xx.x	xx.x
Paromomycin	xx	xx.x	xx.x	xx.x	xx.x	xx.x
CFB						
WR 279,396	xx	xx.x	xx.x	xx.x	xx.x	xx.x
Paromomycin	xx	xx.x	xx.x	xx.x	xx.x	xx.x
**Day 100**						
WR 279,396	xx	xx.x	xx.x	xx.x	xx.x	xx.x
Paromomycin	xx	xx.x	xx.x	xx.x	xx.x	xx.x
CFB						
WR 279,396	xx	xx.x	xx.x	xx.x	xx.x	xx.x
Paromomycin	xx	xx.x	xx.x	xx.x	xx.x	xx.x
**Day 168**						
WR 279,396	xx	xx.x	xx.x	xx.x	xx.x	xx.x
Paromomycin	xx	xx.x	xx.x	xx.x	xx.x	xx.x
CFB						
WR 279,396	xx	xx.x	xx.x	xx.x	xx.x	xx.x
Paromomycin	xx	xx.x	xx.x	xx.x	xx.x	xx.x

[a]SD=Standard Deviation; Med=median; Max=maximum; Min=minimum

[b]CFB=Change from baseline

<Program Name: Time and Date>

Table 28: Area of Ulceration and Change from Baseline of the Index Lesion – Evaluable Subjects

Study Day<br>Treatment	n	Mean	SD[a]	Median[a]	Max[a]	Min[a]
**Day 1**						
WR 279,396	xx	xx.x	xx.x	xx.x	xx.x	xx.x
Paromomycin	xx	xx.x	xx.x	xx.x	xx.x	xx.x
**Day 20**						
WR 279,396	xx	xx.x	xx.x	xx.x	xx.x	xx.x
Paromomycin	xx	xx.x	xx.x	xx.x	xx.x	xx.x
CFB[b]						
WR 279,396	xx	xx.x	xx.x	xx.x	xx.x	xx.x
Paromomycin	xx	xx.x	xx.x	xx.x	xx.x	xx.x
**Day 35**						
WR 279,396	xx	xx.x	xx.x	xx.x	xx.x	xx.x
Paromomycin	xx	xx.x	xx.x	xx.x	xx.x	xx.x
CFB						
WR 279,396	xx	xx.x	xx.x	xx.x	xx.x	xx.x
Paromomycin	xx	xx.x	xx.x	xx.x	xx.x	xx.x
**Day 49**						
WR 279,396	xx	xx.x	xx.x	xx.x	xx.x	xx.x
Paromomycin	xx	xx.x	xx.x	xx.x	xx.x	xx.x
CFB						
WR 279,396	xx	xx.x	xx.x	xx.x	xx.x	xx.x
Paromomycin	xx	xx.x	xx.x	xx.x	xx.x	xx.x
**Day 63**						
WR 279,396	xx	xx.x	xx.x	xx.x	xx.x	xx.x
Paromomycin	xx	xx.x	xx.x	xx.x	xx.x	xx.x
CFB						
WR 279,396	xx	xx.x	xx.x	xx.x	xx.x	xx.x
Paromomycin	xx	xx.x	xx.x	xx.x	xx.x	xx.x
**Day 100**						
WR 279,396	xx	xx.x	xx.x	xx.x	xx.x	xx.x
Paromomycin	xx	xx.x	xx.x	xx.x	xx.x	xx.x
CFB						
WR 279,396	xx	xx.x	xx.x	xx.x	xx.x	xx.x
Paromomycin	xx	xx.x	xx.x	xx.x	xx.x	xx.x
**Day 168**						
WR 279,396	xx	xx.x	xx.x	xx.x	xx.x	xx.x
Paromomycin	xx	xx.x	xx.x	xx.x	xx.x	xx.x
CFB						
WR 279,396	xx	xx.x	xx.x	xx.x	xx.x	xx.x
Paromomycin	xx	xx.x	xx.x	xx.x	xx.x	xx.x

[a]SD=Standard Deviation; Med=median; Max=maximum; Min=minimum

[b]CFB=Change from baseline

<Program Name: Time and Date>

Table 29: Area of All Lesions and Change from Baseline – mITT Subjects

Study Day<br>Treatment	n	Mean	SD[a]	Median[a]	Max[a]	Min[a]
**Day 1**						
WR 279,396	xx	xx.x	xx.x	xx.x	xx.x	xx.x
Paromomycin	xx	xx.x	xx.x	xx.x	xx.x	xx.x
**Day 20**						
WR 279,396	xx	xx.x	xx.x	xx.x	xx.x	xx.x
Paromomycin	xx	xx.x	xx.x	xx.x	xx.x	xx.x
CFB[b]						
WR 279,396	xx	xx.x	xx.x	xx.x	xx.x	xx.x
Paromomycin	xx	xx.x	xx.x	xx.x	xx.x	xx.x
**Day 35**						
WR 279,396	xx	xx.x	xx.x	xx.x	xx.x	xx.x
Paromomycin	xx	xx.x	xx.x	xx.x	xx.x	xx.x
CFB						
WR 279,396	xx	xx.x	xx.x	xx.x	xx.x	xx.x
Paromomycin	xx	xx.x	xx.x	xx.x	xx.x	xx.x
**Day 49**						
WR 279,396	xx	xx.x	xx.x	xx.x	xx.x	xx.x
Paromomycin	xx	xx.x	xx.x	xx.x	xx.x	xx.x
CFB						
WR 279,396	xx	xx.x	xx.x	xx.x	xx.x	xx.x
Paromomycin	xx	xx.x	xx.x	xx.x	xx.x	xx.x
**Day 63**						
WR 279,396	xx	xx.x	xx.x	xx.x	xx.x	xx.x
Paromomycin	xx	xx.x	xx.x	xx.x	xx.x	xx.x
CFB						
WR 279,396	xx	xx.x	xx.x	xx.x	xx.x	xx.x
Paromomycin	xx	xx.x	xx.x	xx.x	xx.x	xx.x
**Day 100**						
WR 279,396	xx	xx.x	xx.x	xx.x	xx.x	xx.x
Paromomycin	xx	xx.x	xx.x	xx.x	xx.x	xx.x
CFB						
WR 279,396	xx	xx.x	xx.x	xx.x	xx.x	xx.x
Paromomycin	xx	xx.x	xx.x	xx.x	xx.x	xx.x
**Day 168**						
WR 279,396	xx	xx.x	xx.x	xx.x	xx.x	xx.x
Paromomycin	xx	xx.x	xx.x	xx.x	xx.x	xx.x
CFB						
WR 279,396	xx	xx.x	xx.x	xx.x	xx.x	xx.x
Paromomycin	xx	xx.x	xx.x	xx.x	xx.x	xx.x

[a]SD=Standard Deviation; Max=maximum; Min=minimum

[b]CFB=Change from baseline

<Program Name: Time and Date>

Table 30: Area of All Lesions and Change from Baseline – Evaluable Subjects

Study Day<br>Treatment	n	Mean	SD[a]	Median[a]	Max[a]	Min[a]
**Day 1**						
WR 279,396	xx	xx.x	xx.x	xx.x	xx.x	xx.x
Paromomycin	xx	xx.x	xx.x	xx.x	xx.x	xx.x
**Day 20**						
WR 279,396	xx	xx.x	xx.x	xx.x	xx.x	xx.x
Paromomycin	xx	xx.x	xx.x	xx.x	xx.x	xx.x
CFB[b]						
WR 279,396	xx	xx.x	xx.x	xx.x	xx.x	xx.x
Paromomycin	xx	xx.x	xx.x	xx.x	xx.x	xx.x
**Day 35**						
WR 279,396	xx	xx.x	xx.x	xx.x	xx.x	xx.x
Paromomycin	xx	xx.x	xx.x	xx.x	xx.x	xx.x
CFB						
WR 279,396	xx	xx.x	xx.x	xx.x	xx.x	xx.x
Paromomycin	xx	xx.x	xx.x	xx.x	xx.x	xx.x
**Day 49**						
WR 279,396	xx	xx.x	xx.x	xx.x	xx.x	xx.x
Paromomycin	xx	xx.x	xx.x	xx.x	xx.x	xx.x
CFB						
WR 279,396	xx	xx.x	xx.x	xx.x	xx.x	xx.x
Paromomycin	xx	xx.x	xx.x	xx.x	xx.x	xx.x
**Day 63**						
WR 279,396	xx	xx.x	xx.x	xx.x	xx.x	xx.x
Paromomycin	xx	xx.x	xx.x	xx.x	xx.x	xx.x
CFB						
WR 279,396	xx	xx.x	xx.x	xx.x	xx.x	xx.x
Paromomycin	xx	xx.x	xx.x	xx.x	xx.x	xx.x
**Day 100**						
WR 279,396	xx	xx.x	xx.x	xx.x	xx.x	xx.x
Paromomycin	xx	xx.x	xx.x	xx.x	xx.x	xx.x
CFB						
WR 279,396	xx	xx.x	xx.x	xx.x	xx.x	xx.x
Paromomycin	xx	xx.x	xx.x	xx.x	xx.x	xx.x
**Day 168**						
WR 279,396	xx	xx.x	xx.x	xx.x	xx.x	xx.x
Paromomycin	xx	xx.x	xx.x	xx.x	xx.x	xx.x
CFB						
WR 279,396	xx	xx.x	xx.x	xx.x	xx.x	xx.x
Paromomycin	xx	xx.x	xx.x	xx.x	xx.x	xx.x

[a]SD=Standard Deviation; Max=maximum; Min=minimum

[b]CFB=Change from baseline

<Program Name: Time and Date>

Table 31: Numbers and Proportion of Index Lesions Meeting Criteria for Clinical Cure During the Study – mITT Subjects

Study Day	1	20	35	49	63	100	168
**Study Drug**							
**WR 279,396 (n=xx)**							
n	xx.x	xx.x	xx.x	xx.x	xx.x	xx.x	xx.x
(%)	(x.xx)	(x.xx)	(x.xx)	(x.xx)	(x.xx)	(x.xx)	(x.xx)
**Paromomycin (n=xx)**							
n	xx.x	xx.x	xx.x	xx.x	xx.x	xx.x	xx.x
(%)	(x.xx)	(x.xx)	(x.xx)	(x.xx)	(x.xx)	(x.xx)	(x.xx)

<Program Name: Time and Date>

Table 32: Numbers and Proportion of Index Lesions Meeting Criteria for Clinical Cure Throughout the Study – Evaluable Subjects

Study Day	1	20	35	49	63	100	168
**Study Drug**							
**WR 279,396 (n=xx)**							
n	xx.x	xx.x	xx.x	xx.x	xx.x	xx.x	xx.x
(%)	(x.xx)	(x.xx)	(x.xx)	(x.xx)	(x.xx)	(x.xx)	(x.xx)
**Paromomycin (n=xx)**							
n	xx.x	xx.x	xx.x	xx.x	xx.x	xx.x	xx.x
(%)	(x.xx)	(x.xx)	(x.xx)	(x.xx)	(x.xx)	(x.xx)	(x.xx)

<Program Name: Time and Date>

Table 33: Generalized Estimating Equations of Index Lesions Meeting Criteria for Clinical Cure Across Time – mITT Subjects

Parameter		Estimate	SE	Wald Chi-Square	p-value
Intercept		xx.xxx	xx.xxx	xx.xxx	0.xxx
Treatment	WR 279,396	xx.xxx	xx.xxx	xx.xxx	0.xxx
Covariate		xx.xxx	xx.xxx	xx.xxx	0.xxx

<Program Name: Time and Date>

Table 34: Generalized Estimating Equations of Index Lesions Meeting Criteria for Clinical Cure Across Time – mITT Subjects

Parameter		Estimate	SE	Wald Chi-Square	p-value
Intercept		xx.xxx	xx.xxx	xx.xxx	0.xxx
Treatment	WR 279,396	xx.xxx	xx.xxx	xx.xxx	0.xxx
Covariate		xx.xxx	xx.xxx	xx.xxx	0.xxx

<Program Name: Time and Date>

Table 35: Numbers and Proportion of All Lesions Meeting Criteria for Clinical Cure During the Study – mITT Subjects

Study Day	1	20	35	49	63	100	168
**Study Drug**							
**WR 279,396 (n=xx)**							
n	xx.x	xx.x	xx.x	xx.x	xx.x	xx.x	xx.x
(%)	(x.xx)	(x.xx)	(x.xx)	(x.xx)	(x.xx)	(x.xx)	(x.xx)
**Paromomycin (n=xx)**							
n	xx.x	xx.x	xx.x	xx.x	xx.x	xx.x	xx.x
(%)	(x.xx)	(x.xx)	(x.xx)	(x.xx)	(x.xx)	(x.xx)	(x.xx)

<Program Name: Time and Date>

Table 36: Numbers and Proportion of All Lesions Meeting Criteria for Clinical Cure During the Study – Evaluable Subjects

Study Day	1	20	35	49	63	100	168
**Study Drug**							
**WR 279,396 (n=xx)**							
n	xx.x	xx.x	xx.x	xx.x	xx.x	xx.x	xx.x
(%)	(x.xx)	(x.xx)	(x.xx)	(x.xx)	(x.xx)	(x.xx)	(x.xx)
**Paromomycin (n=xx)**							
n	xx.x	xx.x	xx.x	xx.x	xx.x	xx.x	xx.x
(%)	(x.xx)	(x.xx)	(x.xx)	(x.xx)	(x.xx)	(x.xx)	(x.xx)

<Program Name: Time and Date>

Table 37: Generalized Estimating Equations of All Lesions Meeting Criteria for Clinical Cure Across Time – mITT Subjects

Parameter		Estimate	SE	Wald Chi-Square	p-value
Intercept		xx.xxx	xx.xxx	xx.xxx	0.xxx
Treatment	WR 279,396	xx.xxx	xx.xxx	xx.xxx	0.xxx
Covariate		xx.xxx	xx.xxx	xx.xxx	0.xxx

<Program Name: Time and Date>

Table 38: Generalized Estimating Equations of All Lesions Meeting Criteria for Clinical Cure Across Time – mITT Subjects

Parameter		Estimate	SE	Wald Chi-Square	p-value
Intercept		xx.xxx	xx.xxx	xx.xxx	0.xxx
Treatment	WR 279,396	xx.xxx	xx.xxx	xx.xxx	0.xxx
Covariate		xx.xxx	xx.xxx	xx.xxx	0.xxx

<Program Name: Time and Date>

Table 39: Median Time to Initial Clinical Cure of Index Lesions – mITT Subjects

	WR 279,396<br>n=xx	Paromomycin<br>n=xx	p-value[a]
**Median Time to Initial Clinical Cure**			
Median	xx	xx	0.xxx
95% CI	(xx, xx)	(xx, xx)	

[a]p-value from log-rank test

<Program Name: Time and Date>

Table 40: Median Time to Initial Clinical Cure of Index Lesions – Evaluable Subjects

	WR 279,396<br>n=xx	Paromomycin<br>n=xx	p-value[a]
**Median Time to Initial Clinical Cure**			
Median	xx	xx	0.xxx
95% CI	(xx, xx)	(xx, xx)	

[a]p-value from log-rank test

<Program Name: Time and Date>

Table 41: Numbers and Proportion of Subjects with Clinical Signs of Infection of the Index Lesion – mITT Subjects

Study Day	1	20	35	49	63	100	168
**Study Drug**							
**WR 279,396 (n=xx)**							
n	xx.x	xx.x	xx.x	xx.x	xx.x	xx.x	xx.x
(%)	(x.xx)	(x.xx)	(x.xx)	(x.xx)	(x.xx)	(x.xx)	(x.xx)
**Paromomycin (n=xx)**							
n	xx.x	xx.x	xx.x	xx.x	xx.x	xx.x	xx.x
(%)	(x.xx)	(x.xx)	(x.xx)	(x.xx)	(x.xx)	(x.xx)	(x.xx)

<Program Name: Time and Date>

Table 42: Numbers and Proportion of Subjects with Clinical Signs of Infection of the Index Lesion – Evaluable Subjects

Study Day	1	20	35	49	63	100	168
**Study Drug**							
**WR 279,396 (n=xx)**							
n	xx.x	xx.x	xx.x	xx.x	xx.x	xx.x	xx.x
(%)	(x.xx)	(x.xx)	(x.xx)	(x.xx)	(x.xx)	(x.xx)	(x.xx)
**Paromomycin (n=xx)**							
n	xx.x	xx.x	xx.x	xx.x	xx.x	xx.x	xx.x
(%)	(x.xx)	(x.xx)	(x.xx)	(x.xx)	(x.xx)	(x.xx)	(x.xx)

<Program Name: Time and Date>

Table 43: Numbers and Proportion of Subjects with Clinical Signs of Infection of All Lesions – mITT Subjects

Study Day	1	20	35	49	63	100	168
**Study Drug**							
**WR 279,396 (n=xx)**							
n	xx.x	xx.x	xx.x	xx.x	xx.x	xx.x	xx.x
(%)	(x.xx)	(x.xx)	(x.xx)	(x.xx)	(x.xx)	(x.xx)	(x.xx)
**Paromomycin (n=xx)**							
n	xx.x	xx.x	xx.x	xx.x	xx.x	xx.x	xx.x
(%)	(x.xx)	(x.xx)	(x.xx)	(x.xx)	(x.xx)	(x.xx)	(x.xx)

<Program Name: Time and Date>

Table 44: Numbers and Proportion of Subjects with Clinical Signs of Infection of All Lesions – Evaluable Subjects

Study Day	1	20	35	49	63	100	168
**Study Drug**							
**WR 279,396 (n=xx)**							
n	xx.x	xx.x	xx.x	xx.x	xx.x	xx.x	xx.x
(%)	(x.xx)	(x.xx)	(x.xx)	(x.xx)	(x.xx)	(x.xx)	(x.xx)
**Paromomycin (n=xx)**							
n	xx.x	xx.x	xx.x	xx.x	xx.x	xx.x	xx.x
(%)	(x.xx)	(x.xx)	(x.xx)	(x.xx)	(x.xx)	(x.xx)	(x.xx)

<Program Name: Time and Date>

Table 45: Numbers and Proportion of Subjects Meeting Criteria for Final Clinical Cure of Index Lesions: Gender by Treatment Group – mITT Subjects

	Males		Females	
	**WR 279,396 n=xx**	**Paromomycin n=xx**	**WR 279,396 n=xx**	**Paromomycin n=xx**
n (%)	xx (x.x)	xx (x.x)	xx (x.x)	xx (x.x)
Difference	xx		xx	
95% CI	(xxx.x-xx.x)		(xxx.x-xx.x)	
Difference p-value[a]	0.xxx		0.xxx	
Overall p-value[b]	0.xxx			

[a] uncorrected chi-square
[b] Cochran-Mantel-Haenszel test
<Program Name: Time and Date>

Table 46: Numbers and Proportion of Subjects Meeting Criteria for Final Clinical Cure of Index Lesions: Gender by Treatment Group – Evaluable Subjects

	Males		Females	
	**WR 279,396 n=xx**	**Paromomycin n=xx**	**WR 279,396 n=xx**	**Paromomycin n=xx**
n (%)	xx (x.x)	xx (x.x)	xx (x.x)	xx (x.x)
Difference	Xx		xx	
95% CI	(xxx.x-xx.x)		(xxx.x-xx.x)	
Difference p-value[a]	0.xxx		0.xxx	
Overall p-value[b]	0.xxx			

[a] uncorrected chi-square
[b] Cochran-Mantel-Haenszel test
<Program Name: Time and Date>

Table 47: Numbers and Proportion of Subjects Meeting Criteria for Final Clinical Cure of Index Lesions: Age Group by Treatment Group – mITT Subjects

Age	<12		12-17		Adult	
	**WR 279,396 n=xx**	**Paro-momycin n=xx**	**WR 279,396 n=xx**	**Paro-momycin n=xx**	**WR 279,396 n=xx**	**Paro-momycin n=xx**
n (%)	xx (x.x)	xx (x.x)	xx (x.x)	xx (x.x)	xx (x.x)	xx (x.x)
Difference<br>95% CI	xx<br>(xxx.x-xx.x)		xx<br>(xxx.x-xx.x)		xx<br>(xxx.x – xxx.x)	
Difference p-value[a]	0.xxx		0.xxx		0.xxx	
Overall p-value[b]	0.xxx					

[a] uncorrected chi-square
[b] Cochran-Mantel-Haenszel test
<Program Name: Time and Date>

Table 48: Numbers and Proportion of Subjects Meeting Criteria for Final Clinical Cure of Index Lesions: Age Group by Treatment Group – Evaluable Subjects

Age	<12		12-17		Adult	
	**WR 279,396 n=xx**	**Paro-momycin n=xx**	**WR 279,396 n=xx**	**Paro-momycin n=xx**	**WR 279,396 n=xx**	**Paro-momycin n=xx**
n (%)	xx (x.x)	xx (x.x)	xx (x.x)	xx (x.x)	xx (x.x)	xx (x.x)
Difference<br>95% CI	xx<br>(xxx.x-xx.x)		xx<br>(xxx.x-xx.x)		xx<br>(xxx.x – xxx.x)	
Difference p-value[a]	0.xxx		0.xxx		0.xxx	
Overall p-value[b]	0.xxx					

[a] uncorrected chi-square
[b] Cochran-Mantel-Haenszel test
<Program Name: Time and Date>

Table 49: Numbers and Proportion of Subjects Meeting Criteria for Final Clinical Cure of Index Lesions: Clinical Signs of Secondary Infection at Baseline by Treatment Group – mITT Subjects

	No Baseline Secondary Infection		Baseline Secondary Infection	
	**WR 279,396 n=xx**	**Paromomycin n=xx**	**WR 279,396 n=xx**	**Paromomycin n=xx**
n (%)	xx (x.x)	xx (x.x)	xx (x.x)	xx (x.x)
Difference<br>95% CI	xx<br>(xxx.x-xx.x)		xx<br>(xxx.x-xx.x)	
Difference p-value[a]	0.xxx		0.xxx	
Overall p-value[b]	0.xxx			

[a] uncorrected chi-square
[b] Cochran-Mantel-Haenszel test
<Program Name: Time and Date>

Table 50: Numbers and Proportion of Subjects Meeting Criteria for Final Clinical Cure of Index Lesions: Clinical Signs of Secondary Infection at Baseline by Treatment Group – Evaluable Subjects

	No Baseline Secondary Infection		Baseline Secondary Infection	
	**WR 279,396 n=xx**	**Paromomycin n=xx**	**WR 279,396 n=xx**	**Paromomycin n=xx**
n (%)	xx (x.x)	xx (x.x)	xx (x.x)	xx (x.x)
Difference<br>95% CI	xx<br>(xxx.x-xx.x)		xx<br>(xxx.x-xx.x)	
Difference p-value[a]	0.xxx		0.xxx	
Overall p-value[b]	0.xxx			

[a] uncorrected chi-square
[b] Cochran-Mantel-Haenszel test
<Program Name: Time and Date>

Table 51: Numbers and Proportion of Subjects Meeting Criteria for Final Clinical Cure of Index Lesions by Infecting Species Between Groups – mITT Subjects

	WR 279,396<br>n=xx		Paromomycin<br>n=xx
L panamensis			
n (%)	xx (x.x)		xx (x.x)
Difference		xx	
95% CI[c]		(xxx.x-xx.x)	
L guyanensis			
n (%)	xx (x.x)		xx (x.x)
Difference		xx	
95% CI[d]		(xxx.x-xx.x)	
L braziliensis			
n (%)	xx (x.x)		xx (x.x)
Difference		xx	
95% CI[e]		(xxx.x-xx.x)	
Difference p-value[a]		0.xxx	
Overall p-value[b]		0.xxx	

[a] c=uncorrected chi-square f=Fisher's exact test
[b] Cochran-Mantel-Haenszel test
<Program Name: Time and Date>

Table 52: Numbers and Proportion of Subjects Meeting Criteria for Final Clinical Cure of Index Lesions by Infecting Species Between Groups – Evaluable Subjects

	WR 279,396<br>n=xx		Paromomycin<br>n=xx
L panamensis			
n (%)	xx (x.x)		xx (x.x)
Difference		xx	
95% CI[f]		(xxx.x-xx.x)	
L guyanensis			
n (%)	xx (x.x)		xx (x.x)
Difference		xx	
95% CI[g]		(xxx.x-xx.x)	
L braziliensis			
n (%)	xx (x.x)		xx (x.x)
Difference		xx	
95% CI[h]		(xxx.x-xx.x)	
Difference p-value[a]		0.xxx	
Overall p-value[b]		0.xxx	

[a] c=uncorrected chi-square; f=Fisher's exact test
[b] Cochran-Mantel-Haenszel test
<Program Name: Time and Date>

Table 53: Numbers and Proportion of Index Lesions with Signs of Lesion Infiltration During the Study – mITT Subjects

Study Day	20	35	49	63	100	168
**Study Drug**						
**WR 279,396**						
**n=xx**	xx.x	xx.x	xx.x	xx.x	xx.x	xx.x
n (%)	(x.xx)	(x.xx)	(x.xx)	(x.xx)	(x.xx)	(x.xx)
**Paromomycin**						
**n=xx**	xx.x	xx.x	xx.x	xx.x	xx.x	xx.x
n (%)	(x.xx)	(x.xx)	(x.xx)	(x.xx)	(x.xx)	(x.xx)

<Program Name: Time and Date>

Table 54: Numbers and Proportion of Index Lesions with Signs of Lesion Infiltration During **the Study – Evaluable Subjects**

Study Day	20	35	49	63	100	168
**Study Drug**						
**WR 279,396**						
**n=xx**	xx.x	xx.x	xx.x	xx.x	xx.x	xx.x
n (%)	(x.xx)	(x.xx)	(x.xx)	(x.xx)	(x.xx)	(x.xx)
**Paromomycin**						
**n=xx**	xx.x	xx.x	xx.x	xx.x	xx.x	xx.x
n (%)	(x.xx)	(x.xx)	(x.xx)	(x.xx)	(x.xx)	(x.xx)

<Program Name: Time and Date>

Table 55: Cumulative Numbers of Subjects with New Lesions and Cumulative Number of New Lesions – mITT Subjects

Study Day	20	35	49	63	100	168
**Study Drug**						
**WR 279,396 n=xx**						
number of subjects	xx	xx	xx	xx	xx	xx
number of lesions	xx	xx	xx	xx	xx	xx
**Paromomycin n=xx**						
number of subjects	xx	xx	xx	xx	xx	xx
number of lesions	xx	xx	xx	xx	xx	xx

<Program Name: Time and Date>

Table 56: Cumulative Numbers of Subjects with New Lesions and Cumulative Number of New Lesions – Evaluable Subjects

Study Day	20	35	49	63	100	168
**Study Drug**						
**WR 279,396 n=xx**						
number of subjects	xx	xx	xx	xx	xx	xx
number of lesions	xx	xx	xx	xx	xx	xx
**Paromomycin n=xx**						
number of subjects	xx	xx	xx	xx	xx	xx
number of lesions	xx	xx	xx	xx	xx	xx

<Program Name: Time and Date>

Table 57: Cure Rates of New Lesions that Received Treatment – mITT Subjects

Day since start of treatment for new lesion	20	35	49	63	100	168
**Study Drug**						
**WR 279,396 n=xx**						
Number of new lesions	x	x	x	x	x	x
n (% with clinical cure)	x(xx.x)	x(xx.x)	x(xx.x)	x(xx.x)	x(xx.x)	x(xx.x)
**Paromomycin n=xx**						
Number of new lesions	x	x	x	x	x	x
n (% with clinical cure)	x(xx.x)	x(xx.x)	x(xx.x)	x(xx.x)	x(xx.x)	x(xx.x)

<Program Name: Time and Date>

Table 58: Cure Rates of New Lesions that Received Treatment – Evaluable Subjects

Day since start of treatment for new lesion	20	35	49	63	100	168
**Study Drug**						
**WR 279,396 n=xx**						
Number of new lesions	x	x	x	x	x	x
n (% with clinical cure)	x(xx.x)	x(xx.x)	x(xx.x)	x(xx.x)	x(xx.x)	x(xx.x)
**Paromomycin n=xx**						
Number of new lesions	x	x	x	x	x	x
n (% with clinical cure)	x(xx.x)	x(xx.x)	x(xx.x)	x(xx.x)	x(xx.x)	x(xx.x)

<Program Name: Time and Date>

Table 59: Overall Number of Adverse Events – Safety Subjects

	WR 279,396 (n=xx)	Paromomycin (n=xx)
Total number of AEs	Xx	xx
Total number (%) of mild AEs	xx (x.xx)	xx (x.xx)
Total number (%) of moderate AEs	xx (x.xx)	xx (x.xx)
Total number (%) of severe AEs	xx (x.xx)	xx (x.xx)
Total number (%) of life-threatening AEs	xx (x.xx)	xx (x.xx)
Total number (%) of subjects with mild AEs	xx (x.xx)	xx (x.xx)
Total number (%) of subjects with moderate AEs	xx (x.xx)	xx (x.xx)
Total number (%) of subjects with severe AEs	xx (x.xx)	xx (x.xx)
Total number (%) of subjects with life-threatening AEs	xx (x.xx)	xx (x.xx)

<Program Name: Time and Date>

Table 60: Summary of Adverse Events in WR 279,396 Group – Safety Subjects

System, Organ, Class/ Preferred Term	Number of Subjects (n, %) n=xx						
	Grade 1		Grade 2		Grade 3		Total
	related	unrelated	related	unrelated	related	unrelated	related + unrelated
**SOC No. 1**							
Preferred Term	x (x.x)	x (x.x)	x (x.x)	x (x.x)	x (x.x)	x (x.x)	x (x.x)
Preferred Term	x (x.x)	x (x.x)	x (x.x)	x (x.x)	x (x.x)	x (x.x)	x (x.x)
**SOC No. 2**							
Preferred Term	x (x.x)	x (x.x)	x (x.x)	x (x.x)	x (x.x)	x (x.x)	x (x.x)
Preferred Term	x (x.x)	x (x.x)	x (x.x)	x (x.x)	x (x.x)	x (x.x)	x (x.x)

Programmers Note: The categories of Definitely, Probably, and Possibly Related are considered investigational product related with regards to summary statistics. Multiple occurrences of a specific adverse event for a subject are counted once in the frequency for the adverse event. Likewise, multiple occurrences of adverse events within a specific preferred term for a subject are counted once in the frequency for the preferred term.

<Program Name: Time and Date>

Table 61: Summary of Adverse Events in Paromomycin Group – Safety Subjects

System, Organ, Class/ Preferred Term	Number of Subjects (n, %) n = xx						
	Grade 1		Grade 2		Grade 3		Total
	related	unrelated	related	unrelated	related	unrelated	related + unrelated
**SOC No. 1**							
Preferred Term	x (x.x)	x (x.x)	x (x.x)	x (x.x)	x (x.x)	x (x.x)	x (x.x)
Preferred Term	x (x.x)	x (x.x)	x (x.x)	x (x.x)	x (x.x)	x (x.x)	x (x.x)
**SOC No. 2**							
Preferred Term	x (x.x)	x (x.x)	x (x.x)	x (x.x)	x (x.x)	x (x.x)	x (x.x)
Preferred Term	x (x.x)	x (x.x)	x (x.x)	x (x.x)	x (x.x)	x (x.x)	x (x.x)

<Program Name: Time and Date>

Table 62: Summary of Adverse Events (Number and Percentage) Occurring in at Least 5% of Subjects in Any Group – Safety Subjects

Adverse Event Preferred Term	WR 279,396<br>n=xx	Paromomycin<br>n=xx
Preferred Term No. 1	n (%)	n (%)
Preferred Term No. 2	n (%)	n (%)

Table 63: Subjects with Evidence of Mucosal Erythema, Edema, Infiltration, or Erosion at Follow-up Visits During the Study

Preferred Term	WR 279,396<br>n=xx	Paromomycin<br>n=xx
**Day 63**		
Erythema	n (%)	n (%)
Edema	n (%)	n (%)
Infiltration	n (%)	n (%)
Erosion	n (%)	n (%)
None	n (%)	n (%)
**Day 100**		
Erythema	n (%)	n (%)
Edema	n (%)	n (%)
Infiltration	n (%)	n (%)
Erosion	n (%)	n (%)
None	n (%)	n (%)
**Day 168**		
Erythema	n (%)	n (%)
Edema	n (%)	n (%)
Infiltration	n (%)	n (%)
Erosion	n (%)	n (%)
None	n (%)	n (%)

Table 64: Adverse Events Time to Onset – Safety Subjects

SOC/Preferred Term	WR 279,396<br>n=xx	Paromomycin<br>n=xx
SOC No. 1		
Preferred Term No. 1		
n	xx	xx
Mean (SD)	xx.x (x.xx)	xx.x (x.xx)
Median	xx.x	xx.x
Range	xx-xx	xx-xx

<Program Name: Time and Date>

Table 65: Adverse Events Duration – Safety Subjects

SOC/Preferred Term	WR 279,396<br>n=xx	Paromomycin<br>n=xx
SOC No. 1		
Preferred Term No. 1		
n	xx	xx
Mean (SD)	xx.x (x.xx)	xx.x (x.xx)
Median	xx.x	xx.x
Range	xx-xx	xx-xx

<Program Name: Time and Date>

Table 66: Clinical Chemistry: Day 1 and 20 – Safety Subjects

Study Drug	WR 279,396 n=xx			Paromomycin n=xx		
**Study Day**	**1**	**20**	**Change from Baseline**	**1**	**20**	**Change From Baseline**
Creatinine (mg/dL)						
n	xx	xx	xx	xx	xx	xx
Mean	xx.x	xx.x	xx.x	xx.x	xx.x	xx.x
SD	x.xx	x.xx	x.xx	x.xx	x.xx	x.xx
Median	xx.x	xx.x	xx.x	xx.x	xx.x	xx.x
Range	xx-xx	xx-xx	xx-xx	xx-xx	xx-xx	xx-xx
AST (U/L)						
n	xx	xx	xx	xx	xx	xx
Mean	xx.x	xx.x	xx.x	xx.x	xx.x	xx.x
SD	x.xx	x.xx	x.xx	x.xx	x.xx	x.xx
Median	xx.x	xx.x	xx.x	xx.x	xx.x	xx.x
Range	xx-xx	xx-xx	xx-xx	xx-xx	xx-xx	xx-xx
ALT (U/L)						
n	xx	xx	xx	xx	xx	xx
Mean	xx.x	xx.x	xx.x	xx.x	xx.x	xx.x
SD	x.xx	x.xx	x.xx	x.xx	x.xx	x.xx
Median	xx.x	xx.x	xx.x	xx.x	xx.x	xx.x
Range	xx-xx	xx-xx	xx-xx	xx-xx	xx-xx	xx-xx

<Program Name: Time and Date>

Table 67: Number (%) of Subjects with Laboratory Values above the Upper Limit of Normal – Safety Subjects

	WR 279,396<br>n=xx	Paromomycin<br>n=xx
**Creatinine - Screening**		
n (%)	xx (x.x)	xx (x.x)
**Creatinine - Day 20**		
n (%)	xx (x.x)	xx (x.x)
**AST – Screening**		
n (%)	xx (x.x)	xx (x.x)
**AST - Day 20**		
n (%)	xx (x.x)	xx (x.x)
**ALT – Screening**		
n (%)	xx (x.x)	xx (x.x)
**ALT - Day 20**		
n (%)	xx (x.x)	xx (x.x)

<Program Name: Time and Date>

Table 68: Pregnancy Test Results

	WR 279,396<br>n=xx	Paromomycin<br>n=xx
**Screening**		
n (%) negative	xx (x.x)	xx (x.x)
n (%) positive	xx (x.x)	xx (x.x)
**Day 35**		
n (%) negative	xx (x.x)	xx (x.x)
n (%) positive	xx (x.x)	xx (x.x)

Table 69: Summary of Concomitant Medication Use (Number and Percentage of Subjects) – Safety Subjects

Medication Preferred Term	WR 279,396<br>n=xx	Paromomycin<br>n=xx
Preferred Term No. 1	xx.x (x.xx)	xx.x (x.xx)
Preferred Term No. 2	xx.x (x.xx)	xx.x (x.xx)

13. Appendix B: Figure Shells

Figure 1: Mean Area of Ulceration of the Index Lesion at All Study Time Points – mITT Subjects. 70

Figure 2: Mean Area of Ulceration of the Index Lesion at All Study Time Points – Evaluable Subjects 70

Figure 3: Index Lesion Cure Rate at Each Measurement Time Point – mITT Subjects........ 71

Figure 4: Index Lesion Cure Rate at Each Measurement Time Point – Evaluable Subjects 71

Figure 5: Time to Initial Clinical Cure of Index lesion – mITT Subjects........ 72

Figure 6: Time to Initial Clinical Cure of Index Lesion – Evaluable Subjects........ 72

Figure 7: Proportion of Subjects with New Lesions Over Time – mITT Subjects........ 73

Figure 8: Number of New Lesions Over Time – mITT Subjects 73

Figure 1: Mean Area of Ulceration of the Index Lesion at All Study Time Points – mITT Subjects

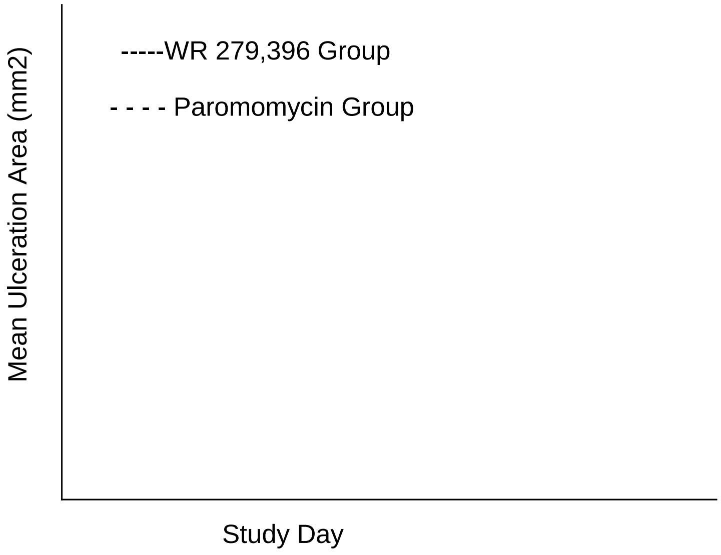

Figure 2: Mean Area of Ulceration of the Index Lesion at All Study Time Points – Evaluable Subjects

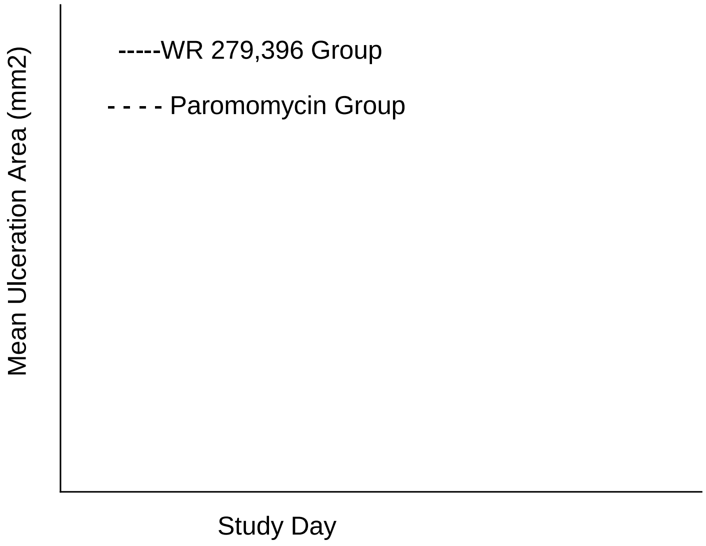

Figure 3: Index Lesion Cure Rate at Each Measurement Time Point – mITT Subjects

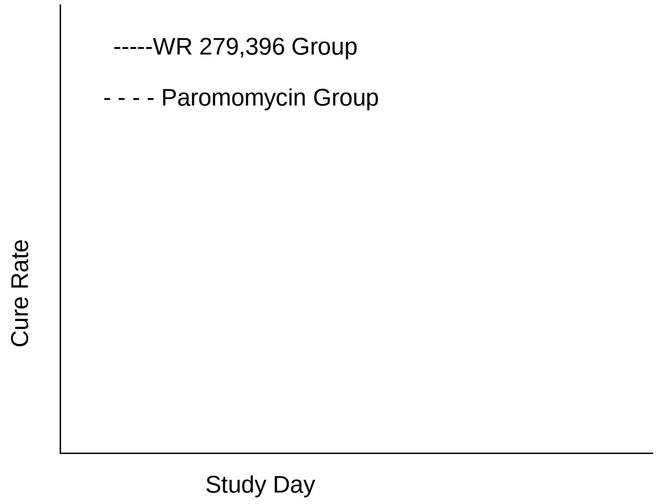

Figure 4: Index Lesion Cure Rate at Each Measurement Time Point – Evaluable Subjects

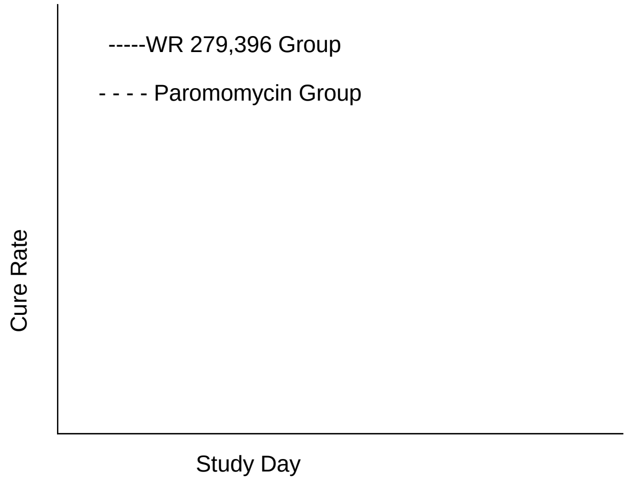

Figure 5: Time to Initial Clinical Cure of Index lesion – mITT Subjects

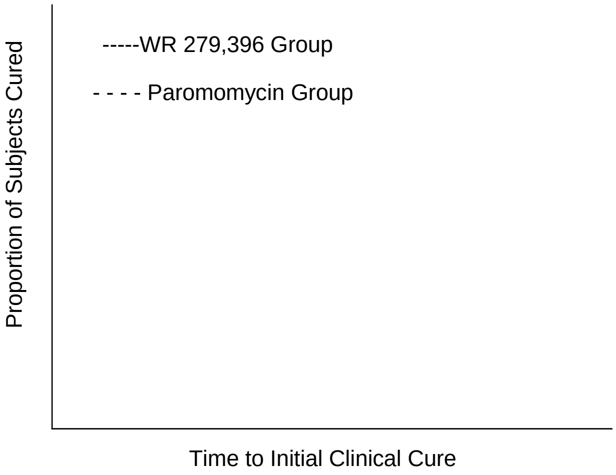

Figure 6: Time to Initial Clinical Cure of Index Lesion – Evaluable Subjects

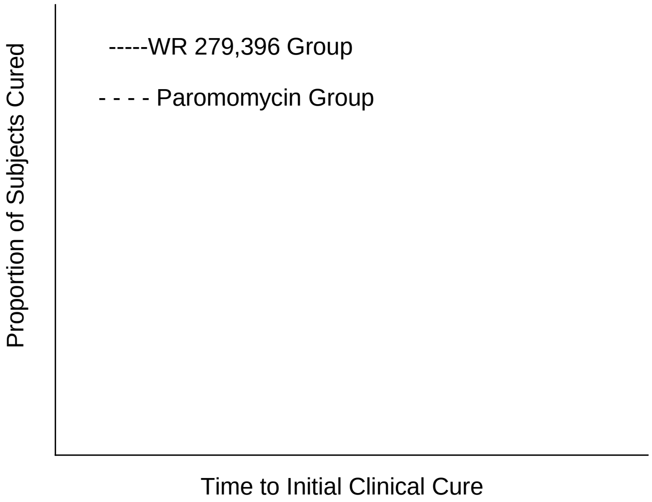

Figure 7: Proportion of Subjects with New Lesions Over Time – mITT Subjects

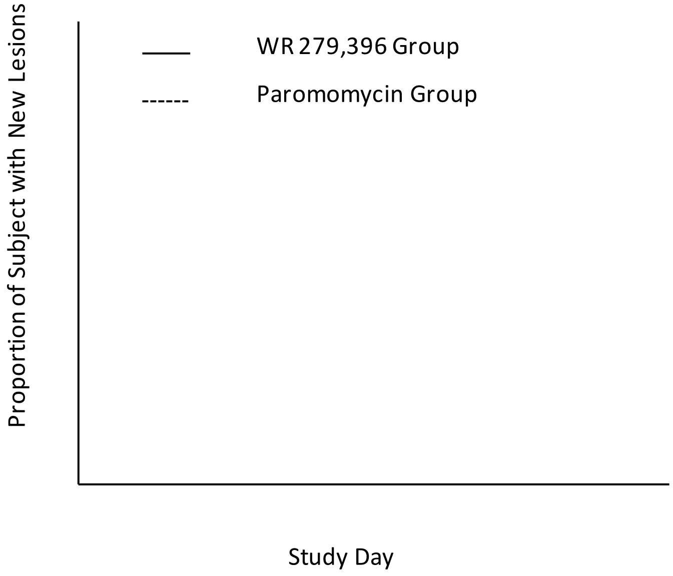

Figure 8: Number of New Lesions Over Time – mITT Subjects

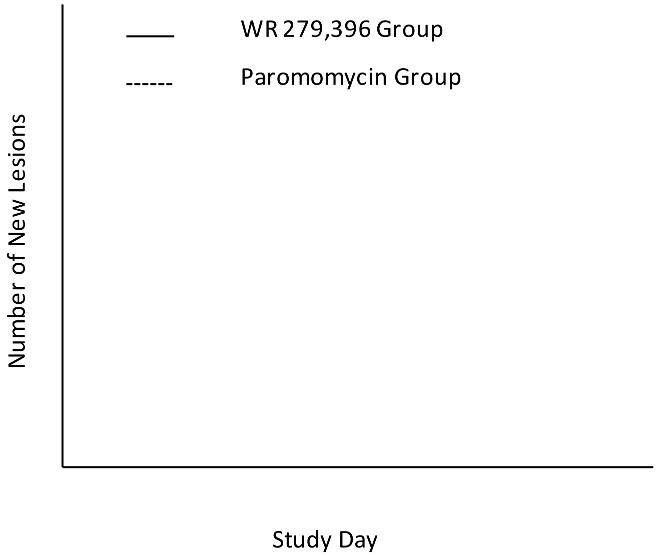

14. Appendix C: Listing Shells

Listing 1. Subject Disposition – All Randomized Subjects 75
Listing 2 *continued* Subject Disposition – All Randomized Subjects 75
Listing 3. Reasons for Not Meeting Eligibility Criteria – All Subjects 76
Listing 4. Protocol Deviations – All Subjects 76
Listing 5. Demographics – mITT Subjects 77
Listing 6. Baseline Medical History – mITT Subjects 77
Listing 7. Baseline Physical Exam – mITT Subjects 78
Listing 8. Vital Signs – mITT Subjects 79
Listing 9. Pregnancy Test Results – mITT Subjects 79
Listing 10. Baseline Lesion Types – mITT Subjects 79
Listing 11. Parasitology Results – mITT Subjects 80
Listing 12. Final Clinical Cure of Index Lesion – mITT Subjects 81
Listing 13. Final Clinical Cure of All Lesions – mITT Subjects 81
Listing 14. Area of Ulceration of Lesions at All Assessments –mITT Subjects 82
Listing 15. New Lesion Assessments – mITT Subjects 82
Listing 16. Cure Status of All Lesions at All Study Time Points – mITT Subjects 83
Listing 17. Study Drug Application: Days 1 - 20 – mITT Subjects 83
Listing 18. Study Drug Application: New Lesions (After Day 20) – mITT Subjects 84
Listing 19. Adverse Events – mITT Subjects 84
Listing 20. Adverse Events Leading to Withdrawal – mITT Subjects 85
Listing 21. Serious Adverse Events – mITTS Subjects 86
Listing 22. Clinical Laboratory Results Listings – mITT Subjects 88
Listing 23. Unscheduled Clinical Laboratory Results Listings – mITT Subjects 88
Listing 24. Physical Exam of Mucosa – mITT Subjects 89
Listing 25. Concomitant Medications – mITT Subjects 89

Listing 1. Subject Disposition – All Randomized Subjects

Group	Subject ID No.	Consent Date	Randomization Date	Completion Date	mITT Subject	Evaluable Subject	Completed Study Through Day 168?
WR 279,396	xxx	dd/mmm/yyyy	dd/mmm/yyyy	dd/mmm/yyyy	Y	Y	Y
Paromomycin					N	N	N

Programming Note: Sorted by Group, Subject ID.

<Program Name: Time and Date>

Listing 2 *continued* Subject Disposition – All Randomized Subjects

Group	Subject ID No.	Reasons for Early Discontinuation	Reason Detail
WR 279,396	xxx	Clinical evidence of continued presence of disease	
Paromomycin		Worsening of disease	
		Recurrence of disease	
		AE/SAE	
		Death	
		Withdrawal of subject by investigator	Verbatim text
		Withdrawal of consent by Subject	
		Lost to follow-up	
		Other	Verbatim text

Programming Note: Sorted by Group, Subject ID.

<Program Name: Time and Date>

Listing 3. Reasons for Not Meeting Eligibility Criteria – All Subjects

Subject ID No.	Reason for Screen Failure	Other Description
Xxx	Subject withdrew consent	
	Negative parasitology	
	Had recent(<56 days) CL treatment	
	Pregnant	
	Breastfeeding	
	>10 lesions	
	Significant organ abnormality	
	Hypersensitivity to aminoglycosides	
	Evidence of mucosal disease	
	Contraindicated medical condition	
	Other:	Verbatim text

Programming Note: Subject ID.
<Program Name: Time and Date>

Listing 4. Protocol Deviations – All Subjects

Group	Subject ID No.	Deviation Date	Deviation Type	Deviation Detail
WR 279,396	xxx	dd/mmm/yyyy	I/E[a] EXCEPTION	Verbatim text
Paromomycin			VISIT DEVIATION	
			MISSED VISIT	
			OTHER	

[a] I/E=Inclusion/Exclusion
Note: Only subjects with protocol deviations are listed.
Programming Note: Sorted by Group, Subject ID.
<Program Name: Time and Date>

Listing 5. Demographics – mITT Subjects

Group	Subject ID No.	Age (years at Date of Consent)	Gender	Race	Ethnicity
WR 279,396	xxx		M[a]	Mestizo	Hispanic or Latino
Paromomycin			F[b]	Other	Not Hispanic of Latino
					Not given

[a] M=male
[b] F=female
Programming Note: Sorted by Group, Subject ID. Insert verbatim when “Other” race is selected.
<Program Name: Time and Date>

Listing 6. Baseline Medical History – mITT Subjects

Group	Subject ID No.	Evaluation Date	History Verbatim Term/MedDRA PT[a]/SOC[b]	Onset Date	Start Day (relative to start of treatment)	Condition Present Currently?
WR 279,396	xxx	dd/mmm/yyyy	xxxx/xxxx/xxxx	dd/mmm/yyyy	xxx	Y
Paromomycin						N

[a] PT=Preferred Term
[b] SOC=System Order Class
Programming Note: Sorted by Group, Subject ID, System Order Class, MedDRA PT.
<Program Name: Time and Date>

Listing 7. Baseline Physical Exam – mITT Subjects

Group	Subject ID No.	Exam Date	Visit	Body System	Abnormal Finding Description
WR 279,396	xxx	dd/mmm/yyyy	Screening	Oral Cavity	Verbatim text
Paromomycin			Unscheduled	Head	
				Eyes	
				Ears-Nose-Throat	
				Cardiovascular	
				Lungs	
				Abdomen	
				Extremities	
				Skin	
				Lymphatics	
				Neuropsychiatric	
				Sensor/Motor Status	
				Musculoskeletal	
				General Appearance	
				Other	

Programming Note: Sorted by Group, Subject ID, Body System. Only list abnormal findings.
<Program Name: Time and Date>

Listing 8. Vital Signs – mITT Subjects

Group	Subject ID No.	Exam Date	Study Day	Temperature (˚C)	Heart rate (Beats/Minute)	Systolic Blood Pressure (mmHg)	Diastolic Blood Pressure (mmHg)
WR 279,396	xxx	dd/mmm/yyyy	Screening	xxx.x	xxx	xxx	Xxx
Paromomycin			Unscheduled				

Programming Note: Sorted by Group, Subject ID, Exam Date.
<Program Name: Time and Date>

Listing 9. Pregnancy Test Results – mITT Subjects

Group	Subject ID No.	Study Day	Date of Test	Pregnancy Test Result
WR 279,396	xxx	Screening	dd/mmm/yyyy	Negative
Paromomycin		Day 1/-1		Positive
		Day 35		NA
				ND

Programming Note: Sorted by Group, Subject ID.
<Program Name: Time and Date>

Listing 10. Baseline Lesion Types – mITT Subjects

Group	Subject ID No.	Index Lesion No.	Lesion No.	Body Site	Primary Lesion Type	Secondary Infection at Treatment Site?
WR 279,396	xxx	xx	01…10	xx	ULCERATED	Y
Paromomycin					NONULCERATED	N

Programming Note: Sorted by Group, Subject ID, Lesion No., Evaluation Date.
<Program Name: Time and Date

Listing 11. Parasitology Results – mITT Subjects

Group	Subject ID No.	Date of Collection	Lesion No.	Microscopic Evaluation	Culture Evaluation	Species by Isoenzyme Analysis	Species by PCR/RFLP
WR 279,396	xxx	dd/mmm/yyyy	xx	NEGATIVE	POSITIVE	*L. panamensis*	*L. panamensis*
Paromomycin				POSITIVE	NEGATIVE	*L. braziliensis*	*L. braziliensis*
				NOT DONE	NOT DONE	Not Done	Unknown
						Not Applicable	Not Done

Abbreviations: PCR/RFLP=Polymerase Chain Reaction
Programming Note: Sorted by Group, Subject ID, Lesion No.
<Program Name: Time and Date>

Listing 12. Final Clinical Cure of Index Lesion – mITT Subjects

Group	Subject ID No.	Evaluable	Met Criteria for Final Clinical Cure?
WR 279,396	xxx	Y	Y
Paromomycin		N	N

Programming Note: Sorted by Group, Subject ID.
<Program Name: Time and Date>

Listing 13. Final Clinical Cure of All Lesions – mITT Subjects

Group	Subject ID No.	Evaluable	Met Criteria for Final Clinical Cure?
WR 279,396	xxx	Y	Y
Paromomycin		N	N

Programming Note: Sorted by Group, Subject ID.
<Program Name: Time and Date>

Listing 14. Area of Ulceration of Lesions at All Assessments –mITT Subjects

Group	Subject ID No.	Evaluation Date	Study Day	Lesion No.	Index Lesion	Length (mm)	Width (mm)	Area (mm^2)	Secondary Infection at treatment site	Infiltration
WR 279,396	xxx	dd/mmm/yyyy	1	01…10	Y	xxx.x	xxx.x	xxx.x	Y	Y
Paromomycin			20		N				N	N
			35							
			49							
			63							
			100							
			168							

Programming Note: Sorted by Group, Subject ID, Lesion No., Visit.
<Program Name: Time and Date>

Listing 15. New Lesion Assessments – mITT Subjects

Group	Subject ID No.	Were New Lesions Observed?	Lesion No.	Body Site No.	Date of Appearance	Evaluation Date	Study Day	Length (mm)	Width (mm)	Area (mm^2)
WR 279,396	xxx	Y	xx	xx	dd/mmm/yyyy	dd/mmm/yyyy	xxx	xxx.x	xxx.x	xxx.x
Paromomycin		N								

Programming Note: Sorted by Group, Subject ID, Lesion No., Date.
<Program Name: Time and Date>

Listing 16. Cure Status of All Lesions at All Study Time Points – mITT Subjects

Group	Subject ID No.	Study Day	Date	Lesion No.	Lesion Type	Cured?	Secondary Infection at treatment site?
WR 279,396	xxx	xx	dd/mmm/yyyy	xx	Ulcerated	Y	Y
Paromomycin					Non-ulcerated	N	N
						UNKNOWN	UNKNOWN

Programming Note: Sorted by Group, Subject ID,Lesion No., Date
<Program Name: Time and Date>

Listing 17. Study Drug Application: Days 1 - 20 – mITT Subjects

Group	Subject ID No.	First Day of Treatment	Last Day of Treatment	Missed Treatments?	What Days?	Total Amount Applied (g)	Number of Lesions
WR 279,396	xxx	dd/mmm/yyyy	dd/mmm/yyyy	Y	xxxxxx	xxx.x	xx
Paromomycin				N			

Programming Note: Sorted by Group, Subject ID.
<Program Name: Time and Date>

Listing 18. Study Drug Application: New Lesions (After Day 20) – mITT Subjects

Group	Subject ID No.	New Lesions?	Start of Treatment	End of Treatment	Amount Applied (g)
WR 279,396	xxx	Y	dd/mmm/yyyy	dd/mmm/yyyy	xxx.x
Paromomycin		N			

Programming Note: Sorted by Group, Subject ID.
<Program Name: Time and Date>

Listing 19. Adverse Events – mITT Subjects

Group	Subject ID No.	AE Name/ MedDRA PT/ SOC	Onset Date/ Stop Date	Onset Day	Outcome	Relationship	Action with respect to study drug	Severity	Treatment Required	Serious
WR 279,396	xxx	xxxx/ xxxx / xxxx	dd/mmm/yyyy dd/mmm/yyyy	xx	Resolved	Not Related	None	Mild	N	N
Paromomycin					Recovered with sequelae	Unlikely	Temporarily discontinued	Moderate	Y	Y
					Ongoing	Possibly	Permanently discontinued	Severe		
					Unknown	Probably		Potentially life-threatening		
						Definitely				

Programming Note: Sorted by Group, Subject ID, Onset Date.
<Program Name: Time and Date>

Listing 20. Adverse Events Leading to Withdrawal – mITT Subjects

Group	Subject ID No.	AE Name/ MedDRA PT/ SOC	Onset Date/ Stop Date	Onset Day	Outcome	Relationship	Action with respect to study drug	Severity	Treatment Required	Serious
WR 279,396	xxx	xxxx/ xxxx / xxxx	dd/mmm/yyyy dd/mmm/yyyy	xx	Resolved	Not Related	None	Mild	N	N
Paromomycin					Recovered with sequelae	Unlikely	Temporarily discontinued	Moderate	Y	Y
					Ongoing	Possibly	Permanently discontinued	Severe		
					Unknown	Probably		Potentially life-threatening		
						Definitely				

Programming Note: Sorted by Group, Subject ID, Onset Date.
<Program Name: Time and Date>

Listing 21. Serious Adverse Events – mITTS Subjects

Group	Subject ID No.	Report Type	Date of Birth	Gender	SAE Name	SAE Description	Considered serious because	Onset Date/ Stop Date	Death Date
WR 279,396	xxx	Initial	dd/mmm/yyyy	M[a]	Verbatim text	Verbatim text	Death	dd/mmm/yyyy dd/mmm/yyyy	dd/mmm/yyyy
Paromomycin		Follow-up		F[b]			Life-threatening		
		Final					Hospitalization-initial or prolonged		
							Disability or permanent damage		
							Congenital anomalies or birth defects		
							Other medically important condition		
							Required intervention to prevent permanent damage		

[a] M=Male
[b] F=Female
Programming Note: Sorted by Group, Subject ID.
<Program Name: Time and Date>

Listing 20. Serious Adverse Events – mITT Subjects (continued)

Group	Subject ID No.	SAE Name	Date Hospitalized	Discharge Date	Continued Study Participation	Continued Study Drug
WR 279,396	xxx	Verbatim text	dd/mmm/yyyy	dd/mmm/yyyy	Y	Y
Paromomycin					N	N

Programming Note: Sorted by Group, Subject ID
<Program Name: Time and Date>

Listing 20. Serious Adverse Events – mITT Subjects (continued)

Group	Subject ID No.	SAE Name	Date of First Treatment of Study Drug	Date of Last Treatment of Study Drug	Time of Last Treatment of Study Drug	Event Abated After Study Drug Stopped	Event Reappeared After Reintroduction?	Relationship to Study Drug
WR 279,396	xxx	Verbatim text	dd/mmm/yyyy	dd/mmm/yyyy	hh:mm	Y	Y	Definite
Paromomycin	xxx					N	N	Probable
								Possible
								Unlikely
								Not Related

Programming Note: Sorted by Group, Subject ID
<Program Name: Time and Date>

Listing 22. Clinical Laboratory Results Listings – mITT Subjects

Group	Subject ID No.	Study Day	Date	Analyte	Analyte Units	Test Results	Evaluation	Lab Normal Range
WR 279,396	xxx	Screening	dd/mmm/yyyy	Creatinine	mg/dL	xx.x	WNL[a]	xx-xx
Paromomycin	xxx	Day 20		AST	U/L	xxx	Abnormal, NCS[b]	xx-xx
		Unscheduled		ALT	U/L	xxx	Abnormal, CS[c]	xx-xx
							Not Done	

[a] Within normal limits
[b] Not clinically significant
[c] Clinically significant
Programming Note: Sorted by Group, Subject ID, Analyte, Study Day
<Program Name: Time and Date>

Listing 23. Unscheduled Clinical Laboratory Results Listings – mITT Subjects

Group	Subject ID No.	Study Day	Date	Analyte	Analyte Units	Test Results	Evaluation
WR 279,396	xxx	Unscheduled	dd/mmm/yyyy	xxxxx	xxxxx	xxxxx	WNL[a]
Paromomycin							Abnormal, NCS[b]
							Abnormal, CS[c]
							Not Done

[a] Within normal limits
[b] Not clinically significant
[c] Clinically significant
Programming Note: Sorted by Group, Subject ID, Analyte, Study Day
<Program Name: Time and Date>

Listing 24. Physical Exam of Mucosa – mITT Subjects

Group	Subject ID No.	Visit	Examination Date	Body System	Signs of Disease	Present
WR 279,396	xxx	Screening	dd/mmm/yyyy	Nasal Skin	Erythema	Y
Paromomycin		Day 63		Nasal Mucosa	Edema	N
		Day 100		Palate	Infiltration	ND
		Day 168		Pharynx	Erosion	
		Unscheduled		Larynx	None	
					Not Done	

Programming Note: Sorted by Group, Subject ID, Body System, Visit
<Program Name: Time and Date>

Listing 25. Concomitant Medications – mITT Subjects

Group	Subject ID No.	Prior Med?	Verbatin Med Name/ PT[a] /ATC[b] Code/ Class/	Indication	Route	Dose	Frequency	Start Date	Stop Date
WR 279,396	xxx	YES	xxx/<br>xxxx/<br>xxxx/<br>xxxxxx	xxxxxx	xxxxxx	xxxxxx	xxxxxx	dd/mmm/yyyy	dd/mmm/yyyy
Paromomycin		NO							Ongoing

[a] PT=Preferred Term
[b] ATC=Anatomic, Therapeutic, and Chemical
Concomitant medications will be coded to a drug term using the WHO Drug Dictionary (recent version).
Programming Note: Sorted by Group, Subject ID, Start Date.
<Program Name: Time and Date>
